# Supplementary material for: Carfilzomib, lenalidomide, dexamethasone, and cyclophosphamide (KRdc) as induction therapy for transplant-eligible, newly diagnosed multiple myeloma patients (Myeloma XI+): Interim analysis of an open-label randomised controlled trial
Source: PLoS Med. 2021 Jan 11;18(1):e1003454. doi: 10.1371/journal.pmed.1003454 (PMC7799846; doi:10.1371/journal.pmed.1003454)
Supplement: S1 Text — Fig A—Progression-free survival by cytogenetic risk. Progression-free survival (PFS) for carfilzomib, lenalidomide, dexamethasone, and cyclophosphamide (KRdc) compared to the triplet control group (Rdc/Tdc) within each cytogenetic risk group. (A) Standard risk, (B) high risk, (C) ultra-high risk. Adverse molecular abnormalities were defined as gain(1q), del(17p), t(4;14), t(14;16), or t(14;20). Efficacy in the subgroups of standard risk, high risk (defined as 1 adverse cytogenetic abnormality) and ultra-high risk (2 or more adverse cytogenetic abnormalities) were pre-specified by protocol. CI, confidence interval; HR, hazard ratio; m, months. Fig B—Progression-free survival for patients with/without t(4;14) and del(17p). Progression-free survival (PFS) for carfilzomib, lenalidomide, dexamethasone, and cyclophosphamide (KRdc) compared to the triplet control group (Rdc/Tdc) for patients with the cytogenetic lesions t(4;14) and del(17p). (A) t(4;14), (B) no t(4;14), (C) del(17p), (D) no del(17p). CI, confidence interval; HR, hazard ratio; m, months. Fig C—Progression-free survival adjusted for CVD randomisation. Progression-free survival (PFS) for carfilzomib, lenalidomide, dexamethasone, and cyclophosphamide (KRdc) compared to the triplet control group (Rdc/Tdc) with patients achieving a suboptimal response to triplet treatment and randomised to no intensification therapy removed. CI, confidence interval; HR, hazard ratio; m, months. Table A—Myeloma XI+ study sites and principal investigators. Text A—CONSORT Checklist. Text B—Myeloma XI+ protocol. (PDF) [file pmed.1003454.s001.pdf]

S1 Text: Supplementary Appendix

Carfilzomib, Lenalidomide, Dexamethasone and Cyclophosphamide (KRdc) as Induction Therapy for Transplant Eligible Newly Diagnosed Multiple Myeloma Patients (Myeloma XI+): interim analysis of an open-label randomised controlled trial.

Contents

Supplementary Figures
- Figure A – Progression-free Survival by Cytogenetic Risk 2
- Figure B – Progression-free Survival for Patients with/without t(4;14) and del(17p) 3
- Figure C – Progression-free Survival Adjusted for CVD Randomisation ..4

Supplementary Tables
- Table A - Myeloma XI+ Study Sites and Principal Investigators 5

Supplementary Text
- Text A - CONSORT Checklist..8
- Text B – Myeloma XI+ Protocol 12

Supplementary Figures

Supplementary Figure A – Progression-free Survival by Cytogenetic Risk

Progression-free survival (PFS) for carfilzomib, lenalidomide, dexamethasone cyclophosphamide (KRdc) compared to the triplet control group (Rdc/Tdc) within each cytogenetic risk group. A) Standard risk, B) High-risk, C) Ultra high-risk. Adverse molecular abnormalities were defined as gain(1q), del(17p), t(4;14), t(14;16) or t(14;20). Efficacy in the subgroups of standard risk, high-risk (defined as one adverse cytogenetic abnormality) and ultra-high-risk cytogenetic profile (two or more adverse cytogenetic abnormalities) were prespecified by protocol. HR, hazard ratio; CI, confidence interval; m, months.

A)

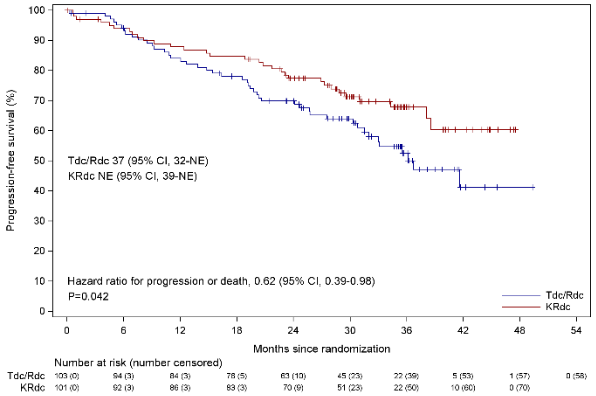

B)

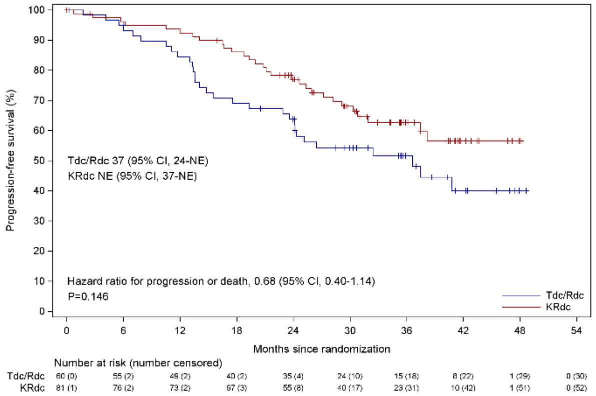

C)

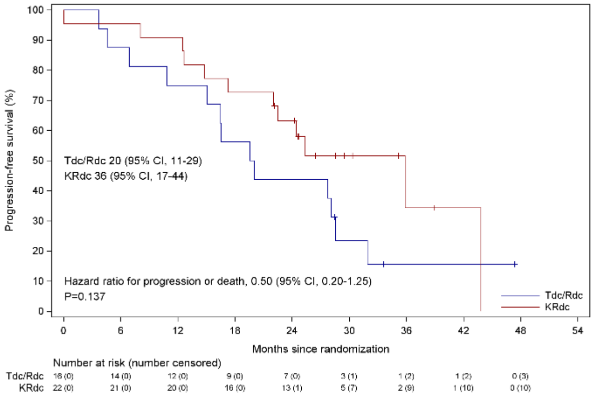

Supplementary Figure B – Progression-free Survival for Patients with/without t(4;14) and del(17p)

Progression-free survival (PFS) for carfilzomib, lenalidomide, dexamethasone cyclophosphamide (KRdc) compared to the triplet control group (Rdc/Tdc) for patients with the cytogenetic lesions t(4;14) and del(17p). A) t(4;14), B) no t(4;14), C) del(17p) D) no del(17p). HR, hazard ratio; CI, confidence interval; m, months.

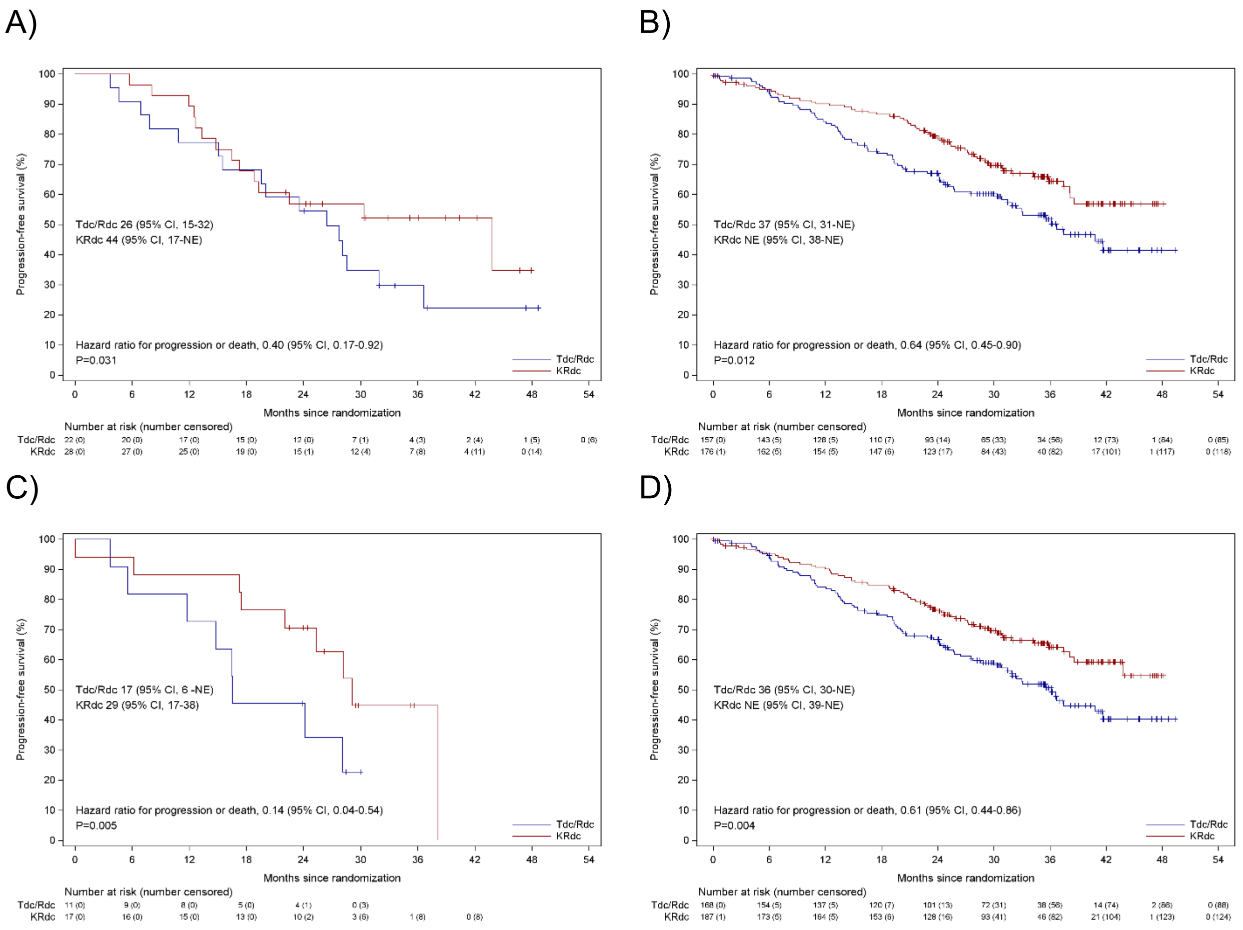

Supplementary Figure C – Progression-free Survival Adjusted for CVD Randomisation
Progression-free survival (PFS) for carfilzomib, lenalidomide, dexamethasone cyclophosphamide (KRdc) compared to the triplet control group (Rdc/Tdc) with patients achieving a suboptimal response to triplet and randomised to no intensification therapy removed. HR, hazard ratio; CI, confidence interval; m, months.

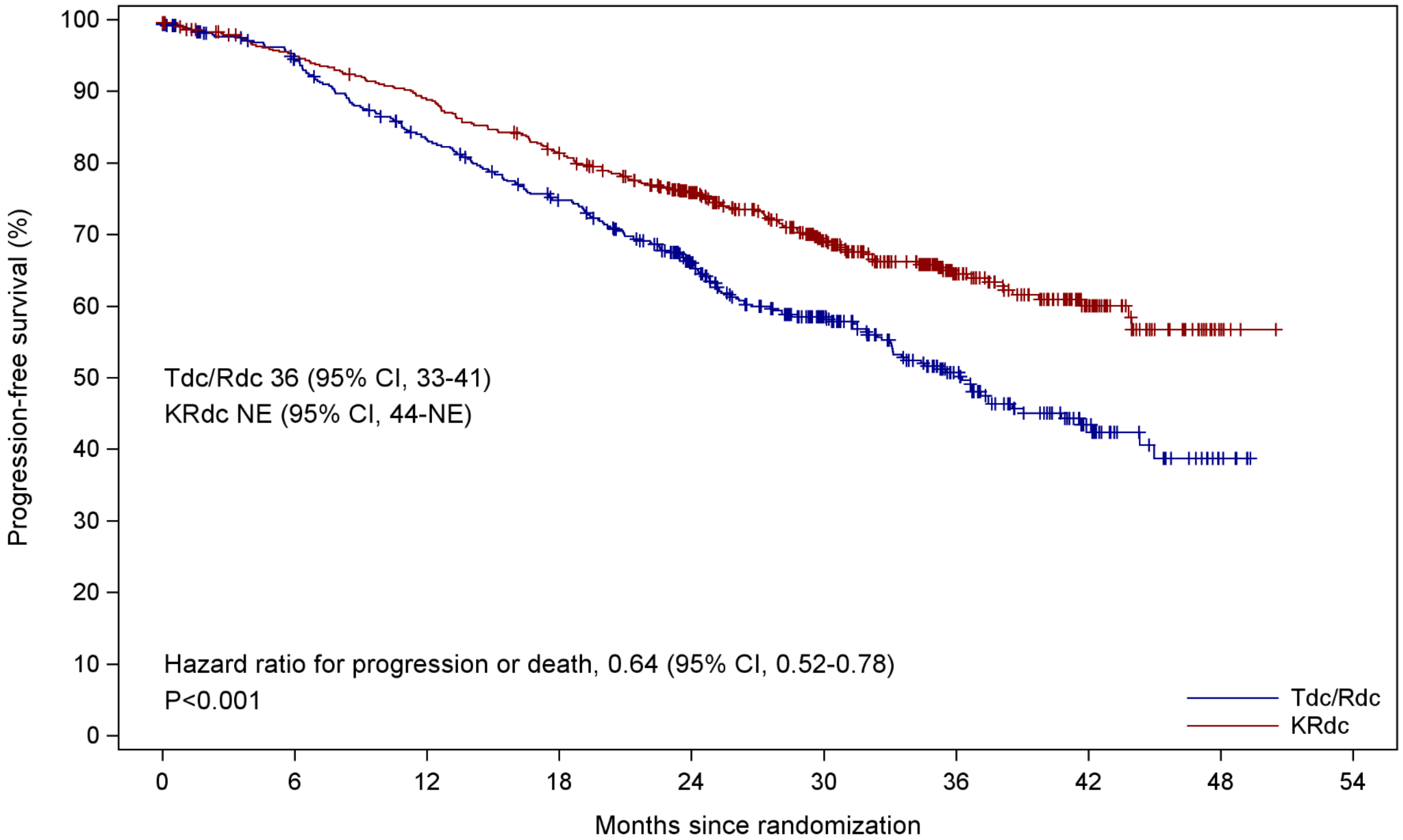

Number at risk (number censored)

	0	6	12	18	24	30	36	42	48	54
Tdc/Rdc	485 (5)	437 (22)	379 (28)	333 (36)	261 (70)	180 (123)	100 (183)	43 (228)	7 (261)	0 (268)
KRdc	526 (7)	484 (17)	451 (18)	410 (21)	337 (67)	237 (140)	129 (235)	62 (295)	5 (350)	0 (355)

Table A - Myeloma XI+ Study Sites and Principal Investigators

Site	Principal Investigator(s)	Recruited patients
Royal Stoke University Hospital, Stafford County Hospital (University Hospital North Staffordshire)	Dr Kamaraj Karunanithi, Dr Paul Revell	43
Leicester Royal Infirmary	Dr Mamta Garg, Dr Claire Chapman	37
Southampton General Hospital	Dr Matthew Jenner, Dr Alastair Smith	31
Manchester Royal Infirmary, Trafford General Hospital	Dr Alberto Rocci, Dr Eleni Tholouli, Dr John Alderson, Dr Simon Gibbs	28
Royal Hallamshire Hospital, Sheffield	Prof John Snowden	27
Monklands Hospital, Hairmyres Hospital, Wishaw General Hospital	Dr Iain Singer	26
Singleton Hospital, Swansea	Dr Hamdi Sati	26
Nottingham City Hospital	Dr Cathy Williams, Prof Nigel Russell	24
Worcestershire Royal Hospital, Alexandra Hospital Redditch, Kidderminster General Hospital	Dr Salim Shafeek	23
University Hospital Coventry	Dr Beth Harrison, Dr Syed Bokhari	22
Western General Hospital, Edinburgh	Dr Huw Roddie	21
James Cook University Hospital, Middlesbrough	Dr Raymond Dang	20
Beatson Oncology Centre, Glasgow	Dr Richard Soutar	19
New Cross Hospital, Wolverhampton	Dr Supratik Basu	19
Norfolk and Norwich University Hospital	Dr Martin Auger, Dr Kristian Bowles	19
Derriford Hospital, Plymouth	Dr Hannah Hunter	18
Lincoln County Hospital, Grantham and District General Hospital, Pilgrim Hospital Boston	Dr Caroline Harvey, Dr Charlotte Kallmeyer, Dr Kandeepan Saravanmuttu	18
Medway Maritime Hospital	Dr Sarah Arnott, Dr Vijay Dhanapal, Dr Vivienne Andrews	18
Aberdeen Royal Infirmary	Dr Jane Tighe	17
Castle Hill Hospital, Hull	Dr David Allsup, Dr Haz Sayala	17
Ipswich Hospital	Dr Isobel Chalmers	17
Royal Cornwall Hospital, Truro	Dr Julie Blundell	17
University Hospital of Wales Cardiff, Llandough Hospital	Dr Ceri Bygrave, Dr Christopher Fegan, Dr Belinda Austin	17
Birmingham Heartlands Hospital, Good Hope Hospital	Dr Bhuvan Kishore, Prof Donald Milligan	16
Ninewells Hospital Dundee, Perth Royal Infirmary	Dr Duncan Gowans	16
Chesterfield Royal Hospital	Dr Peter Toth, Dr Emma Welch	15
Royal Bournemouth Hospital	Dr Rachel Hall	15
Royal Devon and Exeter Hospital	Dr Tony Todd, Dr Claudius Rudin	15
Bristol Haematology and Oncology Centre	Dr Jenny Bird, Dr Roger Evely	14
Freeman Hospital, Newcastle	Prof Graham Jackson	14
Royal Preston Hospital	Dr Mark Grey, Dr Frederick Kanyike, Dr Maqsood Punekar	14

University Hospital Aintree	Dr Lynny Yung, Dr Barbara Hammer	14
Cheltenham General Hospital, Gloucestershire Royal Hospital	Dr Sally Chown	13
Royal Berkshire Hospital, Reading	Dr Henri Grech	13
Worthing Hospital, St Richards Hospital Chichester	Dr Jamie Wilson, Dr Sarah Janes, Dr Phillip Bevan, Dr Santosh Narat	13
Blackpool Victoria Hospital	Dr Mark Grey, Dr Marian Paul Macheta	12
Diana Princess of Wales Hospital, Grimsby	Dr Susan Levison-Keating, Dr Sanjeev Jalihal, Dr Hannah Ciepluch	12
Royal Derby Hospital	Dr David Allotey	12
Scunthorpe General Hospital	Dr Sanjeev Jalihal	12
Southmead Hospital, Bristol (Frenchay)	Dr Alastair Whiteway	12
Victoria Hospital Kirkcaldy	Dr Lorna McClintock	12
Calderdale Royal Hospital, Huddersfield Royal Infirmary	Dr Kate Rothwell, Dr Sylvia Feyler	11
Countess of Chester Hospital	Dr Gillian Brearton, Dr Salah Tueger	11
Kent and Canterbury Hospital	Dr Jindriska Lindsay	11
Kettering General Hospital	Dr Mark Kwan	11
Torbay Hospital, Torquay	Dr Heather Eve, Dr Deborah Turner	11
Doncaster Royal Infirmary	Dr Joe Joseph, Dr Youssef Sorour	10
George Eliot Hospital, Nuneaton	Dr Mekkali Narayanan	10
King's Mill Hospital, Sutton-in-Ashfield	Dr Tim Moorby, Dr Rowena Faulkner	10
The Christie, Manchester	Dr Samar Kulkarni, Dr Jim Cavet	10
Eastbourne Hospital, Conquest Hospital	Dr Sunil Gupta, Dr Simon Weston-Smith, Dr Satyajit Sahu	9
Poole Hospital	Dr Ram Jayaprakash, Dr Fergus Jacki	9
Royal Lancaster Infirmary	Dr David Howarth	9
Royal Oldham Hospital	Dr Hayley Greenfield	9
Russells Hall Hospital, Dudley	Dr Craig Taylor	9
Sunderland Royal Hospital	Dr Victoria Hervey, Dr Scott Marshall, Dr Simon Lyons	9
Arrowe Park, Wirral	Dr Ranjit Dasgupta, Dr Nauman Butt	8
Nevill Hall Hospital, Abergavenny	Dr Nilima Parry-Jones	8
Sandwell General Hospital, West Bromwich	Dr Farooq Wandroo	8
St Helens Hospital, Whiston Hospital	Dr Toby Nicholson	8
Salford Royal Hospital	Dr Simon Jowitt	7
Stoke Mandeville Hospital, Wycombe Hospital	Dr Robin Aitchison	7
Borders General Hospital, Melrose	Dr Jenny Buxton, Dr Srivnivasa Dasari, Dr John Tucker, Dr Ashok Okhandiar	6
Bradford Royal Infirmary	Dr Sam Ackroyd	6
Hereford County Hospital	Dr Lisa Robinson	6
Royal Liverpool Hospital	Dr Stephen Hawkins, Prof Patrick Chu	6
Addenbrookes Hospital, Cambridge	Dr Jenny Craig, Dr Charles Crawley	5

Darent Valley Hospital	Dr Tariq Shafi, Dr Anil Kamat	5
Royal Gwent Hospital, Newport	Dr Helen Jackson	5
Salisbury District Hospital	Dr Jonathan Cullis	5
Maidstone Hospital, Tunbridge Wells Hospital	Dr Don Gillett, Dr Lalita Banerjee	4
Queen's Hospital, Romford	Dr Sandra Hassan, Dr Biju Krishnan, Dr Jane Stevens	4
Royal Blackburn Hospital	Dr Malgorzata Rokicka, Dr Jagdish Adiyodi	4
Royal Bolton Hospital	Dr Chetan Patalappa, Dr Suzanne Roberts, Dr Mark Grey, Dr Claire Barnes	4
Royal Marsden Hospital, London	Dr Martin Kaiser, Prof Gareth Morgan	4
North Devon District Hospital, Barnstaple	Dr Paul Kerr, Dr Malcolm Hamilton	3
Pinderfields General Hospital Wakefield, Dewsbury & District Hospital, Pontefract Hospital	Dr John Ashcroft	3
Rotherham General Hospital	Dr Richard Went, Dr Helen Barker	3
Stepping Hill Hospital, Stockport	Dr Montaser Haj	3
Warwick Hospital	Dr Carolina Arbuthnot	3
Ysbyty Gwynedd, Bangor	Dr Sally Evans, Dr Melinda Hamilton, Dr David Edwards	3
Glan Clwyd Hospital, Rhyl	Dr Earnest Hartin, Dr Christina Hoyle	2
Harrogate District Hospital	Dr Claire Hall	2
Royal Alexandra Hospital, Paisley	Dr Alison McCaig, Dr Alison Sefcick	2
Wythenshawe Hospital, Manchester	Dr Simon Watt	2
Colchester General Hospital	Dr Michael Hamblin, Dr Sudhakaran Makkuni	1
Dorset County Hospital	Dr Dietman Hofer, Dr Akeel Moosa	1
York Hospital, Scarborough General Hospital	Dr Laura Munro, Dr Haz Sayala	1

Text A – CONSORT Checklist

CONSORT 2010 checklist of information to include when reporting a randomised trial*

Section/Topic	Item No	Checklist item	Reported on paragraph No
**Title and abstract**			
	1a	Identification as a randomised trial in the title	Title
	1b	Structured summary of trial design, methods, results, and conclusions (for specific guidance see CONSORT for abstracts)	Abstract
**Introduction**			
Background and objectives	2a	Scientific background and explanation of rationale	Introduction 1
	2b	Specific objectives or hypotheses	Introduction 1
**Methods**			
Trial design	3a	Description of trial design (such as parallel, factorial) including allocation ratio	Study design and participants 1
	3b	Important changes to methods after trial commencement (such as eligibility criteria), with reasons	Study design and participants 3
Participants	4a	Eligibility criteria for participants	Study design and participants 2
	4b	Settings and locations where the data were collected	Study design and participants 2
Interventions	5	The interventions for each group with sufficient details to allow replication, including how and when they were actually administered	Randomisation and treatment 2 &-3
Outcomes	6a	Completely defined pre-specified primary and secondary outcome measures, including how and when they were assessed	Endpoints and assessments 1 & 4
	6b	Any changes to trial outcomes after the trial commenced, with reasons	N/A
Sample size	7a	How sample size was determined	Statistical analysis 2
	7b	When applicable, explanation of any interim analyses and stopping guidelines	Statistical analysis 2
Randomisation:			
Sequence generation	8a	Method used to generate the random allocation sequence	Randomisation and treatment 1
	8b	Type of randomisation; details of any restriction (such as blocking and block size)	Randomisation and treatment 1

Allocation concealment mechanism	9	Mechanism used to implement the random allocation sequence (such as sequentially numbered containers), describing any steps taken to conceal the sequence until interventions were assigned	Randomisation and treatment 1
Implementation	10	Who generated the random allocation sequence, who enrolled participants, and who assigned participants to interventions	Study design and participants 1 and Randomisation and treatment 1
Blinding	11a	If done, who was blinded after assignment to interventions (for example, participants, care providers, those assessing outcomes) and how	Randomisation and treatment 1
	11b	If relevant, description of the similarity of interventions	N/A
Statistical methods	12a	Statistical methods used to compare groups for primary and secondary outcomes	Statistical analysis 1
	12b	Methods for additional analyses, such as subgroup analyses and adjusted analyses	Statistical analysis 1
**Results**			
Participant flow (a diagram is strongly recommended)	13a	For each group, the numbers of participants who were randomly assigned, received intended treatment, and were analysed for the primary outcome	Figure 1; Results 2
	13b	For each group, losses and exclusions after randomisation, together with reasons	Figure 1; Table 2
Recruitment	14a	Dates defining the periods of recruitment and follow-up	Results 1
	14b	Why the trial ended or was stopped	Study design and participants 1
Baseline data	15	A table showing baseline demographic and clinical characteristics for each group	Table 1
Numbers analysed	16	For each group, number of participants (denominator) included in each analysis and whether the analysis was by original assigned groups	Results 1
Outcomes and estimation	17a	For each primary and secondary outcome, results for each group, and the estimated effect size and its precision (such as 95% confidence interval)	Results 2
	17b	For binary outcomes, presentation of both absolute and relative effect sizes is recommended	Results 3 & 4
Ancillary analyses	18	Results of any other analyses performed, including subgroup analyses and adjusted analyses, distinguishing pre-specified from exploratory	Statistical analysis 1 and Results 2
Harms	19	All important harms or unintended effects in each group (for specific guidance see CONSORT for harms)	Table 3
**Discussion**			
Limitations	20	Trial limitations, addressing sources of potential bias, imprecision, and, if relevant, multiplicity of analyses	Discussion 5
Generalisability	21	Generalisability (external validity, applicability) of the trial findings	Discussion 1 & 2
Interpretation	22	Interpretation consistent with results, balancing benefits and harms, and considering other relevant evidence	Discussion 1 & 2

Other information

Registration	23	Registration number and name of trial registry	Study design and participants 1
Protocol	24	Where the full trial protocol can be accessed, if available	Supplementary appendix
Funding	25	Sources of funding and other support (such as supply of drugs), role of funders	Manuscript meta data

*We strongly recommend reading this statement in conjunction with the CONSORT 2010 Explanation and Elaboration for important clarifications on all the items. If relevant, we also recommend reading CONSORT extensions for cluster randomised trials, non-inferiority and equivalence trials, non-pharmacological treatments, herbal interventions, and pragmatic trials. Additional extensions are forthcoming: for those and for up to date references relevant to this checklist, see www.consort-statement.org.

Text B – Myeloma XI+ Protocol

Myeloma XI

Myeloma XI

Randomised comparisons, in myeloma patients of all ages, of thalidomide, lenalidomide, carfilzomib and bortezomib induction combinations, and of lenalidomide and combination lenalidomide vorinostat as maintenance

EudraCT Number: 2009-010956-93
Sponsor ID: HM09/8885
ISRCTN49407852

Version 9.0
2nd November 2017

Chief Investigator:
Professor Graham Jackson
Northern Centre for Cancer Care, Freeman Hospital,
Freeman Road, Newcastle-upon-Tyne, NE7 7DN
Tel: 0191 2139379
Email: graham.jackson@newcastle.ac.uk

[redacted]

[redacted]

CONTACTS

CHIEF INVESTIGATOR

Professor Graham Jackson, Northern Centre for Cancer Care, Freeman Hospital, Freeman Road, Newcastle-upon-Tyne, NE7 7DN. Tel: 0191 2139379, Fax: 0191 201 0154, Email: graham.jackson@newcastle.ac.uk

CO-INVESTIGATORS

Professor Faith Davies, Myeloma Institute for Research and Therapy, University of Arkansas for Medical Sciences Email: FEDavies@uams.edu.org

Professor Nigel Russell, Department of Haematology, Nottingham City Hospital, Clinical Sciences Building, Hucknall Rd, Nottingham, NG5 1PB. Tel: 0115 9691169 Ext. 55564, Fax: 0115 9627742. Email: nigel.russell@nottingham.ac.uk

IMMUNOLOGY

Professor Mark Drayson, Myeloma Clinical Trials Unit, University of Birmingham, PO Box 1894, Edgbaston, Birmingham, B15 2SZ, Tel: 0121 414 4074, Fax: 0121 414 3069, Email: m.t.drayson@bham.ac.uk

CELLULAR STUDIES

Dr Martin Kaiser, The Institute of Cancer Research, Centre for Myeloma Research, Division of Molecular Pathology, Brookes Lawley Building, 15 Cotswold Road, London, Surrey, SM2 5NG Tel: 020 87224265, Fax: 0208 722 4266, Email: myeloma.lab@icr.ac.uk / martin.kaiser@icr.ac.uk

Dr Roger Owen, HMDS, Level 3 Bexley Wing, St James's University Hospital, Leeds, LS9 7TF. Tel: 0113 206 7851, Fax: 0113 206 7883, Email: rgowen@hmds.org.uk

PHARMACY LIAISON

Ms Pauline Brookes, Clinical Trials Pharmacy, Nottingham University Hospitals NHS Trust, City Campus, Hucknall Road, Nottingham, NG5 IPB. Tel 0115 9691169 Ext 59415, Email: pauline.brookes@nuh.nhs.uk

CO-ORDINATING CENTRE
Clinical Trials Research Unit, Leeds Institute of Clinical Trials Research,
University of Leeds, Leeds, LS2 9JT
Main contact telephone: 0113 343 1479 (Data Management Team)
Email: ctru_myelomaxi@leeds.ac.uk

Ms Rachel Sigsworth	Data Manager	Tel<br>Fax<br>Email	0113 343 1479<br>0113 343 6427<br>r.sigsworth@leeds.ac.uk
Mrs Andrea Paterson	Data Manager	Tel<br>Fax<br>Email	0113 343 1479<br>0113 343 6427<br>a.paterson@leeds.ac.uk
Mrs Anna Waterhouse	CTRU Project Delivery Lead	Tel<br>Fax<br>Email	0113 343 7685<br>0113 343 6427<br>a.k.waterhouse@leeds.ac.uk
Dr Gwen Jacques	Senior Trial Coordinator	Tel<br>Fax<br>Email	0113 343 1159<br>0113 343 6427<br>g.jacques@leeds.ac.uk
Professor Walter Gregory	CTRU Scientific Lead	Tel<br>Fax<br>Email	0113 343 1489<br>0113 343 1471<br>w.m.gregory@leeds.ac.uk
Dr David Cairns	Supervising Trial Statistician	Tel<br>Fax<br>Email	0113 343 1712<br>0113 343 1471<br>d.a.cairns@leeds.ac.uk
Miss Alina Striha	Trial Statistician	Tel<br>Fax<br>Email	0113 343 9077<br>0113 343 1471<br>a.striha@leeds.ac.uk

Direct lines for 24-hour randomisation

0113 343 1469

(initial, VCD and maintenance randomisations for participants entering trial under PV6.0 and beyond)

0113 343 5029

(VCD and maintenance randomisations for participants who entered trial prior to PV6.0)

CONTENTS

STUDY SUMMARY **8**
GLOSSARY **11**
1 BACKGROUND AND RATIONALE **13**
1.1 Background 13
1.2 Thalidomide 13
1.3 Lenalidomide 14
1.4 Carfilzomib 15
1.5 Bortezomib 18
1.6 Vorinostat 19
1.7 Supportive care 21
1.7.1 Bisphosphonates 21
1.7.2 Thromboprophylaxis 21
1.8 Biological factors 22
1.8.1 FISH-based cytogenetics 22
1.8.2 Molecular monitoring 22
1.9 Summary and rationale for therapeutic study 23
1.9.1 Intensive treatment pathway 23
1.9.2 Non-intensive treatment pathway 23
1.9.3 Both treatment pathways 23
2 AIMS AND OBJECTIVES **25**
2.1 Therapeutic questions within the intensive pathway 25
2.2 Therapeutic questions within the non-intensive pathway 25
2.3 Therapeutic questions across both pathways 25
2.4 Sub-study objective 25
2.4.1 Evaluation of the effect of IMiDs on EBV lifecycle in plasma from multiple myeloma patients in the Myeloma XI trial 25
2.5 Further translational objectives 26
3 TRIAL DESIGN **27**
4 ELIGIBILITY **28**
4.1 Inclusion criteria for initial randomisation 28
4.2 Exclusion criteria for initial randomisation 28
4.3 Inclusion criteria for randomisation to bortezomib, cyclophosphamide and dexamethasone 29
4.4 Exclusion criteria for randomisation to bortezomib, cyclophosphamide and dexamethasone 29
4.5 Inclusion criteria for maintenance randomisation 29
4.6 Exclusion criteria for maintenance randomisation 30

5 RECRUITMENT AND CONSENT 31
5.1 Recruitment 31
5.2 Informed consent process 31
5.2.1 Loss of Capacity Following Informed Consent 32
6 BASELINE INVESTIGATIONS 34
7 RANDOMISATION PROCEDURES 35
7.1 Initial randomisation – induction chemotherapy 35
7.2 Bortezomib-cyclophosphamide-dexamethasone (VCD) randomisation 36
7.3 Maintenance randomisation 37
8 TRIAL MEDICINAL PRODUCT MANAGEMENT 38
8.1 Investigational Medicinal Products 38
8.1.1 IMP formulation and storage 39
8.1.2 IMP preparation 40
8.1.3 IMP labelling and handling 40
8.1.4 IMP administration 40
8.2 Non-Investigational Medicinal Products 41
9 STUDY TREATMENT 42
9.1 Intensive pathway treatment 42
9.1.1 Intensive pathway outline 42
9.1.2 Induction chemotherapy treatment 43
9.1.3 Consolidation chemotherapy treatment 45
9.1.4 Stem cell mobilisation and harvest 45
9.1.5 High-dose melphalan (HDM) regimen and autologous stem cell transplant 46
9.1.6 Maintenance 46
9.1.7 Relapsed participants 46
9.2 Non-Intensive pathway treatment 47
9.2.1 Non-intensive pathway outline 47
9.2.2 Induction chemotherapy treatment 48
9.2.3 Consolidation chemotherapy treatment 49
9.3 Maintenance (Intensive and Non-Intensive pathways) 49
9.3.1 Maintenance schedules 50
9.3.2 Prescribing and pregnancy testing 50
9.3.3 Dose reduction schedules for maintenance 51
9.3.4 Relapsed participants 51
9.4 Supportive measures 51
9.5 Contraindicated concomitant medication 53
9.6 Withdrawal of treatment 53
10 LABORATORY INVESTIGATIONS AND DATA COLLECTION 54
10.1 Baseline investigations 54
10.1.1 Local investigations at presentation 54
10.1.2 Central investigations at presentation 55
10.2 Follow-up investigations 55
10.2.1 Local follow-up 55
10.2.2 Central follow-up 56
10.2.3 Response and relapse assessment 56

10.2.4 Toxicity 56
10.2.5 Follow up for SPMs 56
10.2.6 Death 56
10.3 Definition of end of trial 56
11 PHARMACOVIGILANCE PROCEDURES 57
11.1 General definitions 57
11.1.1 Adverse events (AEs) 57
11.1.2 Serious Adverse Events (SAEs) 57
11.1.3 Adverse reactions (ARs) 57
11.1.4 Serious Adverse Reaction (SAR) 57
11.1.5 Suspected Unexpected Serious Adverse Reaction (SUSAR) 57
11.2 Operational definition and reporting adverse events and reactions 58
11.2.1 Recording and reporting thromboembolic events 58
11.2.2 Recording and reporting pregnancies/suspected pregnancies 58
11.3 Operational Definition – Serious Adverse Events (SAEs) 58
11.3.1 Events not classed as SAEs 58
11.3.2 Recording and reporting SAEs and SUSARs 59
11.4 Pregnancies or suspected pregnancies 60
11.5 Responsibilities 61
12 CRITERIA OF RESPONSE 62
13 ENDPOINTS 63
13.1 Primary 63
13.2 Secondary 63
13.3 Study Definitions 63
14 STATISTICAL CONSIDERATIONS 65
14.1 Sample size 65
14.1.1 Protocol version 2.0 - Protocol version 5.0 65
14.1.2 Protocol version 6.0 67
14.2 Planned recruitment rate 72
14.3 Analysis timelines 72
14.3.1 Induction and Consolidation therapy 75
14.3.2 Maintenance therapy 75
15 STATISTICAL ANALYSIS 77
15.1 General considerations 77
15.2 Formal interim analyses 77
15.3 Primary endpoint analyses 78
15.4 Secondary endpoint analyses 78
15.5 Subgroup Analyses 78
16 DATA MONITORING 79
16.1 Data monitoring and ethics committee 79
16.2 Data monitoring 79
16.3 Clinical governance issues 79

17 QUALITY ASSURANCE, SPONSORSHIP, ETHICAL CONSIDERATIONS, CONFIDENTIALITY and STATEMENT OF INDEMNITY 80
17.1 Quality assurance 80
17.2 Sponsorship 80
17.3 Ethical considerations 80
17.4 Confidentiality 80
17.5 Archiving 81
17.6 Statement of indemnity 81
18 STUDY ORGANISATIONAL STRUCTURE 82
18.1 Responsibilities 82
18.2 Operational Structure 82
19 PUBLICATION POLICY 83
20 KEY REFERENCES 84
21 APPENDICES 93
Appendix A – Definition of myeloma and related diseases 93
Appendix B – WHO grades of performance status 95
Appendix C – Definitions of response 96
Appendix D – National Cancer Institute Common Toxicity Criteria (NCIC) 99
Appendix E – Local investigations and sample collection for central investigation 100
Appendix F – Axial skeletal survey 104
Appendix G – Definition of a woman of childbearing potential 105
Appendix H – Dose modifications for induction regimens 106
Appendix I – Dose modifications for VCD 112
Appendix J – Suggested dose reduction schedule for lenalidomide maintenance and lenalidomide with vorinostat maintenace 114
Appendix K – Definition of high risk venous thrombosis 118
Appendix L – Central laboratory addresses 119

STUDY SUMMARY

The last ten years has seen the introduction of a number of effective new anti-myeloma agents into the clinical arena. These agents have been shown to be highly effective in the relapse setting and now are being introduced as treatment earlier in the disease course.

This study aims to address in the randomised setting some of the key questions concerning the use of thalidomide, bortezomib, carfilzomib, lenalidomide and vorinostat in the initial treatment of multiple myeloma patients.

Newly diagnosed patients of all ages with symptomatic myeloma requiring treatment are eligible.

For initial treatment, thalidomide in combination with cyclophosphamide and dexamethasone, the UK gold standard, will be compared with the newer combinations of lenalidomide, cyclophosphamide and dexamethasone with or without carfilzomib. This 4-drug combination (CCRD) has been added by a study amendment (Pv 6.0, 28th June 2013) and will be evaluated only in the younger fitter participant group who go on to receive transplantation. In this group the randomisation will be that 50% of participants will receive the new 4-drug combination while the other 50% will follow the original study randomisation to either CTD or RCD. The older pathway did not change in this amendment.

For participants randomised to CTD or RCD in the intensive arm, or CTD(a) or RCD(a) in the non-intensive arm with a sub-optimal response to initial therapy, the response to the proteasome inhibitor bortezomib will be assessed, as previous studies have demonstrated that it is able to induce responses and improve progression-free and overall survival in participants resistant to standard chemotherapy. Participants young and fit enough to tolerate an autologous transplant will then proceed to high dose melphalan with peripheral blood stem cell rescue. Older or less fit participants will go directly to a maintenance randomisation.

For participants who entered the trial prior to Pv 6.0, the value of lenalidomide and lenalidomide combined with vorinostat maintenance will then be assessed by randomising eligible participants to receive either lenalidomide, lenalidomide combined with vorinostat maintenance therapy, or close observation.

The value of lenalidomide maintenance versus close observation will be assessed for participants who enter the trial under Pv 6.0.

The primary end points of the study are overall and progression-free survival (OS and PFS). Secondary end points include response and toxicity.

A number of laboratory-based studies will also be performed in order to determine participant specific factors predicting overall and progression-free survival and response to treatment.

The study has undergone peer review and is supported by the NCRN and CTAAC.

INTENSIVE PATHWAY OUTLINE

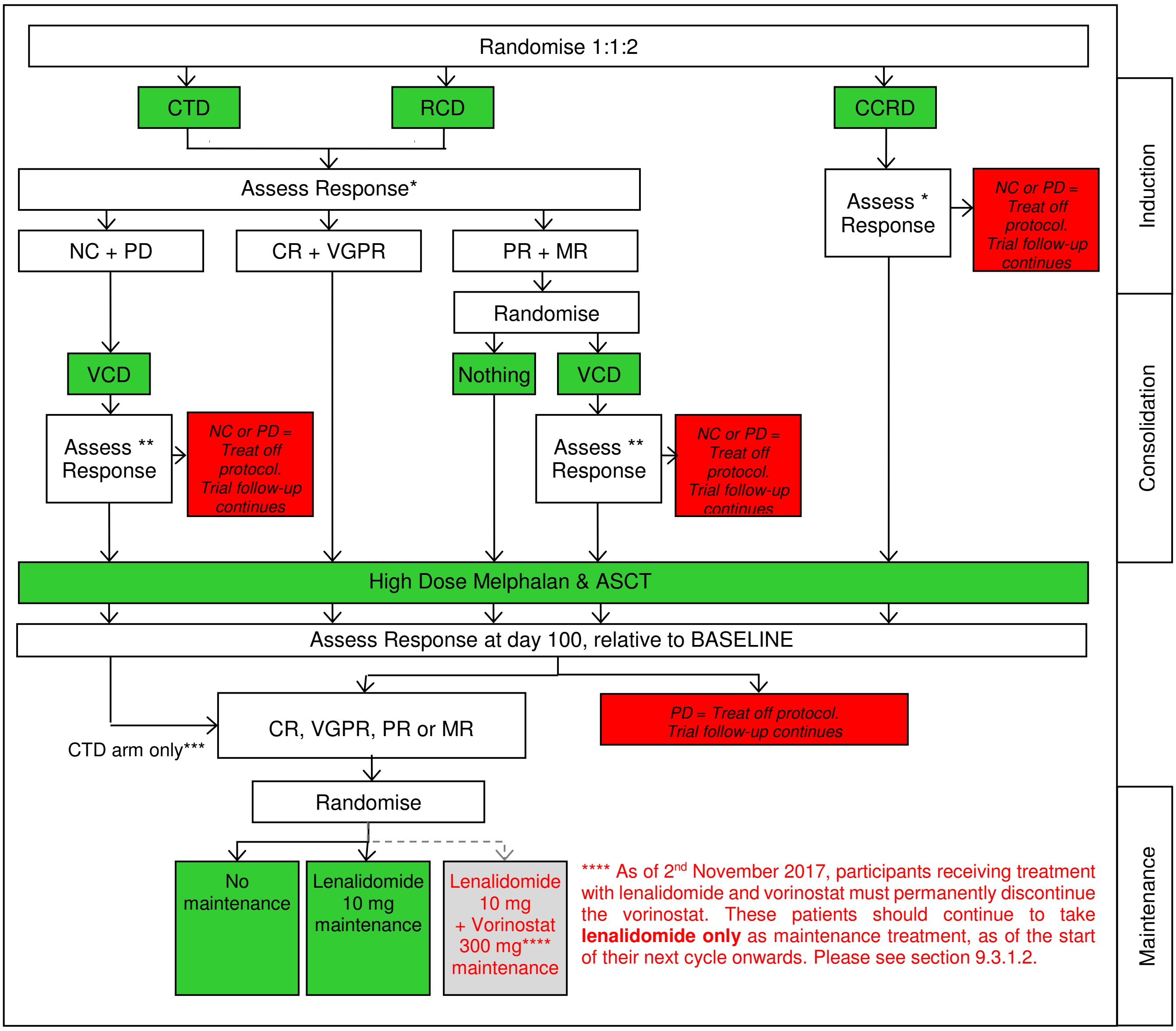

** In the absence of disease progression participants should receive a minimum of 4 cycles of induction chemotherapy (as long as they are responding) and should continue to maximum response or intolerance. Any participants receiving CTD or RCD showing NC after 4 cycles or progressive disease at any time during their induction chemotherapy should proceed to VCD. Participants receiving CCRD **will not** receive VCD.*

*** In the absence of disease progression participants should receive up to a maximum of 8 cycles of VCD and should continue to maximum response or intolerance.*

**** Participants entered into the RCD or CCRD arm and assessed as NC or PD at the end of induction are not eligible for the maintenance randomisation.*

***** Lenalidomide plus vorinostat maintenance is only available for those participants who were entered into the trial prior to PV6.0. NB: See Section 9.3.1.2 (page 50) for ongoing treatment details of participants randomised to the lenalidomide + vorinostat arm*

NON-INTENSIVE PATHWAY OUTLINE

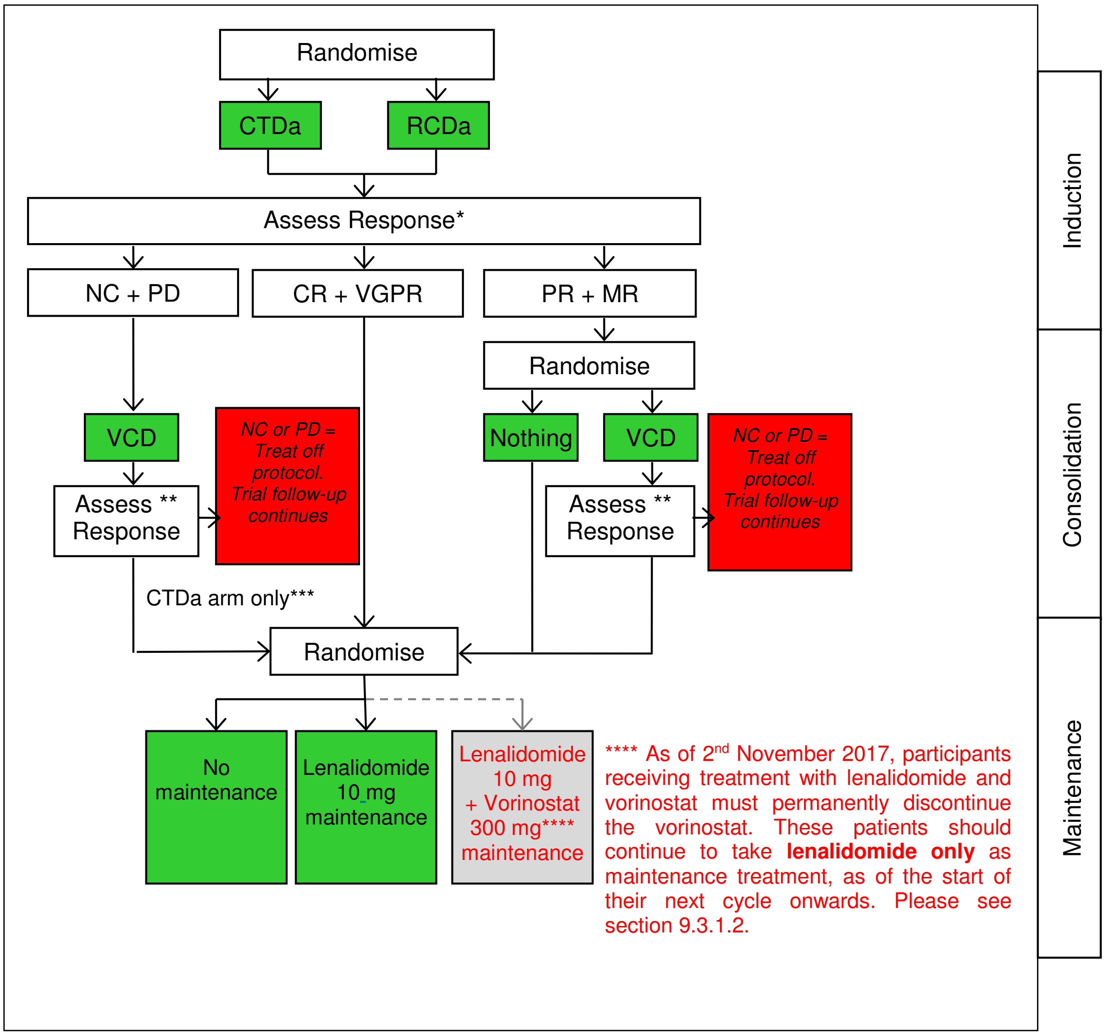

** In the absence of disease progression participants should receive a minimum of 6 cycles of induction chemotherapy (as long as they are responding) and should continue to maximum response or intolerance. Participants showing NC after 4 cycles or progressive disease at any time during their induction chemotherapy should proceed to VCD*

*** In the absence of disease progression participants should receive up to a maximum of 8 cycles of VCD and should continue to maximum response or intolerance.*

****Participants entered into the RCDa arm and assessed as NC or PD at the end of RCDa induction are not eligible for the maintenance randomisation*

***** Lenalidomide plus vorinostat maintenance is only available for those participants who were entered into the trial prior to PV6.0. NB: See Section 9.3.1.2 (page 50) for ongoing treatment details of participants randomised to the lenalidomide + vorinostat arm*

GLOSSARY

ABCM	Adriamycin, BCNU, cyclophosphamide, melphalan
AE	Adverse event
AR	Adverse reaction
ASCT	Autologous stem cell transplant
BCSH	British Committee for Standards in Haematology
BJP	Bence-Jones protein
CCRD	Carfilzomib, cyclophosphamide, lenalidomide and dexamethasone
CRd	Cyclophosphamide, lenalidomide and low dose dexamethasone
CI	Chief investigator
CR	Complete response
CRF	Case report form
CTA	Clinical Trial Authorisation
CTCAE	Common terminology criteria for adverse events
CTD	Cyclophosphamide, thalidomide, dexamethasone
CTDa	Attenuated cyclophosphamide, thalidomide, dexamethasone
CTRU	Clinical Trials Research Unit
CVAD	Cyclophosphamide, vincristine, doxorubicin and dexamethasone
CVAMP	Cyclophosphamide, vincristine, doxorubicin and methylprednisolone
DMEC	Data monitoring and ethics committee
DNA	Deoxyribonucleic acid
DOR	Duration of response
DVT	Deep vein thrombosis
FBC	Full blood count
FISH	Fluorescent in situ hybridisation
GCP	Good clinical practice
G-CSF	Granulocyte colony stimulating factor
HDT	High-dose therapy
HDM	High-dose melphalan
IB	Investigator's brochure
ICMJE	International Committee of Medical Journal Editors
IFM	Intergroupe Françophone du Myelome
IMiD	Immunomodulatory drugs
IMP	Investigational medicinal product
IV	Intravenous
LDH	Lactate dehydrogenase
LMWH	Low molecular weight heparin
LOH	Loss of heterozygosity
MHRA	Medicines and Healthcare Products Regulatory Authority
MP	Melphalan plus prednisolone
MPR	Melphalan, prednisolone, lenalidomide
MR	Minimal response
MRC	Medical Research Council
MRD	Minimal residual disease
NC	No change
NCI	National Cancer Institute
NCRI	National Cancer Research Institute

NDA	New drug application
NSAIDs	Non-steroidal anti-inflammatory drugs
ORR	Overall response rate
OS	Overall survival
PCR	Polymerase chain reaction
PD	Progressive disease
PFS	Progression-free survival
PI	Principal investigator
PO	*Per os* – Oral
PR	Partial response
PV	Protocol version
R&D	Research and development
RCD	Lenalidomide, cyclophosphamide, dexamethasone
RCDa	Attenuated lenalidomide, cyclophosphamide, dexamethasone
REC	Research ethics committee
RSA	Research sponsorship agreement
RZ	Revlimid™, Zolinza™
SAE	Serious adverse event
SAR	Serious adverse reaction
SD	Stable disease
SNP	Single nucleotide polymorphism
SOP	Standard operating procedure
SmPC	Summary of product characteristics
SPM	Second primary malignancy
SUSAR	Suspected unexpected serious adverse reaction
TLS	Tumour lysis syndrome
TMG	Trial management group
TSC	Trial steering committee
UKMF	UK Myeloma Forum
VAD	Vincristine, doxorubicin and dexamethasone
VCD	Bortezomib, cyclophosphamide, dexamethasone
VGPR	Very good partial response
VTE	Venous thromboembolism
WCBP	Woman of child bearing potential
WHO	World Health Organization

1. BACKGROUND AND RATIONALE

1.1 BACKGROUND

Myeloma is a malignant disorder of plasma cells which is characterised by an excess of abnormal plasma cells, lytic bone lesions and paraproteins in the serum and urine. It is frequently associated with painful bone lesions, fractures, myelosuppression and renal failure. The underlying pathogenesis of myeloma is not fully understood, but recurrent chromosomal abnormalities are frequent. In particular, translocations into chromosome 14q32 are common and thought to be mediated via abnormal immunoglobulin class switch recombination. Aneuploidy is another common feature, the cause of which is unknown. It is a relatively common disease with an increasing incidence with age, the majority of cases occurring over the age of sixty. Cases occurring in the childbearing age group are rare. It is an incurable condition which, in the absence of treatment, has a very poor prognosis. With modern treatments the median overall survival is approximately 4-5 years. As well as developing effective chemotherapy, some of the most important clinical aspects of disease management relate to ameliorating bone disease and renal failure.

Approaches to the treatment of myeloma have developed over the last 30 years. In early studies from the Medical Research Council (MRC), the equivalence of cyclophosphamide and melphalan was identified; however, oral treatment with melphalan became the world standard treatment. In the 1980s the value of combination chemotherapy was investigated and in the MRC Myeloma V trial, melphalan alone was compared with ABCM (Adriamycin, BCNU, cyclophosphamide and melphalan). In this study there were significant differences in the achievement of plateau (49% vs 61%) and in median overall survival (24 months vs 32 months), indicating that ABCM was more effective than melphalan. Despite this, overviews of published trials and of individual participant data from trials did not show a significant advantage for other combinations, in comparison with the global standard of melphalan plus prednisolone (MP). Thus, until recently, melphalan remained the world standard against which new developments were compared.

The first randomised trial to compare standard chemotherapy with high-dose therapy (HDT) with stem cell support was carried out by the Intergroupe Françophone du Myelome (IFM). In an 'intention-to-treat' analysis there was a significant advantage for participants in the intensive arm both in terms of response rate, response duration and survival, with a median overall survival of 56 months compared with 44 months in the standard arm. The MRC Myeloma VII trial randomised 400 participants, addressing the same question, comparing ABCM with a more intensive regimen, C-VAMP (cyclophosphamide, vincristine, adriamycin and methylprednisolone) followed by high-dose melphalan 200 mg/m^2. Response rates and response durations were improved in the HDT arm and there was a significant improvement in overall survival, with a median survival of 54 months compared with 42 months. Thus, we were left with a situation where MP was the standard treatment for elderly less fit patients, and VAD (vincristine, adriamycin and dexamethasone) -type treatment followed by HDT was the standard for younger fitter patients.

1.2 THALIDOMIDE

Recently a number of new effective treatment modalities for myeloma have been developed and introduced into the clinic. *In vitro* studies suggest thalidomide not only causes apoptosis

of myeloma cells, but also has an anti-angiogenic effect and enhances tumour cell immuno-surveillance. In initial clinical trials on groups of heavily pre-treated participants at relapse, a group unlikely to respond to conventional chemotherapy, response rates of 30-40% were seen. *In vitro*, the combination of dexamethasone with thalidomide potentates the anti-myeloma effect of thalidomide and *in vivo*, the combination seems to be particularly effective, increasing the number of responses. Side effects were noted in these studies, which can impair the ability to deliver thalidomide, including neurotoxicity (≤30%) and deep vein thrombosis (DVT) (≤15%).

In younger fitter patients, combinations of thalidomide and dexamethasone in the presenting setting have been explored and have been shown to be effective and not to impair stem cell mobilisation. In presenting patients, data suggests responses are greater, occur more rapidly and are associated with fewer infections than with VAD. Worldwide it is now widely accepted that the VAD regimen will no longer be the main induction regimen for patients going for transplantation and that it will be replaced by a thalidomide-containing regimen. In the UK, this will be cyclophosphamide, thalidomide and dexamethasone (CTD), a regimen investigated in the Myeloma IX study. Preliminary results from this study comparing CTD with CVAD (cyclophosphamide, vincristine, adriamycin and dexamethasone), demonstrate increased response rates post induction chemotherapy with CTD (ORR 91.4%, CR 20.8%) compared to those with CVAD (ORR 81.6%, CR 14.0%), and 3 months following autologous transplant an ORR of 98.0% and a CR of 65.4% in the CTD arm compared to ORR 93.4% and CR 48.0% in the CVAD arm.

As melphalan was considered the standard treatment for older less fit patients, it was natural that it should be combined with thalidomide. The combination of MPT (melphalan, prednisolone and thalidomide) is associated with both increased responses (15% CR) and survival in three randomised studies. Thus it is likely to be taken up widely as the standard approach for patients not destined for transplantation. This regimen is relatively toxic and difficult to deliver. In the UK, CTDa (CTD with a reduced dose of dexamethasone and lower starting dose of thalidomide) was compared in the older, less fit population in Myeloma IX to MP. Preliminary results demonstrate it induces significantly higher response rates (ORR 83.1%, CR 21.3%) compared to those with MP (ORR 46.1%, CR 4.1%).

Importantly, CTD does not damage haemopoietic stem cells. Thus, CTD followed by HDT in younger patients, and CTDa in the elderly are the standard comparators against which new treatments are to be assessed.

In our previous study, Myeloma IX, maintenance thalidomide was shown to deliver a better PFS with an improved survival in cases treated with effective relapse schedules. However, the toxicity of thalidomide is such that participants only received a median of 7 months on therapy and there was significant impairment of quality of life and, therefore, the standard for comparison remains no ongoing maintenance.

1.3 LENALIDOMIDE

Lenalidomide (Revlimid™) is a thalidomide derivative, also available as an oral preparation, which is more potent in *in vitro* assays with a different adverse effect profile than thalidomide. It is administrated daily for 21 days of a 28-day cycle, usually with 2-3 pulses of dexamethasone per treatment course. It has been shown to be effective in the treatment of myeloma at relapse in two large phase III studies in Europe and the US using the same protocol (lenalidomide/dexamethasone vs dexamethasone). These trials showed identical

results confirming the superiority of the combination lenalidomide plus dexamethasone to dexamethasone alone both in terms of response (CR 15% vs 2%, ORR 60% vs 22%) and survival (PFS 11.1 month vs 4.7 month, OS 29.6 month vs 20.2 month). A major potential benefit of lenalidomide is the absence of associated neurotoxicity or sedation, making it more tolerable; however, there is a significant rate of myelosuppression (20%) seen with this drug, which is not seen with thalidomide. The rates of DVT are the same as those seen with thalidomide.

From a number of phase II clinical trials the combination of lenalidomide with dexamethasone has also been shown to induce good responses in newly diagnosed participants, with 91% of participants achieving a partial response (PR) or greater. Importantly stem cells can be mobilised following lenalidomide therapy, although a recent report suggests this should be done within 6 months of therapy and using a cyclophosphamide based mobilisation regimen.

Preliminary results of a phase III study comparing lenalidomide plus high-dose dexamethasone (40 mg day 1-4, 9-12 and 17-20 every 28 days) to lenalidomide plus low-dose dexamethasone (40 mg day 1, 8, 15 and 22 every 28 days) suggest an increase in toxicity and a poorer 1 year survival in the high-dose dexamethasone arm (87% vs 96%), suggesting some care needs to be given to the dose of dexamethasone in the older participant group.

We have carried out both a pilot study and dose-finding study of the combination lenalidomide, cyclophosphamide and dexamethasone (RCD) in relapsed participants and found it well tolerated and highly effective, giving better responses than would be expected with RD alone and potentially having the benefit of the stable response phase previously noted with single agent cyclophosphamide. Other groups have evaluated a similar combination MP plus lenalidomide (MPR) and found it effective, but with a worse side-effect profile, particularly damaging stem cells, therefore, is inappropriate for use prior to Autologous Stem Cell Transplant (ASCT).

Three studies have been presented in abstract suggesting an important clinical benefit for the use of maintenance lenalidomide in newly diagnosed myeloma in both younger and older participants. The MM015 study, in transplant ineligible participants, showed that continuing lenalidomide after induction with MPR significantly prolonged PFS. The IFM 2002 study using a dose of 10 mg of lenalidomide as maintenance after HDT with autologous stem cell rescue dramatically improved PFS, with some suggestion of a benefit for OS. The third study has been carried out by the CALGB and had a similar design to the IFM study and showed an almost identical result. Thus, while the data for maintenance lenalidomide suggest that there is a clear and significant improvement in PFS, there remains some uncertainty around its impact on OS. The crucial question to answer, going forward, is whether the results seen with lenalidomide as a single agent for maintenance can be enhanced further by the use of a combination regimen.

1.4 CARFILZOMIB

Carfilzomib (Kyprolis®, also known as PR-171) is an α-keto-epoxy tetrapeptide inhibitor specific for the chymotrypsin-like active site of the 20S proteasome. Carfilzomib is structurally and mechanistically distinct from the dipeptide boronic acid proteasome inhibitor, bortezomib (Velcade®). In addition, when measured against a broad panel of proteases

including metallo, aspartyl, and serine proteases, carfilzomib demonstrated less reactivity against non-proteasomal proteases when compared to bortezomib.

A Phase I clinical trial, PX-171-002, testing carfilzomib in patients with relapsed/refractory haematologic malignancies, is now complete. During the dose escalation portion of the trial, 36 participants received carfilzomib on Days 1, 2, 8, 9, 15, and 16 of a 28-day cycle. Patients with Multiple Myeloma (MM), Non-Hodgkin's Lymphoma (NHL), Waldenström's Macroglobulinemia, and Hodgkin's Lymphoma (HL) were enrolled on the study.

No dose limiting toxicities (DLTs) were observed in the initial seven cohorts (doses ranged from 1.2 to 15 mg/m^2) of three participants each. At the 20 mg/m^2 dose level, one of eight patients had a Grade 3 renal failure at Cycle 1, Day 2 which was considered possibly related to study drug and lasted for six days. The participants continued on study for the remainder of Cycle 1 before having disease progression. At the 27 mg/m^2 dose level, one of six participants experienced a DLT during Cycle 1, consisting of severe hypoxia with pulmonary infiltrates following Day 2 of dosing.

In participants where the 27 mg/m^2 dose was efficacious, a "first dose effect" was seen that included a constellation of findings that appeared to be the clinical sequelae of rapid tumour lysis syndrome (TLS) and/or cytokine release. This effect was notable for fever, chills, and/or rigors occurring during the evening following the first day of infusion. On the second day, three of five participants with multiple myeloma experienced an increase in creatinine to Grade 2 (including the participants with the DLT). This elevation was rapidly reversible and all three participants were re-challenged with carfilzomib without recurrence of the events. Interestingly, all three participants had a rapid decline in serum and/or urine M-protein levels; two participants achieved a partial response (PR) and the third participant achieved a minimal response (MR). There were no consistent changes in potassium, calcium, phosphorous, or uric acid levels although some increases in LDH and other markers of tumour lysis were noted. Because of the possible TLS and reversible creatinine elevations, hydration and very-low dose dexamethasone prophylaxis were instituted in subsequent studies and have essentially eliminated clinically significant TLS/creatinine elevations and the other "first-dose" effects.

Haematologic toxicities were primarily mild or moderate. The thrombocytopenia reported with carfilzomib is cyclical and similar to that reported with bortezomib. The cause and kinetics of the thrombocytopenia following treatment are different from those of standard cytotoxic agents. To maximise the likely benefit of carfilzomib, participants with thrombocytopenia should be supported as clinically indicated rather than having treatment reduced due to thrombocytopenia.

The response rate in the phase 2 study PX-171-003-A0 was 18% PR, 7% MR and 41% SD in these patients that entered the study with progressive disease and were refractory to their most recent therapy, often including bortezomib and/or an immunomodulatory drug (usually lenalidomide). The median time to progression on the PX-171-003-A0 study was 5.1 months with a duration of response of 7.4 months (mean follow up of 7.6 months).

A "stepped up" dosing schedule, referred to as 20/27 mg/m^2, has subsequently been incorporated into the PX-171-003 study (referred to as PX-171-003-A1) in order to maximize the clinical benefit of carfilzomib. Participants receive 20 mg/m^2 for the first cycle and 27 mg/m^2 thereafter. An independent Safety Oversight Group (SOG) evaluated the safety data from the 40 of 250 participants to be enrolled on the 20/27 mg/m^2 schedule and agreed that

the trial should proceed without modification. The study completed enrolment of 266 participants by the end of 2009 and formed the basis for an accelerated approval NDA filing which was granted for single agent carfilzomib in July 2012. Of the 257 response-evaluable patients, the overall response rate was 23.7% by IRC assessment and the clinical benefit rate was 37.0%. The most common treatment-emergent AEs were fatigue (49%) and anaemia (46%), of which at G3/4 were thrombocytopenia (29%) and anemia (24%). The most common AEs of any grade possibly related to carfilzomib were fatigue (37%) and nausea (34%). One case of tumour lysis syndrome was reported, but this was no considered to be related to carfilzomib. The other most common adverse events were similar to the A0 portion of the study. Treatment-emergent peripheral neuropathy remains low on this portion of the study with 12% Grade 1/2 and three (1.1 %) Grade 3/4 events. In addition, anaemia rates in the PX-171-003-A1 (higher dose) were lower than those reported in the PX-171-003–A0 portion of the study, possibly indicating that the higher dose of carfilzomib is achieving better clearing of neoplastic cells in the bone marrow allowing superior normal marrow reconstitution.

PX-171-006 is an ongoing Phase 1b study in patients with relapsed multiple myeloma in which carfilzomib is administered in combination with lenalidomide (Revlimid®) and dexamethasone. "low-dose" dexamethasone 40 mg/day is given on days 1, 8, 15, and 22 in all cases. Carfilzomib is administered iv on days 1, 2, 7, 8, 15, and 16; lenalidomide is administered po on days 1 through 21. Enrolment has closed in this study, and no MTD was reached. The maximum per protocol doses of carfilzomib (27 mg/m^2) with lenalidomide 25 mg and low dose dexamethasone (CRd) are being used. After 8 participants tolerated these doses well, an additional 44 participants were enrolled in an "expansion" cohort at this level, and this regimen is being taken into Phase III in study PX-171-009.

To date, 40 participants were treated in cohorts 1-6 and 44 in the cohort 6 expansion. All 40 participants in cohorts 1-6 were included in the safety analysis and were evaluated for response. Participants were heavily pre-treated; 72% received prior bortezomib, thalidomide and vorinostat (BTZ) and 87.5% received prior lenalidomide or thalidomide. 47% of participants were refractory to their last therapy (typically lenalidomide and high dose dexamethasone; >84% of participants had a history of neuropathy with 67% BTZ- or thalidomide-related. Of the 28 participants who had discontinued treatment before completing full protocol treatment, 19 discontinued to due to progressive disease, 4 due to an adverse event, 2 withdrew consent and 3 discontinued for other reasons. The adverse events that led to the 4 patients discontinuing treatment were not considered to be related to carfilzomib. The most common haematological AEs ≥G3 were thrombocytopenia [n=13], anaemia [n=8], and neutropenia [n=17]. Only 4 patients experienced neuropathy, and all of who had a history of this. An evaluation of 27/32 participants in cohorts 1–5 revealed that 4 participants had drug-related SAEs as follows: transient G3 sinus bradycardia, G3 upper respiratory tract infection, febrile neutropenia, and G3 diarrhoea with G3 urinary infection. Overall response rate and clinical benefit response for the 40 participants are 62.5% and 75%, respectively. Efficacy data is shown in the table below. No deaths attributed to study treatment have been observed.

CRd: Cohorts 1–6
(Carfilzomib: 15 to 20 mg/m^2; Lenalidomide: 10 to 25 mg) (n=40)

Response	Number of participants	%

Stringent complete response	1	2.5
Very good partial response	13	32.5
Partial response	11	27.5
Minimal response	5	12.5
Stable disease	4	10.0
Progressive disease	2	5.0
Not evaluable	4	10.0

Together, these results suggest that carfilzomib, lenalidomide, and low-dose dexamethasone (CRd) in combination are active and well tolerated and that there are no significant overlapping toxicities (in the dose ranges tested). Importantly, lenalidomide-associated neutropenia and thrombocytopenia do not appear to be exacerbated by concurrent treatment with carfilzomib, even up to 27mg/m^2, suggesting that carfilzomib will combine well with other anti-cancer agents.

Preliminary data suggest that carfilzomib as a single agent can produce substantial response rates in myeloma patients across a variety of dosing cohorts. Responses were seen over a wide therapeutic window, from 15 to 27 mg/m^2. Maximum proteasome inhibition was seen at doses 11 mg/m^2 and higher in whole blood samples taken 1 hour after the first dose. Carfilzomib has been shown to be rapidly cleared from plasma with an elimination half-life of < 60 minutes at the 20 mg/m^2 dose.

Carfilzomib has been used in combination with cyclophosphamide, thalidomide and dexamethasone. The combination was well tolerated but neuropathy was seen and ascribed to the use of thalidomide (AK Stewart, Mayo Clinic, Scottsdale, AZ - personal communication). Using the doses proposed and the combination with lenalidomide we do not expect excess toxicity for the participants receiving the new 4-drug combination.

Only one DLT (grade 4 neutropenia on day 8 of cycle 1) was recorded in the study, and this was considered to be related to lenalidomide. The studies are ongoing and data is now available on the use of 36 mg/m^2 and 56 mg/m^2. Given the safety profile is similar to the 20/27 mg/m^2 studies and the potential for increased efficacy, we have chosen a dose of 36 mg/m^2 for this study.

1.5 BORTEZOMIB

Bortezomib (Velcade™) is a boron-containing molecule which reversibly inhibits the proteosome, an intracellular organelle which is central to the breakdown of ubiquinated proteins and consequently for normal cellular homeostasis. Proteosome inhibition with bortezomib can induce apoptosis in myeloma cell lines, particularly those resistant to conventional chemotherapy, via the simultaneous accumulation of contradictory cell cycle regulatory signals. It also dysregulates intracellular calcium metabolism, resulting in caspase activation and cell death. Bortezomib decreases the adhesion of the myeloma plasma cell to stromal cells which increases sensitivity to apoptosis, as well as interrupting pro-survival paracrine and autocrine cytokine loops in the bone marrow microenvironment mediated by IL6, IGF1, VEGF and TNFα.

It is administered intravenously in the outpatient setting on days 1, 4, 8 and 11 of a 21 day cycle. Following injection, maximum proteosome inhibition is observed within the first hour (80% inhibition), followed by partial recovery of proteosome activity over the next 6 to 24 hours to within 50% of the pre-treatment activity. Using the standard schedule, 10%-30%

proteosome inhibition is observed at the next scheduled dosing. This approach of using intermittent injections with a 1 week treatment holiday allows cells to recover proteosome activity and prevents excessive side effects. It is therefore important for the clinical use of bortezomib that its dose interval should not be brought closer together than 72 hours and the week off treatment is observed. Bortezomib does not cross the blood brain barrier. It is metabolised by the cytochrome P450 enzyme system in the liver, which de-boronates the molecule and removes it from the body, and only a small proportion is removed by the kidneys.

Phase II clinical trials demonstrate it is effective for the treatment of relapsed refractory myeloma, with overall response rates (CR, PR, and MR) of approximately 35%, with 10% CR or near CR. The response rate increased to 50% with the addition of dexamethasone (20 mg on days 1, 2, days 4, 5, days 8, 9 and days 11, 12). A randomised phase III trial comparing bortezomib to dexamethasone showed superiority in progression-free and overall survival. The response rate (CR and PR) was 38% in the bortezomib arm compared to 18% in the dexamethasone arm which translated into a 22% difference in overall survival at 12 months. In a sub-analysis, it was noted that response was independent of the number of previous lines of treatment, and type of previous treatment, confirming *in-vitro* data that bortezomib works via a different mechanism and overcomes resistance to other treatments.

Studies are ongoing looking at the use of bortezomib as first-line therapy, but encouraging response rates have been seen when used in combination with dexamethasone, cyclophosphamide and dexamethasone, melphalan and prednisolone, or adriamycin and dexamethasone. Importantly there does not seem to be any impairment in the capacity to harvest stem cells. Proteosome inhibition results in a different range of side-effects compared to that seen with classical chemotherapy, including peripheral neuropathy, autonomic neuropathy and thrombocytopenia. Practitioners need to be aware of this spectrum of side-effects in order to ensure its safe use. While the range of side-effects of bortezomib is wide, the majority are readily manageable; however because it is delivered in the outpatient setting, it is important to put in place a means of assessing and managing these effects.

1.6 VORINOSTAT

Histone deacetylases (HDAC) are enzymes that catalyze the removal of cacetyl groups from the lysine residues of proteins, including histones and transcription factors. In some cancer cells, there is an overexpression of HDACs, or an aberrant recruitment of HDACs to oncogenic transcription factors causing hypoacetylation of core nucleosomal histones which is associated with a condensed chromatin structure and repression of gene transcription. Inhibition of HDAC activity allows for the accumulation of acetyl groups on the histone lysine residues resulting in an open chromatin structure and transcriptional activation. HDAC inhibitors can induce tumour cell growth arrest, differentiation, or apoptosis *in vitro* and inhibit tumour growth in animals.

Vorinostat inhibits the enzymatic activity of histone deacetylases HDAC1, HDAC2 and HDAC3 (Class I) and HDAC6 (Class II) at nanomolar concentrations (IC50<86 nM). *In vitro*, vorinostat causes the accumulation of acetylated histones and induces cell cycle arrest and/or apoptosis of some transformed cells. The mechanism of the antineoplastic effect of vorinostat has not been fully characterised. Many other HDAC inhibitors have also shown synergistic or additive anti-myeloma activity when combined with other anticancer agents *in vitro*. Several of these HDAC inhibitors are in different phases of clinical development.

Vorinostat has been investigated as a single agent in patients with haematological malignancies, cutaneous T-cell lymphoma (CTCL) and various solid tumours. The types of adverse experiences observed in clinical trials of vorinostat were those usually associated with chemotherapy. The most common drug-related adverse experiences in patients treated with vorinostat could be classified into 4 symptom complexes: gastrointestinal symptoms (diarrhoea, nausea, anorexia, weight decrease, vomiting, and constipation), constitutional symptoms (fatigue, chills), haematologic abnormalities (thrombocytopenia, anaemia) and taste disorders (dysgeusia, dry mouth). Most of the adverse experiences were manageable. In fact, most of the very common adverse experiences were reversible and could be managed using conventional supportive care for chemotherapy. On the whole, treatment with oral vorinostat was well tolerated. A total of 146 of the 305 participants (47.9%) experienced one or more adverse experiences that were Grade 3 or greater in severity, and were at least possibly related to vorinostat. The occurrences of specific Grade 3 or greater adverse experiences that were observed in 5% or more of the participants were as follows: fatigue (13.7%), thrombocytopenia (11.2%) and decreased platelet count (4.3%), nausea (5.6%), and anaemia (2.3%) and decreased haemoglobin (3.3%). The occurrence of other Grade 3 or greater events of interest included anorexia (4.9%), diarrhoea (4.6%) and hyperglycemia (3.3%). Pulmonary embolism and deep vein thrombosis have been reported. Across all populations 3.1% participants experienced deep vein thrombosis, and 2.3% participants experienced pulmonary embolism. QT prolongation has been observed, but none had QTc intervals >500 msec. Several studies of standard dose vorinostat (400 mg) in combination with novel agents have been conducted in myeloma and confirmed that standard dose vorinostat (400 mg) was well tolerated.

Vorinostat (Zolinza™) was approved by the U.S. Food and Drug Administration (FDA) on 6-Oct-2006 for the treatment of cutaneous manifestations in patients with cutaneous T-cell lymphoma (CTCL) who have progressive, persistent or recurrent disease on or following two systemic therapies. Along with several other oncologic indications, vorinostat is being tested in multiple myeloma both as monotherapy as well as combination therapy. Mitsiades *et al.* showed that multiple myeloma cells are sensitive to vorinostat. Vorinostat directly targets the transcriptional machinery of tumour cells. By inducing up-regulation of several pro-apoptotic genes and down-regulation of anti-apoptotic genes, vorinostat potently induces apoptosis of multiple myeloma cells. Furthermore, the multiple myeloma cells are irreversibly committed to cell death after a few hours of incubation with vorinostat. Vorinostat is also associated with early changes in gene expression profile, including suppression of genes mediating cytokine-driven proliferation and survival, drug-resistance, cell cycle control, DNA synthesis/repair, and proteasome function.

Vorinostat also enhances the anti-myeloma activity of other pro-apoptotic agents, including dexamethasone, IMiD and cytotoxic chemotherapy. The vorinostat-induced senitisation to dexamethasone and IMiD-induced apoptosis was confirmed in primary tumour samples of myeloma patients resistant to conventional therapies (including dexamethasone-or thalidomide-based regimens). Preliminary preclinical studies results indicate that vorinostat synergizes with the anti-myeloma activity of both lenalidomide and dexamethasone. Like lenalidomide, vorinostat has been reported to diminish the production of VEGF as well as IL-6 and appears to mediate anti-angiogenic effects. In addition, both agents can switch on tumour suppressor genes inactivation, which leads to disease progression, and so may improve outcome. In addition recent evidence that changes epigenetic plasticity at a stem cell level may mediate progression from stable residual disease or plateau states. Modifying this epigenetic state in an ongoing fashion with lenalidomide and vorinostat may prolong the

duration of stable disease phases. These findings shed light on the complex molecular sequelae of vorinostat exposure and provide a preclinical rationale for the clinical evaluation of vorinostat in combination with lenalidomide in multiple myeloma.

In a Phase I clinical study (Protocol 074) of vorinostat in combination with lenalidomide +/- dexamethasone in relapsed or refractory multiple myeloma, the preliminary safety data indicated that the combination was generally well tolerated. The Maximum Tolerated Dose (MTD) was not observed due to non-occurrence of ≥ 2 DLT / 6 participantss in any of the 5 dosing cohorts. Participants in Cohort 5 tolerated the highest dose study level in the study, i.e., vorinostat 400 mg orally days 1-7 and 15-21, lenalidomide 25 mg orally days 1-21 and dexamethasone 40 mg orally once weekly; this was considered the “Maximum Administered Dose”. Of 28 participants evaluable for efficacy, 86% experienced a clinical benefit with an overall response rate of 46%. Of these 28 participants, 2 participants achieved a complete response, 11 participants achieved a partial response, 5 participants achieved a minor response, 6 participants had stable disease and 4 participants experienced progression of disease.

1.7 SUPPORTIVE CARE

1.7.1 Bisphosphonates

The importance of bisphosphonate therapy for all patients with multiple myeloma is now well established. Previous studies have shown that clodronate, initiated at the start of induction treatment and continued long-term, reduced the incidence of hypercalcaemia and fractures, some of the major sources of morbidity in myeloma. Clodronate is an orally-available second generation bisphosphonate. Third generation, nitrogen-containing aminobisphosphonates, exemplified by pamidronate and zoledronic acid, are more potent *in vitro* and have also been extensively investigated in clinical practice. In addition to the inhibitory effects on osteoclasts, there is evidence that third generation bisphosphonates have direct anti-myeloma effects and may potentially increase survival. In the previous study, Myeloma IX, we compared sodium clodronate with zoledronic acid. The results of this analysis showed that the use of zoledronic acid iv both reduced rates of skeletal related events, as well as being associated with improved rates of overall survival. Although the rate of osteonecrosis of the jaw was increased, this only amounted to 3-4% with most cases being mild. Based on this data, we recommend the use of zoledronic acid, but are not specifying this as part of the study protocol.

1.7.2 Thromboprophylaxis

In the Myeloma IX study, participants receiving thalidomide as part of the CTD regimen had a risk of venous thromboembolism (VTE) of approximately 15%, occurring predominately during the first three months of treatment. In that study, no thromboprophylaxis was specified but for high risk participants, it was suggested that participants should be anticoagulated using either warfarin (treatment dose) or low molecular weight heparin (LMWH). More recently guidelines have been developed to govern the use of thromboprophylaxis. These guidelines suggest that aspirin may be useful in low risk patients but the impact of this is unknown. For high risk patients, anticoagulation with either prophylactic or full dose LMWH is suggested, but again, the impact of these interventions is not fully understood. The use of fixed dose warfarin has been suggested but suffers from inter-patient variability and the need to monitor the dose used. Patients may be classified as high or low risk, based on a number of clinical features (Appendix K).

1.8 BIOLOGICAL FACTORS

A number of staging systems and prognostic factors have been developed in myeloma. In the UK, ß2m has proved to be the most useful and widely used of these approaches. However, like most of the other systems, it is a surrogate marker and ignores the biology of the tumour. More biologically-based approaches have been used with some success. Recurrent cytogenetic changes have been explored as prognostic markers with 13q-, t(11;14), t(4;14) and 17p- , which are possibly associated with different clinical outcomes. Other areas which have been developed as prognostic factors are inherited genetic variants, single nucleotide polymorphisms (SNPs). This current study provides a backdrop against which these findings can be further investigated and aims to extend the findings of the previous study, Myeloma IX.

A number of important scientific studies will be performed, subject to funding:

1. Genetic alterations including fluorescent in situ hybridisation (FISH), copy number sensitive polymerase chain reaction (PCR) for known translocations, copy number changes relevant to myeloma pathogenesis and known prognostic factors previously occurring in more than 5% of cases will be analysed. These changes include: t(4;14), t(11;14), other translocations, 17p-, 16q-, 8p-, 1q+, 1p32-, del(13), 11q- together with deletion and amplification affecting the NFkB pathway.

2. Mutational analysis will be carried out for inactivating mutations at genes with loss of heterozygosity (LOH) including genes involved in the NFkB pathway including BIRC2/3, CYLD, TRAF2/3.

3. Validation of gene expression signatures defined in the previous study as being important predictors of OS, PFS and response to therapy. The aim is to define robust clinically validated signatures, which can be incorporated into clinical practice using limited arrays or antibody based technology.

4. Explore epigenetic changes at specific gene locus as well as validating experimentally defined chromatin state maps identified in experimental preclinical studies currently being developed.

5. Assessment of Minimal Residual Disease MRD based on paraprotein and flow cytometry to determine the depth of response and association with outcome in each arm.

1.8.1 FISH-based cytogenetics

We have developed both cytogenetic and FISH-based approaches investigating targets occurring at a frequency that are able to define worthwhile prognostic groups. These include chromosomal translocation into the Ig locus t(4;14) (10%), t(11;14) (20%), t(16;14), t(6;14) and t(8;14) (10%), together with interstitial deletions and loss of 11q (10%), del13 (40%), 17p- (10%), 1p- and 1q+. Recent reports have suggested that the newer agents may be effective even if a poor prognostic abnormality is present.

1.8.2 Molecular monitoring

Previous studies, including MRC Myeloma VII and IX, have shown that the achievement of a CR is associated with a trend to improved disease-free and overall survival. Following the

paraprotein using electrophoresis and immunofixation is the conventional approach to disease monitoring. In Myeloma IX we performed a more detailed examination of the bone marrow and peripheral blood using flow cytometry, together with free light chain analysis to follow the paraprotein. Analysis of these results is awaited, and these studies will be extended in the current study with the intention of validating our earlier results and examining in more detail subgroups defined by trial pathway.

1.9 SUMMARY AND RATIONALE FOR THERAPEUTIC STUDY

Against the background of the previous MRC trials and worldwide data on the treatment of multiple myeloma, a number of key points emerge and have been summarised below:

1.9.1 Intensive treatment pathway

- For younger patients, a number of studies have supported the idea that oral thalidomide combinations are better induction regimens prior to HDT and have led to the replacement of infusional VAD. In the UK, cyclophosphamide, thalidomide and dexamethasone (CTD) has become the standard approach against which new treatments should be assessed.

- Lenalidomide has been shown to be highly effective in the treatment of myeloma at relapse and also proved to be an effective and safe induction treatment prior to HDT. We have carried out both a pilot study and dose-finding study of the combination lenalidomide, cyclophosphamide and dexamethasone (RCD) and found it well tolerated and highly effective. Therefore RCD will be compared with the UK standard CTD.

- Both the CTD and RCD regimens contain an alkylating agent (cyclophosphamide) which offers exposure to a known active anti-myeloma agent early in the clinical course of the treatment, optimising response rates. Previous experience has shown that pulsed cyclophosphamide is not deleterious to subsequent treatment.

- Recent data have suggested that concomitant proteasome/IMiD combinations are effective therapy. A third induction regimen CCRD will be available to those participants entering the intensive pathway containing an alkylating agent, steroid, IMiD and proteasome inhibitor.

1.9.2 Non-intensive treatment pathway

- The combination of melphalan, prednisolone and thalidomide (MPT) is likely to be taken up widely as the standard approach for patients not destined for transplantation.

- MPT is relatively toxic and difficult to deliver. Results from Myeloma IX demonstrate CTDa is better tolerated and is more effective than MP. Therefore, in this trial, attenuated cyclophosphamide-thalidomide-dexamethasone (CTDa) will be compared with a regimen likely to increase response rates, attenuated lenalidomide-cyclophosphamide-dexamethasone (RCDa).

1.9.3 Both treatment pathways

- The proteasome inhibitor bortezomib has a novel mechanism of action, inducing responses in cases resistant to standard treatment, therefore, we will investigate whether giving bortezomib plus dexamethasone and cyclophosphamide (VCD) to patients who achieve a sub-optimal response (<VGPR) with standard treatment (CTD(a)/RCD(a)) can

increase response rates. Those patients who are randomised to CCRD will not be treated with VCD and will receive CCRD to maximum response.

- Historically maintenance therapy has been explored using a number of different agents in myeloma, but none have had widespread uptake because of a number of differing reasons. The agents tried include alkylating agents, steroids and interferon however, either the clinical benefit has been small or the side effect profile has limited their use. This situation has changed with the development of the IMiD drugs including thalidomide and lenalidomide, both of which have the potential to modify the behaviour of residual clonal cells after the induction of remission in a favourable fashion. In our previous study Myeloma IX, we have shown a significant favourable impact of thalidomide on PFS, but the side effect profile of thalidomide was such that patients only were able to remain on therapy for a median of 7 months which is inappropriately short for a maintenance therapy, and so the standard against which to compare other drugs remains no maintenance.

- Three studies have been presented in abstract suggesting an important clinical benefit for the use of maintenance lenalidomide in newly diagnosed myeloma, in both younger and older patients. The MM015 study, in transplant ineligible patients, showed that continuing lenalidomide after induction with MPR significantly prolonged PFS. The IFM 2002 study using a dose of 10 mg of lenalidomide as maintenance after HDT with autologous stem cell rescue dramatically improved PFS with some suggestion of a benefit for OS. Thus, while the data for maintenance lenalidomide is clear for it generating a significant improvement in PFS there remains some uncertainty around its impact on OS.

- The important follow on question that needs to be addressed is whether the beneficial effects of lenalidomide alone can be enhanced further by the use of an agent with which it may have synergy. (Vorinostat has features which suggest such synergy). Consequently we seek to compare the benefits of lenalidomide alone or in combination with vorinostat over the current standard which is no maintenance. A further comparison group of participants who enter the trial under Pv 6.0 will be randomised to lenalidomide versus close observation.

- To understand the impact of molecular variants on these differing questions.

- The majority of patients with myeloma are now given long-term bisphosphonates; as part of the protocol we suggest that patients should be treated with zoledronic acid, but this is not a pre-requisite to a patient being included in the study.

- Patients at high risk of thromboembolism (Appendix K), particularly those with a previous venous thromboembolic event (VTE) or who are immobile, should be considered for full anticoagulation during induction chemotherapy, either with treatment-dose warfarin or low molecular weight heparin, depending on clinicians' preference. Patients at low risk of a VTE should be considered for aspirin prophylaxis during induction chemotherapy at the treating clinicians' discretion.

2. AIMS AND OBJECTIVES

Myeloma XI is intended to be a unifying trial addressing issues in participants of all ages and providing a strategy within which to introduce new treatments as they become available. There are, however, two distinct treatment pathways.

i. **Intensive pathway:** For younger/fitter participants where intensive HDT with stem cell support is considered appropriate.
ii. **Non-intensive pathway:** For older/less fit participants where standard-dose chemotherapy is considered appropriate.

2.1 THERAPEUTIC QUESTIONS WITHIN THE INTENSIVE PATHWAY

To compare a thalidomide-containing regimen (CTD) and a lenalidomide-containing regimen (RCD) with a 4-drug combination including both lenalidomide and carfilzomib (CCRD), as induction treatment prior to HDT, with respect to response, overall/progression-free survival and response.

2.2 THERAPEUTIC QUESTIONS WITHIN THE NON-INTENSIVE PATHWAY

To compare an attenuated thalidomide-containing regimen (CTDa) with an attenuated lenalidomide-containing regimen (RCDa), with respect to overall/progression-free survival and response.

2.3 THERAPEUTIC QUESTIONS ACROSS BOTH PATHWAYS

- In participants randomised to receive CTD(a) or RCD(a), to assess response to a novel agent, bortezomib with cyclophosphamide and dexamethasone (VCD), in participants whose response to induction treatment is sub-optimal (<VGPR).

- To compare the efficacy of lenalidomide and, for those participants who entered the trial prior to Pv 6.0 only, lenalidomide combined with vorinostat, versus no maintenance.

- To investigate prognostic factors for outcome

2.4 SUB-STUDY OBJECTIVE

2.4.1 Evaluation of the effect of IMiDs on EBV lifecycle in plasma from multiple myeloma participants in the Myeloma XI trial

Objective: To determine EBV reactivation status in plasma samples from SPM patients and associations with protocol treatment.

Further information about this sub-study can be found in Appendix M.

2.5 FURTHER TRANSLATIONAL OBJECTIVES

Blood, bone marrow and urine samples will be required at diagnosis and at key time-points in follow-up for biochemical, cytogenetic, molecular/genetic and immunophenotypic assessments, as part of scientific studies with the following aims:

- To verify the prognostic relevance of cytogenetic FISH abnormalities as prognostic factors for participants exposed to IMiD (immunomodulatory) or proteaseome inhibitor drugs and identify new abnormalities using modern and developing scientific techniques
- To verify gene expression array prognostic signatures identified in MRC Myeloma IX for use as new classification and outcome predictors To follow residual disease as defined by paraprotein, serum free light chain and flow cytometry to define depth and quality of responses

3. TRIAL DESIGN

This is a pragmatic, randomised, phase III, multi-centre, parallel group design, open labelled trial of thalidomide, lenalidomide, carfilzomib and bortezomib combinations and maintenance lenalidomide (+/- vorinostat for participants entered into the trial prior to PV6.0 only) in newly diagnosed patients with symptomatic myeloma.

For initial treatment, thalidomide in combination with cyclophosphamide and dexamethasone, the UK gold standard, will be compared with the newer combination of lenalidomide, cyclophosphamide and dexamethasone. A third treatment regimen, containing lenalidomide, carfilzomib, cyclophosphamide and dexamethasone will be available as induction treatment for those participants in the intensive pathway only. For participants with a sub-optimal response to initial therapy with CTD(a) or RCD(a), the response to the proteasome inhibitor bortezomib will be assessed, as previous studies have demonstrated that it is able to induce responses and improve progression-free and overall survival in participants resistant to standard chemotherapy. Participants young and fit enough to tolerate an autologous transplant will then proceed to high dose melphalan with peripheral blood stem cell rescue. The value of lenalidomide maintenance and lenalidomide combined with vorinostat maintenance compared to no maintenance will then be assessed for participants entered into the trial prior to Myeloma XI Pv 6.0 only. The value of lenalidomide maintenance versus close observation will be assessed for participants who entered the trial under Pv 6.0.

4. ELIGIBILITY

Please note eligibility waivers to inclusion/exclusion criteria are not permitted.

4.1 INCLUSION CRITERIA FOR INITIAL RANDOMISATION

Participants with the following characteristics are eligible for this trial:

- Aged 18 years or greater.
- Newly diagnosed as having symptomatic multiple myeloma or non-secretory multiple myeloma (see Appendix A for definitions) based on:
  - *Paraprotein (M-protein) in serum and/or urine.*
  - *Bone marrow clonal plasma cells or plasmacytoma.*
  - *Related organ or tissue impairment and/or symptoms considered by the clinician to be myeloma related.*
- Provide written informed consent.
- Women of childbearing potential and male participants whose partner is a woman of child bearing potential must be prepared to use contraception in accordance with (and consent to) the Celgene-approved process for thalidomide and lenalidomide Risk Management and Pregnancy Prevention, or commit to absolute and continuous abstinence (true abstinence is acceptable when this is in line with the preferred and usual lifestyle of the subject. Periodic abstinence [e.g. calendar, ovulation, symptothermal or post-ovulation methods] and withdrawal are not acceptable methods of contraception.). Contraception must be used during treatment and for 3 months following bortezomib or cyclophosphamide treatment.
- Women of child bearing potential must have a negative pregnancy test performed by a healthcare professional in accordance with the Celgene-approved process for thalidomide and lenalidomide Risk Management and Pregnancy Prevention. Two methods of reliable contraception must be used, this must include one highly effective method and one additional effective (barrier) method. FCBP must be referred to a qualified provider of contraceptive methods if needed. Examples of highly effective and additional effective methods of contraception are listed in Appendix G.

4.2 EXCLUSION CRITERIA FOR INITIAL RANDOMISATION

Participants with the following characteristics are ineligible for this trial:

- Asymptomatic myeloma (Appendix A).
- Solitary plasmacytoma of bone (Appendix A). (Participants with previous solitary plasmacytoma now progressed to symptomatic or non-secretory myeloma are eligible).
- Extramedullary plasmacytoma (without evidence of myeloma).
- Previous or concurrent active malignancies.
- Documented diagnosis of Myelodysplastic Syndrome (MDS).
- Previous treatment for myeloma, *except* the following:
  - *Local radiotherapy to relieve bone pain or spinal cord compression*
  - *Prior bisphosphonate treatment*
  - *Corticosteroids*

- Known history of allergy contributable to compounds containing boron or mannitol.
- Grade 2 or greater (NCI criteria) peripheral neuropathy.
- Acute renal failure (unresponsive to up to 72 hours of rehydration, characterised by creatinine >500 µmol/L or urine output <400 mL/day or requirement for dialysis).
- Lactating or breastfeeding.
- Patient has active or prior hepatitis C.

Please note: caution is advised in participants with a past history of ischaemic heart disease, pericardial disease, acute diffuse infiltrative pulmonary disease or psychiatric disorders, evidence of impaired marrow function or elevated liver function tests, but exclusion is essentially to be at the discretion of the treating clinician.

4.3 INCLUSION CRITERIA FOR RANDOMISATION TO BORTEZOMIB, CYCLOPHOSPHAMIDE AND DEXAMETHASONE

Participants with the following characteristics are eligible for randomisation to bortezomib-cyclophosphamide-dexamethasone (see Appendix C for response definitions):

- Completed a minimum of 4 cycles of CTD or RCD as per their initial randomised treatment allocation in the intensive pathway or 6 cycles of CTDa or RCDa as per their initial randomisation allocation in the non-intensive pathway in accordance with Myeloma XI protocol.
- At maximal response, showing a partial response or minimal response at the end of their randomised induction treatment.

4.4 EXCLUSION CRITERIA FOR RANDOMISATION TO BORTEZOMIB, CYCLOPHOSPHAMIDE AND DEXAMETHASONE

Participants with the following characteristics are ineligible for randomisation to bortezomib-cyclophosphamide-dexamethasone (see Appendix C for response definitions):

- Received any other anti-myeloma treatment, apart from their initial randomised treatment allocation in Myeloma XI. (Participants who have received local radiotherapy to relieve bone pain or spinal cord compression are eligible).
- Participants in the intensive pathway randomised to receive CCRD at induction.
- Showing complete response (CR), very good partial response (VGPR), no change (NC), progressive disease or relapse.
- Pregnant, lactating or breastfeeding, or women of childbearing potential and male participants whose partner is a woman of child bearing potential unprepared to use contraception or commit to absolute and continuous abstinence during treatment and for 3 months afterwards.
- Previous or concurrent active malignancies.
- Documented diagnosis of Myelodysplastic Syndrome (MDS).

4.5 INCLUSION CRITERIA FOR MAINTENANCE RANDOMISATION

Participants with the following characteristics are eligible for randomisation to maintenance treatment:

- Completed randomised induction treatment (a minimum of 4 cycles of CTD, or CCRD, a minimum of 6 cycles of CTDa or RCDa and, if required according to response /

randomisation allocation, VCD for a maximum of 8 cycles) in accordance with Myeloma XI protocol
- Reached maximal response to randomised induction chemotherapy
- Received at least 100 mg/m^2 high-dose melphalan if entered into the Intensive pathway

4.6 EXCLUSION CRITERIA FOR MAINTENANCE RANDOMISATION

Participants with the following characteristics are ineligible for randomisation to maintenance treatment:

- Failed to respond (PD or NC) to lenalidomide (RCD(a) / CCRD) induction
- Failed to respond (NC) to all protocol treatment (i.e. no response achieved since trial entry)
- Received any other anti-myeloma treatment, apart from their randomised treatment allocations
- Progressive disease (PD) or relapse from CR. (Note: increase in size of lytic lesions on radiological investigation and/or development of hypercalcaemia automatically places participants in the progressive disease category)
- Pregnant, lactating or breastfeeding, or women of childbearing potential and male participants whose partner is a woman of child bearing potential unprepared to use contraception in accordance with the Celgene approved process for lenalidomide Risk Management and Pregnancy Prevention, or commit to absolute and continuous abstinence
- Previous or concurrent active malignancies
- Documented diagnosis of Myelodysplastic Syndrome (MDS).

5. RECRUITMENT AND CONSENT

5.1 RECRUITMENT

Research centres will be required to have obtained local management approval and undertaken a site initiation meeting with the CTRU prior to the start of recruitment into the trial.

The Myeloma XI trial comprises two treatment pathways and, at the outset, participants must be assigned to one of the following treatment pathways:

i. **Intensive treatment pathway** - younger/fitter participants where high-dose therapy with autologous stem cell transplant is considered appropriate.
ii. **Non-intensive treatment pathway** - older/less fit participants not suitable for high-dose therapy and autologous stem cell transplant.

Participants will be defined as younger/fitter or older/less fit, based on their age and general fitness. Strict age restrictions have been deliberately avoided to prevent fit older participants being denied intensive therapy. As a general rule, participants aged ≤60 years will enter the intensive (younger/fitter) pathway; those ≥70 will enter the non-intensive (older/less fit) pathway. Participants aged 60-70 will be eligible for intensive or non-intensive therapy. The treatment pathway will be decided on an individual participant basis, based on a combination of their performance status, clinician judgement and participant preference.

5.2 INFORMED CONSENT PROCESS

The majority of participants who are candidates for the Myeloma XI trial will be identified at the time they are referred to the haematology out-patient department with suspected myeloma. A minority of participants may be identified during in-patient admissions. Invitation to participate in the trial will be made either during their first consultation, when routine diagnostic tests will be performed and potential treatment options discussed, or at the time they receive their diagnostic test results.

To avoid the need for repeat sampling after a diagnosis of myeloma has been confirmed, on suspicion of myeloma, bone marrow samples should be collected and sent to the central research laboratories at the same time as the local diagnostic procedures are performed. Participants must consent to this on the standard NHS consent form for investigations or treatment. The central genetics laboratory will process and store the sample, but no investigations will be performed until the participant signs the Myeloma XI consent form agreeing to genetic laboratory investigations. Blood and urine samples must not be obtained or sent to central laboratories until trial consent has been received.

Potential participants will be provided with a full verbal explanation of the trial and a trial summary sheet. If they are interested in the trial potential participants will then be given a Participant Information Sheet and Informed Consent Document for either the intensive pathway or the non-intensive pathway for consideration. This will include information about the rationale, design and personal implications of the trial. Following information provision, participants will have as long as they need to consider participation (normally a minimum of 24 hours) and will be given the opportunity to discuss the study with their family and other

healthcare professionals before they are asked whether they would be willing to take part in the study.

Assenting patients will then be invited to provide informed, written consent, and be formally assessed for eligibility. The local Principal Investigator (PI) retains overall responsibility for the informed consent of participants at their site and must ensure that any person delegated responsibility to participate in the informed consent process is duly authorised, trained and competent to participate according to the ethically approved protocol, principles of Good Clinical Practice (GCP) and Declaration of Helsinki 1996. If taking informed consent is delegated to another clinically qualified member of the trial team they must have received Good Clinical Practice (GCP) training and be approved by the Principal Investigator, as documented on the Authorised Personnel Log. Informed consent must be obtained prior to the participant undergoing procedures that are specifically for the purposes of the study and are out-with standard routine care at the participating site. The right of a participant to refuse participation without giving reasons must be respected. The participant must remain free to withdraw at any time from the study without giving reasons and without prejudicing his/her further treatment and will be provided with a contact point where he/she may obtain further information about the trial.

Participants who refuse permission for central laboratory investigations are not precluded from entering the therapeutic part of the trial, but it is hoped that the number of participants opting out will be minimal.

Where a participant is required to re-consent or new information is required to be provided to a participant it is the responsibility of the PI to ensure this is done in a timely manner and according to any timelines requested by the CTRU.

A record of the consent / re-consent process detailing the date of consent and all those present will be kept in the participant notes. The original consent form will be retained in the Investigator Site File, a copy of the consent form will be given to the participant, a second copy filed in the hospital notes (as per local practice) and a third copy will be returned to the Clinical Trials Research Unit (CTRU), at the University of Leeds.

After randomisation, participants will be given the appropriate information sheet(s) to allow them to gain knowledge of the treatment to which they have been randomised:

i. Advice for participants receiving thalidomide
ii. Advice for participants receiving lenalidomide
iii. Advice for participants receiving carfilzomib
iv. Advice for participants receiving vorinostat

5.2.1 Loss of Capacity Following Informed Consent

Where valid informed consent is obtained from the participant, and the participant subsequently becomes unable to provide ongoing informed consent by virtue of physical or mental incapacity, the consent previously given when capable remains legally valid.

Participants who lose capacity after informed consent has been obtained will continue with protocol treatment and assessments in consultation with the Principal Investigator and participant's carer / family with the participant's best interests foremost in the decision making process. Ongoing collection of safety and follow-up data (where possible) will

continue via the clinical care team for inclusion in the trial analysis in order to preserve the integrity of the trial's analysis and fulfil regulatory requirements specifically for pharmacovigilance purposes.

6. BASELINE INVESTIGATIONS

A number of investigations are required prior to randomisation. Please refer to Section 7 and Appendix E for further information.

To avoid the need for repeat sampling after a diagnosis of myeloma has been confirmed, on suspicion of myeloma, bone marrow samples should be collected and sent to the central research laboratories at the same time as the local diagnostic procedures are performed. Participants must consent to this on the standard NHS consent form for investigations or treatment.

Investigators must ensure that all investigations to confirm eligibility are performed prior to randomisation. All other baseline investigations must be performed prior to starting protocol treatment, with the exception of the axial skeletal survey, which may be performed up to 2 weeks after starting treatment. The results of these investigations will provide a baseline for day-to-day clinical care of participants.

Baseline disease assessments (paraprotein, serum free light chains and urinary light chains) used to assess response should be the highest value measured before starting treatment.

7. RANDOMISATION PROCEDURES

Eligibility must be confirmed and informed written consent for entry into the trial must be obtained prior to initial randomisation.

Randomisation will be performed by an authorised member of staff at the trials research site using the centralised CTRU automated 24-hour telephone system. Authorisation codes and PINs, which will be provided by the CTRU after site initiation, will be required to access the randomisation system.

Participants will potentially undergo three randomisations:

i. At presentation (randomised to induction chemotherapy (CTD(a) vs RCD(a) vs CCRD (intensive pathway only)).

ii. For those participants whose response to induction treatment with CTD(a) or RCD(a) is sub-optimal i.e. MR or PR (randomised to VCD vs nothing).

iii. After induction and consolidation therapy (where received), eligible participants will then undergo maintenance randomisation (randomised to lenalidomide, lenalidomide and vorinostat or no maintenance N.B. lenalidomide plus vorinostat maintenance is only available for those participants who were entered into the trial prior to PV6.0). NB: See Section 9.3.1.2 (page 50) for ongoing treatment details for participants randomised to the lenalidomide + vorinostat arm.

7.1 INITIAL RANDOMISATION – INDUCTION CHEMOTHERAPY

The following information will be required at randomisation:

- Unique authorisation code and PIN
- Hospital name and UKCRN site code
- Name of person randomising the participant
- Basic participant details including initials, sex, date of birth, and NHS number
- Chosen treatment pathway, i.e. intensive or non-intensive (see Section 5.1)
- Confirmation of eligibility, including a negative pregnancy test (if woman of childbearing potential)
- Confirmation of written informed consent
- Stratification factors (see list below)

Direct line for 24-hour initial randomisation<br>0113 343 1469<br>**Please ensure you have completed F02 Eligibility for Initial Randomisation and F03 Initial Randomisation to Induction Chemotherapy CRFs before phoning**

Participants will be randomised (prior to any treatment being given) on a 1:1:2 basis to **RCD, CTD or CCRD** in the intensive pathway and on a 1:1 basis to **RCDa** or **CTDa** in the non-

intensive pathway and will be allocated a trial number. Allocation will use a computer-generated minimisation algorithm that incorporates a random element to ensure treatment groups are well-balanced for the following characteristics, details of which will be required for randomisation:

- Centre
- Beta-2 microglobulin (<3.5, 3.5–<5.5, ≥5.5 mg/L, unknown)
- Haemoglobin (<115, ≥115 g/L for males; <95, ≥ 95 g/L for females)
- Corrected serum calcium (<2.6, ≥2.6 mmol/L)
- Serum creatinine (<140, ≥140 μmol/L)
- Platelets (<150, ≥150x10^9/L)

Immediately after randomisation, please enter the participant's trial number on the consent form and fax with F03 Initial Randomisation to Induction Chemotherapy CRF to CTRU.

Fax consent forms to: 0113 343 6427

7.2 BORTEZOMIB-CYCLOPHOSPHAMIDE-DEXAMETHASONE (VCD) RANDOMISATION

Eligible participants whose maximal response to CTD(a) or RCD(a) is sub-optimal (MR or PR) will be randomised on a 1:1 basis to VCD or nothing.

Refer to Sections 4.3 and 4.4 for eligibility criteria for this VCD randomisation.
The following information will be required at randomisation:

- Unique authorisation code and PIN
- Hospital name and UKCRN site code
- Name of person randomising the participant
- Participant trial number
- Date of birth
- Confirmation of eligibility
- Level of maximum response achieved (PR or MR)

The randomisation will be stratified by centre, treatment group allocated by the first randomisation and response to initial treatment.

Direct line for 24-hour VCD randomisation

0113 343 5029 (for participants entered into the trial prior to PV6.0)

0113 343 1469 (for participants entered into the trial under PV6.0 or later)

Please ensure you have completed F06 Eligibility for Randomisation to Consolidation Chemotherapy CRF before phoning

7.3 MAINTENANCE RANDOMISATION

Following an assessment of maximal response to induction and/or consolidation therapy, eligible participants will be randomised as follows:

- If entering the trial from Pv 6.0 randomisation will be to no maintenance or lenalidomide on a 1:2 basis.
- If entering the trial prior to Pv 6.0 randomisation will be to lenalidomide, lenalidomide and vorinostat or no maintenance. NB: See Section 9.3.1.2 (page 50) for ongoing treatment details for participants randomised to the lenalidomide + vorinostat arm.

Refer to Sections 4.5 and 4.6 for eligibility criteria for this maintenance randomisation. Participants who are not eligible will be treated off-protocol at the discretion of the local clinician, but trial follow-up will continue as normal.

The following information will be required at randomisation:

- Unique authorisation code and PIN
- Hospital name and UKCRN site code
- Name of person randomising the participant
- Participant trial number
- Date of birth
- Confirmation of eligibility
- Confirmation of negative pregnancy test (if woman of childbearing potential)

The randomisation will be stratified by centre, and treatment group allocated by the first and second randomisation (if applicable).

Direct lines for 24-hour maintenance randomisation
0113 343 5029 (for participants entered into the trial prior to PV6.0)
0113 343 1469 (for participants entered into the trial under PV6.0 or later)
**Please ensure you have completed the F10 Eligibility for Maintenance Treatment CRF before phoning**

8. TRIAL MEDICINAL PRODUCT MANAGEMENT

Please refer to the Myeloma XI Pharmacy and IMP Study Site Operating Procedure for full details of the trial IMP management requirements, including details of IMP destruction, accountability and disposal records.

8.1 INVESTIGATIONAL MEDICINAL PRODUCTS

Within the trial, the following are classed as Investigational Medicinal Products (IMPs):

Cyclophosphamide (for CTD(a), RCD(a), CCRD and VCD)
Cyclophosphamide oral tablets

Composition: cyclophosphamide monohydrate BP 53.50 mg equivalent to 50 mg anhydrous cyclophosphamide.

Generic ("off the shelf") commercial supplies to be used as determined by individual hospital sites. Please refer to the most recent Summary of Product Characteristics (SmPC) for the brand being used.

Dexamethasone (for CTD(a), RCD(a), CCRD and VCD)
Dexamethasone oral tablets

Composition: 2.0 mg dexamethasone PhEur.

Generic ("off the shelf") commercial supplies to be used as determined by individual hospital sites. Please refer to the most recent SmPC for the brand being used.

Lenalidomide (Revlimid™) (for RCD(a), CCRD and maintenance)
Lenalidomide oral capsules

Composition: 5, 10, 15 and 25 mg Revlimid hard capsules.

Lenalidomide will be supplied by Celgene® at the same cost as thalidomide. Refer to Myeloma XI Pharmacy and IMP Study Site Operating Procedure for lenalidomide ordering procedures. Sites are responsible for labelling and ring-fencing the lenalidomide upon receipt as outlined in the Study Site Operating Procedure.

Sites are permitted to use non-trial ("off the shelf") commercial supplies of lenalidomide. However, note that no refund is available for the use of non-trial supplies of lenalidomide. If non-trial stock is used for a trial patient, this should be marked clearly on the Myeloma XI lenalidomide accountability log and a retrospective order should be placed for the non-trial drug used.

Please refer to the trial supplied Investigator Brochure and the most recent SmPC.

Thalidomide (manufactured by Celgene) (for CTD(a))
Thalidomide 50 mg oral hard capsules
Composition: 50 mg thalidomide

“Off the shelf” commercial supplies to be used. Please refer to the most recent SmPC.

Bortezomib (Velcade™) (for VCD)
Bortezomib for subcutaneous or intravenous administration

Composition: 3.5 mg bortezomib (as a mannitol boronic ester) powder for solution for injection.

“Off the shelf” commercial supplies to be used. Please refer to the most recent SmPC.

Vorinostat (Zolinza™) (for RZ)
Vorinostat 100 mg capsule for oral administration.

Composition: contains 100 mg vorinostat.

Vorinostat capsules are supplied by Merck & Co. Inc., and distributed by a third party clinical services organisation. Refer to Myeloma XI Pharmacy and IMP Study Site Operating Procedure for vorinostat ordering procedures.

Trial-specific stock must be used, which will be labelled as Myeloma XI clinical trial stock prior to distribution to sites. Sites are responsible for ring-fencing the vorinostat upon receipt as outlined in the Study Site Operating Procedure.

Please refer to the trial supplied Investigator Brochure (IB).

Carfilzomib (Kyprolis™) (for CCRD)
Lyophilised carfilzomib for injection

Composition: Lyophilised parenteral drug product in 60 mg single use vials. Upon reconstitution, carfilzomib for injection consists of 2 mg/mL solution.

Supply: Carfilzomib will be supplied solely for use in this trial by Onyx/Amgen free of charge.

Trial-specific stock must be used, which will be labelled as Myeloma XI clinical trial stock (IST-CAR-598) prior to distribution to sites. Sites are responsible for ring-fencing the carfilzomib upon receipt as outlined in the Study Site Operating Procedure.

Please refer to the trial supplied IB and the most recent Onyx supplied document “Instructions for Storage and Use of Lyophilised Carfilzomib for Injection”.

8.1.1 IMP formulation and storage

Formulation and storage of IMPs are in line with the manufacturers’ recommendations. For further details refer to the SmPC/IB for each IMP as detailed above (including the Onyx supplied document “Instructions for Storage and Use of Lyophilised Carfilzomib for Injection” for Carfilzomib).

Lenalidomide, carfilzomib and vorinostat supplied for the trial must be ring-fenced in a separate area to non-trial products and records retained in the Pharmacy Site File noting the location of the storage.

All other IMPs (bortezomib, cyclophosphamide, dexamethasone and thalidomide) will be off the shelf supplies. There is no requirement to ring-fence off the shelf general hospital supplies of these IMPs.

8.1.2 IMP preparation

All IMPs will be prepared and handled in line with manufacturers' recommendations. Cytotoxics requiring reconstitution will be reconstituted under conditions approved by the hospital pharmacy.

8.1.3 IMP labelling and handling

Lenalidomide, carfilzomib and vorinostat supplies will contain a study specific label, in line with Directive 2001/20/EC and the Medicines for Human Use (Clinical Trials) Regulations 2004 (amended 2006). The pharmacy will be responsible for completing individual participant details on each label.

Pharmacy will be responsible for labelling cyclophosphamide and dexamethasone in accordance with the requirements of the Medicines for Human Use (Marketing Authorisations Etc.) Regulations 1994.

The CTRU will provide instructions on the labelling requirements for thalidomide, bortezomib and lenalidomide (non-trial stock) for use in accordance with the requirements of the Medicines for Human Use (Clinical Trials) Regulations 2004 (and amended in 2006), the application of which pharmacy will be responsible for.

Please refer to the Myeloma XI Pharmacy and IMP Study Site Operating Procedure for full details of the trial IMP management requirements, including record keeping.

8.1.4 IMP administration

The responsibility for prescription and administration of treatment ultimately remains with the Principal Investigator.

<u>Lenalidomide</u>
Lenalidomide capsules should be taken at about the same time each day. The capsules should not be opened, broken or chewed. The capsules should be swallowed whole, preferably with water, either with or without food. If less than 12 hours has elapsed since missing a dose, the participant can take the dose. If more than 12 hours has elapsed since missing a dose at the normal time, the participant should not take the dose, but take the next dose at the normal time on the following day.

<u>Carfilzomib</u>
Intravenous hydration will be given immediately prior to each dose of carfilzomib during Cycle 1. This will consist of 250 to 500 mL normal saline or other appropriate iv fluid. If lactate dehydrogenase (LDH) or uric acid is elevated (and/or in participants considered still at risk for TLS) at Cycle 2 Day 1, then the recommended iv hydration should be given additionally before each dose in Cycle 2. The goal of the hydration program is to maintain robust urine output (eg ≥ 2 L/day).

If the participant has a dedicated line for carfilzomib administration, the line must be flushed with a minimum of 20 mL of normal saline prior to and after drug administration.

Carfilzomib will be given as an iv infusion and for doses > 27 mg/m^2, carfilzomib should be infused over 30 minutes. The dose will be administered at a facility capable of managing hypersensitivity reactions. Participants will remain at the clinic under observation for at least 1 hour following each dose of carfilzomib in Cycle 1 and following the dose on Cycle 2 Day 1. During these observation times, post dose iv hydration (between 250 mL and 500 mL normal saline or other appropriate iv fluid formulation) can be given. Participants should be monitored periodically during this period for evidence of fluid overload, and frusemide administered as appropriate.

Dexamethasone will be administered *prior* to all carfilzomib doses during the 1st cycle. If during their first cycle, patients have not received their treatment defined dexamethasone dose (40 mg po unless dose reduced due to toxicity) prior to their carfizomib infusion, dexamethasone 4 mg po/iv will be given prior to their carfilzomib infusion. The full protocol defined treatment dose of dexamethasone should still be given in addition to this. If a participant is receiving an alternative corticosteroid (e.g. methylprednisolone) due to toxicity an equivalent dose should be given prior to their carfilzomib infusion.

If treatment-related fever, rigors, chills, and/or dyspnea are observed post any dose of carfilzomib after the first cycle, a minimum dose of dexamethasone (4 mg po/iv) should be administered prior to subsequent doses of carfilzomib. In most cases it is expected that this will be the protocol defined treatment dose of dexamethasone (usually 40mg po unless dose reduced due to toxicity).

Cardiac failure events have been reported in some patients receiving carfilzomib. Participants should be monitored for cardiac events and prompt action taken as necessary.

All participants should be routinely evaluated for hypertension and treated as needed. Dose modifications are in Appendix H.

Carfilzomib will be dose capped at a body surface area of 2.2 m^2.

<u>Vorinostat</u>
Vorinostat should be taken in the evening either with food, or within 0 to 30 minutes of a meal. It is suggested that participants take vorinostat at approximately the same time of day, for consistency. Participants should not break, chew or open capsules. If a dose of vorinostat is missed, it should be taken as soon as possible on the same day. If it is missed for the entire day, it should not be made up. NB: See Section 9.3.1.2 (page 50) for ongoing treatment details for participants randomised to the lenalidomide + vorinostat arm.

<u>Bortezomib</u>
Please refer to the SmPC.

8.2 NON-INVESTIGATIONAL MEDICINAL PRODUCTS

Within the trial, the following is classed as Non-Investigational Medicinal Product (NIMPs):

- Melphalan

9. STUDY TREATMENT

9.1 INTENSIVE PATHWAY TREATMENT

9.1.1 Intensive pathway outline

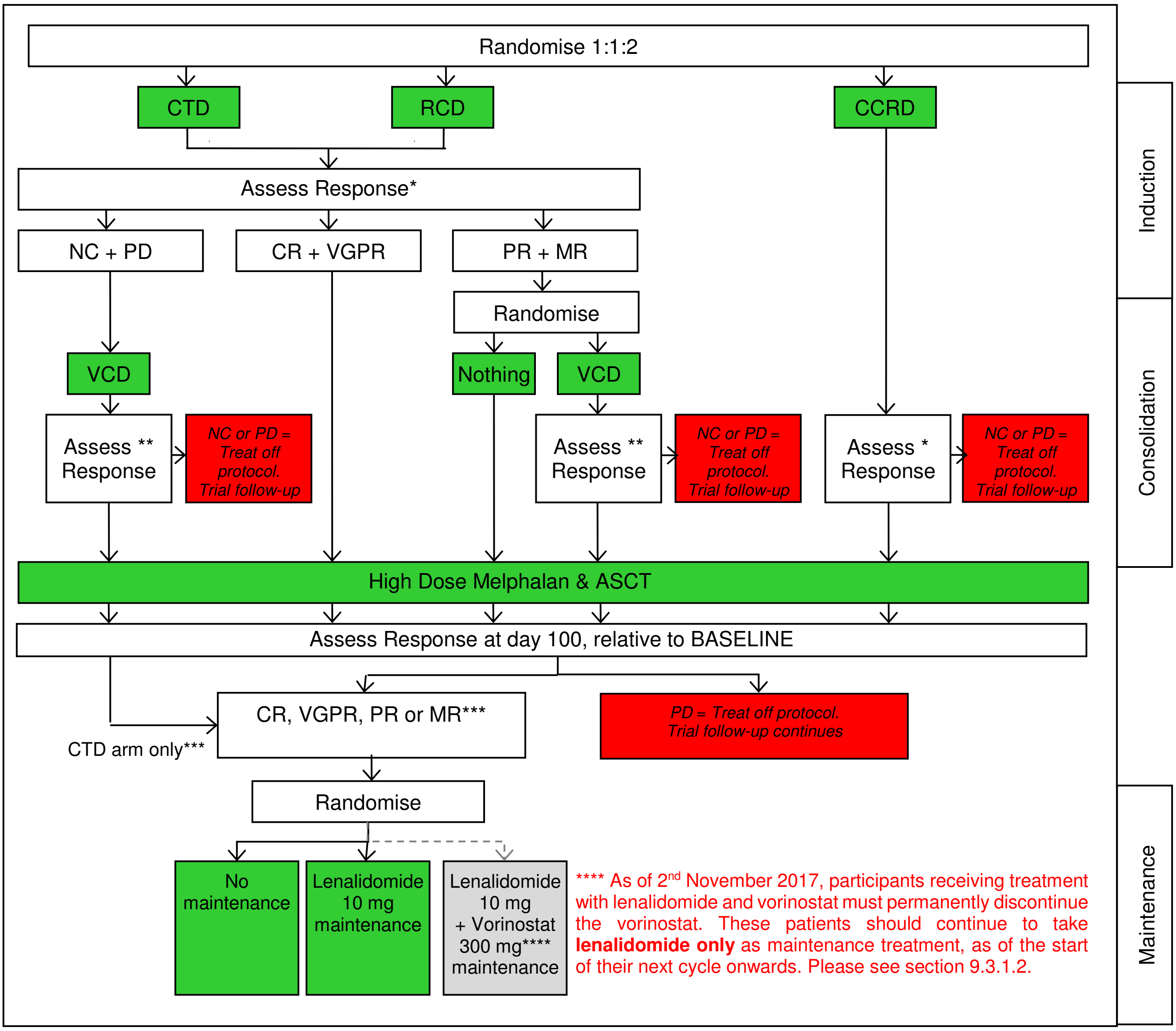

** In the absence of disease progression participants should receive a minimum of 4 cycles of induction chemotherapy and should continue to maximum response or intolerance. Participants showing progressive disease at any time during treatment with CTD or RCD should proceed to VCD. Participants showing progressive disease at any time during CCRD treatment should be treated off protocol and followed up for the purposes of the trial.*
*** In the absence of disease progression participants should receive up to a maximum of 8 cycles of VCD and should continue to maximum response or intolerance.*
**** Participants entered into the RCD or CCRD arms and assessed as NC or PD at the end of induction are not eligible for maintenance randomisation.*
***** Lenalidomide plus vorinostat maintenance is only available for those participants who entered into the trial prior to protocol version 6.0. NB: See Section 9.3.1.2 (page 50) for ongoing treatment details for participants randomised to the lenalidomide + vorinostat arm.*

9.1.2 Induction chemotherapy treatment

Participants will be randomised to receive either CTD, RCD, or CCRD.

9.1.2.1 Cyclophosphamide, thalidomide, and dexamethasone (CTD) regimen

**Days 1, 8, 15 (i.e. weekly)**	Cyclophosphamide 500 mg po
**Continuously**	Thalidomide 50 mg hard capsules; initially 100 mg daily po for 3 weeks, increasing to 200 mg daily po
**Days 1-4 and 12-15**	Dexamethasone 40 mg daily po

The cycle is repeated every 21 days. Response should be assessed after each cycle and, in the absence of disease progression, participants should continue therapy until maximum response (minimum 4 cycles) or intolerance.

- Participants showing CR or VGPR at maximum response will proceed to high dose melphalan and ASCT.
- Participants showing PR or MR at maximum response will proceed to VCD randomisation.

Participants showing NC after 4 cycles or PD at any time during induction will all receive VCD (i.e. will not undergo VCD vs nothing randomisation)

9.1.2.2 Lenalidomide, cyclophosphamide, and dexamethasone (RCD) regimen

**Days 1 and 8**	Cyclophosphamide 500 mg po
**Days 1-21**	Lenalidomide 25 mg daily po
**Days 1-4 and 12-15**	Dexamethasone 40 mg daily po

The cycle is repeated every 28 days. Response should be assessed after each cycle and in the absence of progression, participants should continue therapy until maximum response (minimum 4 cycles) or intolerance.

- Participants showing CR or VGPR at maximum response will proceed to high dose melphalan and ASCT.
- Participants showing PR or MR at maximum response will proceed to VCD randomisation.
- Participants showing NC after 4 cycles or PD at any time during induction will all receive VCD (i.e. will not undergo VCD vs nothing randomisation).

9.1.2.3 Carfilzomib, cyclophosphamide, lenalidomide, & dexamethasone (CCRD) regimen

**Days 1 and 8**	Cyclophosphamide 500 mg po
**Days 1 and 2, 8 and 9, 15 and 16**	Carfilzomib 20*/36 mg/m^{2**} iv
**Days 1-21**	Lenalidomide 25 mg daily po
**Days 1-4, 8, 9 and 15, 16**	Dexamethasone 40 mg daily po

* Carfilzomib 20 mg/m^2 is only administered on days 1 and 2 of cycle 1
** Carfilzomib will be dose capped at a body surface area of 2.2 m^2

Carfilzomib should be administered as detailed in Section 8.1.4. The cycle is repeated every 28 days. Response should be assessed after each cycle and in the absence of progression, participants should continue therapy until maximum response (minimum 4 cycles).

- Participants showing CR, VGPR, PR or MR at maximum response will proceed to high dose melphalan and ASCT.
- Participants showing NC after 4 cycles or PD at any time during induction will be treated off protocol and followed up for the purposes of the trial only.

9.1.2.4 Prescribing and pregnancy testing

Prescribing of thalidomide 50 mg hard capsules
This must be done according to the Celgene Risk Management Programme. Refer to Investigator Site File and Pharmacy Site File for further details.

Prescribing of lenalidomide
This must be done in accordance to the Celgene Risk Management Programme. Refer to the Investigator Site File and Pharmacy Site File for further details.

Under normal circumstances a maximum of 28 days lenalidomide supply should be prescribed. In exceptional circumstances a request for extended prescribing (greater than 28 days supply) may be made to the CTRU. Sites should ensure written agreement is attained from the CTRU prior to each occasion of extended prescribing.

Prescribing of carfilzomib
Patients should be adequately hydrated and iv hydration should be given according to the Investigator Brochure. In participants considered to be at risk for TLS, oral hydration should be continued in further cycles as required by the participants' medical condition and at the investigators discretion. See Section 8.1.4 for further details.

Pregnancy testing and contraception
For all chemotherapy treatments women of childbearing potential (WCBP) (see Appendix G) must have a negative pregnancy test performed by a healthcare professional in accordance with the Celgene-approved thalidomide / lenalidomide Pregnancy Prevention Programme:

- Before starting treatment, on the day of the study visit or in the 3 days prior to the study visit
- Every 3 or 4 weeks during treatment (prior to each cycle), including 4 weeks after the end of study treatment

All protocol treatment is to be discontinued immediately if a pregnancy in a female participant occurs or is suspected and the participant instructed to return any unused portion of the medication to the investigator (see Section 11.4 for further information). Contraception must also continue to be used for 3 months following cyclophosphamide treatment.

9.1.2.5 Dose modifications for CTD, RCD, and CCRD

Dose modifications for CTD, RCD, and CCRD are detailed in Appendix H. Dose modifications and delays different from those stated in the protocol, for management of toxicities are at the discretion of the investigator.

9.1.3 Consolidation chemotherapy treatment

9.1.3.1 Bortezomib, cyclophosphamide, and dexamethasone (VCD) regimen

Participants demonstrating NC or PD to their randomised induction regimen (CTD(a) / RCD(a) only), or who demonstrated MR or PR and subsequently randomised to receive VCD will receive VCD treatment as follows:

**Days 1, 4, 8 and 11**	Bortezomib 1.3 mg/m^2 sc or iv
**Days 1, 8, 15**	Cyclophosphamide 500 mg orally
**Days 1-2, 4-5, 8-9 and 11-12**	Dexamethasone 20 mg daily orally

The cycle is repeated every 21 days. Response should be assessed after each cycle and, in the absence of disease progression, should continue to maximum response or intolerance (up to maximum of 8 treatment cycles). If CR is achieved then only a further two treatment cycles should be administered. There must be at least 72 hours between each bortezomib dose.

Varicella prophylaxis with Aciclovir is also recommended as per local practice.

Contraception must be used during treatment and for 3 months following bortezomib or cyclophosphamide treatment.

Dose modification of VCD

Dose modifications for VCD are detailed in Appendix I.

9.1.4 Stem cell mobilisation and harvest

PBSC harvest should commence after the participant has completed their induction and consolidation (if applicable) treatment. Participants who respond (MR, PR, VGPR or CR) to RCD/CTD +/- VCD or CCRD should proceed to stem cell mobilisation and harvest.

Participants showing PD or NC during CTD or RCD induction will all receive VCD then proceed to stem cell mobilisation, harvest and high-dose melphalan with stem cell rescue. Participants showing PD or NC during induction chemotherapy (RCD/CTD) and who subsequently show NC/PD following VCD should be treated off-trial at the local clinician's discretion. These participants will be followed up for the purposes of the trial. Participants showing PD or NC during CCRD should also be treated off-trial at the local clinician's discretion and will be followed up for the purposes of the trial.

Participants showing progressive disease or relapse from CR (Appendix C) **after an initial response to protocol induction chemotherapy** (RCD/CTD +/-VCD or CCRD), and prior to high-dose treatment, should be considered for alternative treatment, at the clinician's

discretion off-protocol, as treatment failures. These participants will be followed up for the purposes of the trial.

Stem cell mobilisation and stem cell harvest will be performed according to local practice. As further stem cell-supported therapy may be considered in the relapse setting, the possibility of collecting enough cells to divide into aliquots should be borne in mind.

Inadequate stem cell harvest
Participants who fail to obtain an adequate stem cell harvest should be given high dose melphalan at a reduced dose of 100 mg/m^2 using the same protocol, with no stem-cells re-infused.

9.1.5 High-dose melphalan (HDM) regimen and autologous stem cell transplant

All participants in the intensive pathway, who have responded to initial induction chemotherapy (CTD/RCD +/- VCD or CCRD) will go on to receive HDM and autologous stem cell transplant (ASCT).

High-dose melphalan and ASCT will be given according to local practice.

Adjustment for renal insufficiency
In the presence of renal insufficiency (participants with serum creatinine ≥200 µmol/L, prior to transplant), the dose of melphalan should be reduced.

Serum creatinine	Dose of melphalan (mg/m^2)
<200µmol/L	200
≥200µmol/L	140

Participants who receive at least 100 mg/m^2 HDM (+/- ASCT) and have not demonstrated PD or relapse will proceed to maintenance randomisation.

9.1.6 Maintenance

At approximately day 100 after HDM (+/- ASCT), eligible participants must undergo maintenance randomisation as detailed in Sections 7.3 and 9.3.

9.1.7 Relapsed participants

All participants will continue to be followed up annually until death or the final analysis of survival data as described in Section 15, regardless of the treatment they receive at relapse.

9.2 NON-INTENSIVE PATHWAY TREATMENT

9.2.1 Non-intensive pathway outline

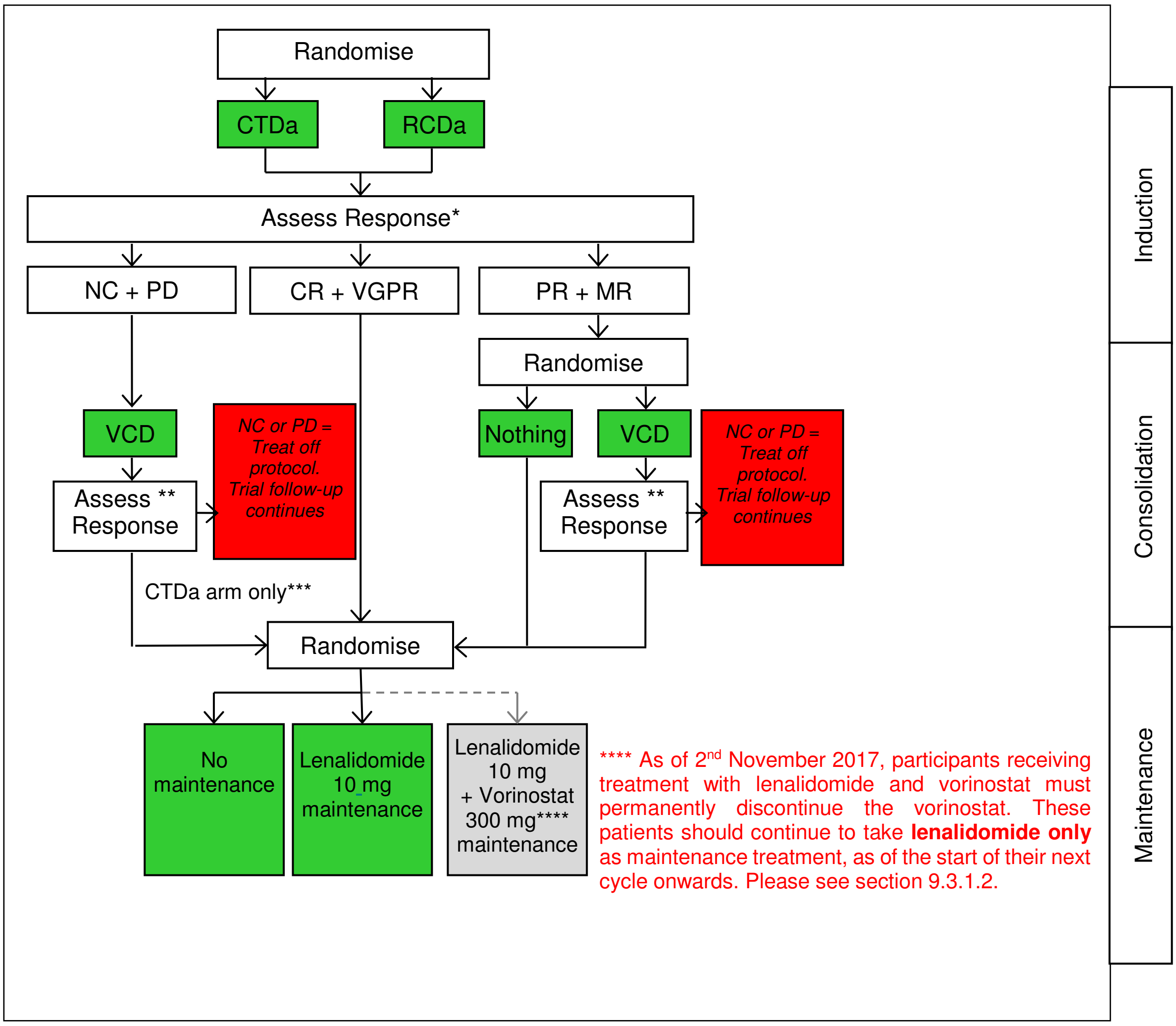

** In the absence of disease progression, and as long as they are responding, participants should receive a minimum of 6 cycles of induction chemotherapy and should continue to maximum response or intolerance. Participants showing NC after 4 cycles or progressive disease at any time during their induction chemotherapy should proceed to VCD*

*** In the absence of disease progression participants should receive up to a maximum of 8 cycles of VCD and should continue to maximum response or intolerance*

**** Participants entered into the RCDa arm and assessed as NC or PD at the end of RCDa induction are not eligible for maintenance randomisation*

**** Lenalidomide plus vorinostat maintenance is only available for those participants who were entered into the trial prior to protocol version 6.0. NB: See Section 9.3.1.2 (page 50) for ongoing treatment details for participants randomised to the lenalidomide + vorinostat arm.*

9.2.2 Induction chemotherapy treatment

Participants will be randomised to receive either CTDa **or** RCDa.

9.2.2.1 Cyclophosphamide, thalidomide, and dexamethasone attenuated (CTDa) regimen

**Days 1, 8, 15, 22 (weekly)**	Cyclophosphamide 500 mg po
**Continuously**	Thalidomide 50 mg hard capsules; initially 50 mg daily po for 4 weeks, increasing every 4 weeks by 50 mg increments to 200 mg daily po
**Days 1-4 and 15-18**	Dexamethasone 20 mg daily po

The cycle is repeated every 28 days. Response should be assessed after each cycle. In the absence of disease progression and as long as they are responding, participants should continue therapy until maximum response (minimum of 6 cycles) or intolerance.

- Participants showing CR or VGPR at maximum response will proceed to lenalidomide maintenance.
- Participants showing PR or MR at maximum response will proceed to VCD randomisation
- Participants showing NC after 4 cycles or PD at any time during induction will all receive VCD (i.e. will not undergo VCD vs nothing randomisation)

9.2.2.2 Lenalidomide, cyclophosphamide, and dexamethasone attenuated (RCDa) regimen

**Days 1 and 8**	Cyclophosphamide 500 mg po
**Days 1-21**	Lenalidomide 25 mg daily po
**Days 1-4 and 15-18**	Dexamethasone 20 mg daily po

The cycle is repeated every 28 days. Response should be assessed after each cycle. In the absence of disease progression and as long as they are responding, participants should continue treatment until maximum response (minimum 6 cycles) or intolerance.

- Participants showing CR or VGPR at maximum response will proceed to lenalidomide maintenance
- Participants showing PR or MR at maximum response will proceed to VCD randomisation
- Participants showing NC after 4 cycles or PD at any time during induction will all receive VCD (i.e. will not undergo VCD vs nothing randomisation)

9.2.2.3 Prescribing and pregnancy testing

<u>Prescribing of thalidomide 50 mg hard capsules</u>
This must be done according to the Celgene Risk Management Programme. Refer to Investigator Site File and Pharmacy Site File for further details.

<u>Prescribing of lenalidomide</u>
This must be done according to the Celgene Risk Management Programme. Refer to Investigator Site File and Pharmacy Site File for further details.

Under normal circumstances a maximum of 28 days lenalidomide supply should be prescribed. In exceptional circumstances a request for extended prescribing (greater than

28 days supply) may be made to the CTRU using the lenalidomide exceptional prescribing request form. Sites should ensure written agreement is attained from the CTRU prior to each occasion of extended prescribing.

Pregnancy testing and contraception
For all chemotherapy treatments, women of childbearing potential (WCBP) (see Appendix G) must have a negative pregnancy test performed by a healthcare professional in accordance with the Celgene approved thalidomide/lenalidomide Pregnancy Prevention Programme:

- before starting thalidomide/lenalidomide, on the day of the study visit or in the 3 days prior to the study visit
- every 4 weeks during treatment, including 4 weeks after the end of study treatment

All protocol treatment is to be discontinued immediately if a pregnancy in a female participant occurs or is suspected and the participant instructed to return any unused portion of the medication to the investigator (see Section 11.4 for further information).

Contraception must continue to be used for 3 months following cyclophosphamide treatment.

9.2.2.4 Dose modifications of attenuated CTD and attenuated RCD

Dose modifications for CTDa and RCDa are detailed in Appendix H.

9.2.3 Consolidation chemotherapy treatment

9.2.3.1 Bortezomib, cyclophosphamide, and dexamethasone (VCD) regimen

**Days 1, 4, 8 and 11**	Bortezomib 1.3 mg/m^2 sc or iv
**Days 1, 8, 15**	Cyclophosphamide 500 mg orally
**Days 1-2, 4-5, 8-9 and 11-12**	Dexamethasone 20 mg daily orally

The cycle is repeated every 3 weeks (21 days). Response should be assessed after each cycle and, in the absence of progression, participants should continue treatment to maximum response (up to a maximum of 8 treatment cycles) or participant intolerance. If CR is achieved then only a further two treatments should be administered. There must be at least 72 hours between each bortezomib dose.

Varicella prophylaxis with acyclovir is also recommended as per local practice.

Contraception must be used during treatment and for 3 months following bortezomib or cyclophosphamide treatment.

Dose modification of VCD
Before each dose, participants should be evaluated for possible toxicities which may have occurred. Dose modifications are as detailed in Appendix I.

9.3 MAINTENANCE (INTENSIVE AND NON-INTENSIVE PATHWAYS)

Upon completion of induction chemotherapy +/- VCD (and HDM sequence in the intensive pathway), eligible participants must undergo a further randomisation as detailed in Sections 4.5, 4.6, and 7.3.

If allocated to receive maintenance it should commence approximately 100 days after HDM sequence (+/- ASCT) for intensive pathway participants and immediately after completing induction treatment for non-intensive pathway participants. Eligible participants should be randomised when they are ready to commence treatment but this must be within 9 months of completing HDM sequence (+/- ASCT) or induction treatment for participants on the non-intensive pathway. Maintenance should only be started when the neutrophil count is ≥1.0 x 10^9/L and platelets ≥100 x 10^9/L.

9.3.1 Maintenance schedules

9.3.1.1 Lenalidomide maintenance

**Days 1-21**	Lenalidomide 10 mg daily po

The cycle is repeated every 28 days (allowing for a 7 day rest period), and in the absence of toxicity, lenalidomide is continued until disease progression.

For participants randomised to lenalidomide maintenance prior to the implementation of Protocol version 5.0:

These participants should remain on their original lenalidomide maintenance dose of 25 mg daily po for 21 days of a 28 day cycle.

9.3.1.2 Lenalidomide Vorinostat (RZ) maintenance (for participants entered into the trial prior to protocol version 6.0 only)

As of 2nd November 2017, participants receiving treatment with lenalidomide and vorinostat must permanently discontinue the vorinostat. These participants should continue to take **lenalidomide only as maintenance treatment, as of the start of their next cycle onwards. They should continue taking lenalidomide up until disease progression, in the absence of non-manageable toxicity.**

**Days 1-21**	Lenalidomide 10 mg daily po
**Days 1-7 and 15-21**	**As of 2nd November 2017, all participants randomised to lenalidomide + vorinostat must permanently discontinue vorinostat on completion of their current treatment cycle. See above**

The cycle is repeated every 28 days, and in the absence of toxicity, continued until disease progression.

9.3.2 Prescribing and pregnancy testing

Prescribing of lenalidomide
This must be done according to the Celgene Risk Management Programme. Refer to Investigator Site File and Pharmacy Site File for further details.

Under normal circumstances a maximum of 28 days lenalidomide supply should be prescribed. In exceptional circumstances a request for extended prescribing (greater than 28 days supply) may be made to the CTRU using the lenalidomide exceptional prescribing request form. Sites should ensure written agreement is attained from the CTRU prior to each occasion of extended prescribing.

Pregnancy testing and contraception
For all chemotherapy, women of childbearing potential (WCBP) (see Appendix G) must have a negative pregnancy test performed by a healthcare professional in accordance with the Celgene approved lenalidomide Pregnancy Prevention Programme:

- Before starting maintenance treatment, on the day of the study visit or in the 3 days prior to the study visit
- Every 4 weeks during treatment, including 4 weeks after the end of study treatment

All protocol treatment is to be discontinued immediately if a pregnancy in a female participant occurs or is suspected and the participant instructed to return any unused portion of the medication to the investigator (see Section 11.4 for further information).

Contraception must continue for 30 days after completing treatment with vorinostat.

9.3.3 Dose reduction schedules for maintenance

Participants should be evaluated before each cycle to confirm suitability for ongoing treatment. A new course of treatment may begin on the scheduled Day 1 of a new cycle if all of the following are met:

- The absolute neutrophil count (ANC) is ≥ 1 x 10^9/L
- The platelet count is ≥75 x 10^9/l *or, dependent on bone marrow infiltration by plasma cells, platelet count is ≥30 x 10^9/l.*
- Any other lenalidomide or vorinostat (RZ arm only) related AE that may have occurred must have resolved to ≤ Grade 1 severity or baseline

If these conditions are not met on day 1 of a new cycle, the subject will be evaluated weekly, and a new treatment cycle will not be initiated until the toxicity has resolved, as described above. If lenalidomide and/or vorinostat was halted during the previous cycle and was restarted with a dose reduction, without requiring an interruption for the remainder of the cycle, that reduced level will be initiated on day 1 of the new cycle. If lenalidomide or vorinostat was omitted for the remainder of the previous cycle, or if the new cycle is delayed due to toxicity encountered on scheduled day 1, then the new cycle will be started with a one-level dose reduction (Appendix I).

Detailed dose reductions for lenalidomide and vorinostat maintenance for both haematologic and non-haematologic toxicity are given in Appendix J.

Dose modifications and delays different from those stated in the protocol, for management of toxicities are at the discretion of the investigator.

9.3.4 Relapsed participants

All participants will continue to be followed up annually until death or the final analysis of survival data as described in Section 15, regardless of the treatment they receive at relapse.

9.4 SUPPORTIVE MEASURES

There are many aspects of the care of patients with myeloma which, although not part of the specific treatment regimens, are of considerable importance. It is assumed that all centres entering participants into the study will be familiar with these. They are set out in the guidelines 'Diagnosis and Management of Multiple Myeloma' compiled by the UK Myeloma Forum on behalf of the British Committee for Standards in Haematology (BCSH).

Initial hydration with the institution of a fluid intake of at least 3 litres prior to starting chemotherapy and maintenance of adequate hydration throughout is of prime importance. Hypercalcaemia often resolves rapidly with vigorous re-hydration without recourse to the ‘acute’ use of bisphosphonates.

Anaemia is one of the commonest complications in patients with myeloma. Approximately half of the patients will have moderate to severe anaemia at diagnosis, and most of the remainder will develop anaemia during the course of their illness. Some of the patients who are anaemic at diagnosis will have an improvement in haemoglobin concentration with the introduction of effective chemotherapy, but in other patients the anaemia persists or worsens. Treatment choices are to transfuse with blood or to treat with recombinant erythropoietin.

Other aspects of management are reviewed in the UKMF guidelines. The avoidance of NSAIDs in patients with renal impairment and use with caution in other patients, the need for timely orthopaedic/neurosurgical intervention particularly in the event of disease affecting the spine/spinal cord, and prompt and appropriate treatment of infection is emphasised. It is contemplated that most centres will have their own regimens and protocols covering these aspects of general management. Liaison with general practitioners is important and treating clinicians should have clear protocols for providing advice, and mechanisms for immediate admission of patients to hospital, if required.

**G-CSF, Human Granulocyte Colony-Stimulating Factor (e.g. Granocyte™ – rHuG-CSF, Lenograstim™ – Chugai Pharma Limited)**	Use as clinically indicated. Dose for autologous transplantation, Peripheral Blood Progenitor Cell Mobilisation (PBPC) or for chemotherapy-induced Neutropenia – 150 µg/m^2/day (or as per local protocol). Granocyte™ is available at contract prices from AAH Hospital Service in the UK.
**Erythropoietin**	Use as clinically indicated.
**Bisphosphonates**	All participants should receive a bisphosphonate. The choice (clodronate, pamidronate or zoledronic acid) is at the discretion of the treating clinician. However, based on the results of the previous study, Myeloma IX, we recommend the use of zoledronic acid until disease progression.
**Blood products**	Use as clinically indicated.
**Thromboprophylaxis**	All participants should receive thromboprophylaxis for at least the first three months of treatment. This should be done according to local guidelines. However, it is suggested that low risk participants be given aspirin (75 mg daily) and high risk participants be given LMWH (Appendix K).
**Tumour lysis syndrome prevention**	Use allopurinol as per local practice.
***Pneumocystis carinii* pneumonia prophylaxis**	As per local practice.
**Varicella prophylaxis**	Use acyclovir as per local practice.
**Antifungal prophylaxis**	As per local practice.
**Anti-emetics**	As per local practice.
**Gastric Irritation**	Use of proton pump inhibitor or H2 antagonist as per local policy.
**Antibiotic prophylaxis**	The value and risks of prophylactic antibiotics are unproven in newly diagnosed myeloma patients. Myeloma XI participants are eligible to enter the TEAMM trial (a randomised placebo controlled trial of levofloxacin once daily for the first 12 weeks from diagnosis).

9.5 CONTRAINDICATED CONCOMITANT MEDICATION

Participants must not receive other anti-cancer therapy or investigational drugs while on this study.

9.6 WITHDRAWAL OF TREATMENT

In line with usual clinical care, cessation or alteration of regimens at any time will be at the discretion of the attending clinicians or participants themselves. All participants withdrawn from treatment or prescribed alternative treatment will still attend for follow-up assessments unless unwilling to do so and case report forms will continue to be collected.

10. LABORATORY INVESTIGATIONS AND DATA COLLECTION

Investigations in this study will combine both local and, where the participant has consented, central assessment.

Full details of local investigations required are detailed in tabular form in Appendix E.

For participants who have consented to central investigations, samples to be sent to central laboratories are detailed in Appendix E. Addresses to which samples for central analysis should be sent are provided in Appendix L. **The central investigations will be performed in relation to the scientific studies described in Section 2.5.**

Completed Case Report Forms (CRFs) should be returned to the Clinical Trials Research Unit at the address given in the Investigator Site File.

Participating sites will be expected to maintain a file of essential trial documentation (Investigator Site File), which will be provided by the CTRU, and keep copies of all completed CRFs for the trial.

It is the responsibility of staff at research sites to obliterate all personal identifiable data on any hospital reports, letters etc. prior to sending to CTRU. Such records should only include trial number, initials and date of birth to identify the participant.

10.1 BASELINE INVESTIGATIONS

To avoid the need for repeat sampling after a diagnosis of myeloma has been confirmed, on suspicion of myeloma, bone marrow samples should be collected and sent for central review at the same time as the local diagnostic procedures are performed. Participants must consent to this on the standard NHS consent form for investigations or treatment. PLEASE NOTE: Only bone marrow samples are permitted to be sent for central review using consent given on the NHS consent form only. Blood and urine samples may only be obtained and sent after consent to participate in the trial has been received.

10.1.1 Local investigations at presentation

Investigators must ensure that all local baseline investigations to confirm eligibility are performed within 4 weeks prior to randomisation (unless otherwise specified). All other baseline investigations must be performed prior to starting protocol treatment, with the exception of the axial skeletal survey, which may be performed up to 2 weeks after starting treatment.

These are investigations required for the establishment and staging of the diagnosis, and to provide a baseline for the clinical care of participants on a day-to-day basis and should include:

- Performance status (see Appendix B for definitions of performance status)
- Physical examination
- Medical history (including a review for any prior cancer history)
- FBC, biochemistry, β_2M, LDH, CRP

- Paraprotein, immunoglobulins, urinary light chain (24hr sample) and serum free light chain assessments. NB: results from prior to initiation of treatment where the paraprotein / light chain values were at their highest should be used to assess response.
- An axial skeletal survey should be performed (see Appendix F). Axial skeletal survey can be supplemented by CT and/or MRI investigation when appropriate. It is permissible to use unenhanced whole body CT in place of the skeletal survey where this is local policy.
- Bone marrow. While we anticipate that these will be done locally to examine bone marrow morphology, it is essential to the success of this study that adequate material is sent centrally for RNA expression analysis, FISH and cytogenetics. If the diagnosis of myeloma is highly likely, samples may be sent centrally in anticipation of trial entry, though sometimes it may be necessary to obtain a second marrow sample before initiating treatment.
- Pregnancy test. Women of childbearing potential (WCBP) (see Appendix G) must have a negative pregnancy test performed by a healthcare professional in accordance with the Celgene thalidomide and lenalidomide Pregnancy Prevention Programmes (on the day of starting thalidomide or lenalidomide or in the 3 days prior to starting study treatment).

10.1.2 Central investigations at presentation

It is important that adequate good-quality bone marrow samples are obtained and sent to the stated destination. Do not waste the majority of sample making an excess of smears.

The investigations to be performed include:

- A baseline assessment of disease comprising creatinine, paraprotein, serum free light chains, serum immunoglobulins, urinary light chain and β_2M.
- Bone marrow aspirates will be used to determine the percentage plasma cells and their phenotype. These samples will also be used for RNA expression, molecular analysis and cytogenetic evaluation.
- A sample of peripheral blood will be converted into DNA and stored for SNP analysis.

10.2 FOLLOW-UP INVESTIGATIONS

Follow up is designed to monitor response to therapy and will be assessed both locally and at critical time-points centrally.

10.2.1 Local follow-up

During treatment, participants should attend clinic for follow-up visits at the end of each cycle of treatment, i.e. every **3 weeks** during CTD and VCD, and every **4 weeks** during CTDa, RCD(a), CCRD, lenalidomide maintenance or RZ maintenance. Participants should be followed up in accordance with local practice during HDM sequence (+/- ASCT). Thereafter, participants should attend clinic for follow-up visits every **2 months** for the first 2 years and 3 monthly thereafter until disease progression. During the initiation of therapy, more frequent haematological assessment will be required according to the SPC(s).

Following 2 years intensive follow-up of the last participant recruited into the trial (last participant under protocol version up to and including version 5.0 and last participant under Pv 6.0 onwards will act as separate triggers for the annual follow-up), data collection at the CTRU will be performed annually.

Refer to Appendix E for details of investigations.

10.2.2 Central follow-up

Testing will be done at a number of critical time points, summarised below:

- To monitor disease progression and residual disease levels; serum paraprotein, serum immunoglobulin level measured by electrophoresis, immunofixation, serum free light chains and BJP.
- The number of plasma cells in the bone marrow will be determined by the use of morphology and flow cytometry.
- At relapse a further bone marrow sample should be sent for the determination of the level of bone marrow plasma cells and RNA expression profiling.

10.2.3 Response and relapse assessment

Response and relapse will be assessed by: clinical symptoms, FBC, paraprotein and free light chain assessments, urinary light chain, bone marrow assessments, and defined using the International Uniform Response criteria in Appendix C. All response categories (CR, VGPR, PR, MR and PD) require 2 consecutive assessments made at any time before the institution of any new therapy. A bone marrow assessment must be done to confirm CR, but confirmation with a repeat bone marrow is not needed. VGPR and CR categories require serum and urine studies regardless of whether disease at baseline was measurable on serum, urine, both, or neither. All categories also require no known evidence of progressive or new bone lesions if radiographic studies were performed. Radiographic studies are not required to satisfy these response requirements. Refer to Appendix C for full criteria.

10.2.4 Toxicity

Toxicity data will be collected to determine the occurrence of trial treatment related events, including thromboembolic events.

10.2.5 Follow up for SPMs

All participants will be followed up for second primary malignancy for the duration of the trial.

10.2.6 Death

At the time of death date and cause of death will be collected.

10.3 DEFINITION OF END OF TRIAL

The end of the trial is defined as the last participant's last data item. Participants will be followed up until death or until the final analysis of survival data as described in Section 15.

11. PHARMACOVIGILANCE PROCEDURES

11.1 GENERAL DEFINITIONS

11.1.1 Adverse events (AEs)

An adverse event is any untoward medical occurrence in a participant or clinical trial subject administered a medicinal product which does not necessarily have a causal relationship with this treatment and can include:

- Any unintentional, unfavourable clinical sign or symptom
- Any new illness or disease or the deterioration of existing disease or illness
- Any clinically relevant deterioration in any laboratory assessments or clinical tests

11.1.2 Serious Adverse Events (SAEs)

A serious adverse event is defined in general as any untoward medical occurrence or effect that:

- Results in death
- Is life threatening*
- Requires in-patient hospitalisation or prolongation of existing hospitalisation
- Results in persistent or significant disability or incapacity
- Results in a congenital anomaly or birth defect
- Other important medical event

For the purposes of this trial, a Second Primary Malignancy (SPM) is reportable as an SAE (please see Section 11.3.2).

*The term life threatening refers to an event in which the participant was at risk of death at the time of the event; it does not refer to an event which hypothetically might have caused death if it was more severe.

Medical judgement should be exercised in deciding whether an AE is serious in other situations. Important AE/ARs that are not immediately life-threatening or do not result in death or hospitalisation but may jeopardise the subject or may require intervention to prevent one or the other outcomes listed in the definition above, should also be considered serious.

11.1.3 Adverse reactions (ARs)

Adverse reactions are all untoward and unintended responses to an IMP related to any dose administered.

11.1.4 Serious Adverse Reaction (SAR)

Where an SAE is deemed to have been related to an IMP used within the trial, the event is termed as a serious adverse reaction.

11.1.5 Suspected Unexpected Serious Adverse Reaction (SUSAR)

A Suspected Unexpected Serious Adverse Reaction (SUSAR) is a serious adverse drug reaction which also demonstrates the characteristic of being unexpected, the nature,

seriousness, severity OR outcome of which is not consistent with the information about the medicinal product as set out in the Reference Safety Information (RSI) (See Section 11.3.2).

11.2 OPERATIONAL DEFINITION AND REPORTING ADVERSE EVENTS AND REACTIONS

Due to the nature of myeloma and its treatment, participants are likely to experience several adverse events throughout the course of the disease. Adverse reactions considered to be related to trial treatment will be collected throughout treatment (up to 30 days after last protocol treatment) on the relevant CRF.

11.2.1 Recording and reporting thromboembolic events

All thromboembolic events (DVT, line-related thrombosis, pulmonary embolism) occurring at any time from randomisation until 30 days after the date of disease progression must be recorded on the Thromboembolic Event CRF and returned to the CTRU via the standard postal system within 7 days of the site becoming aware.

11.2.2 Recording and reporting pregnancies/suspected pregnancies

Pregnancies and suspected pregnancies (including a positive pregnancy test regardless of age or disease status) of a participant or a male participant's partner occurring any time until 3 months post cessation of trial treatment must be reported using the Pregnancy CRF and faxed to the CTRU within 24 hours of the research staff becoming aware of the event (see Section 11.4).

**Fax numbers for reporting pregnancies/suspected pregnancies**<br><br>**CTRU fax number: 0113 343 6427**

Should the event fulfil any of the criteria described in Section 11.1.2 (Serious Adverse Events), a Serious Adverse Event Form should also be completed.

11.3 OPERATIONAL DEFINITION – SERIOUS ADVERSE EVENTS (SAEs)

11.3.1 Events not classed as SAEs

The following events will <u>not</u> be recorded as SAEs within this trial:

- Deaths attributable to myeloma
- Hospitalisation for:
    - *Routine treatment or monitoring of the studied indication not associated with any deterioration in condition*
    - *Treatment which was elective and pre-planned, for a pre-existing condition not associated with any deterioration in condition*
    - *Admission to hospital or other institution for general care, not associated with any deterioration in condition*
    - *Treatment on an emergency, outpatient basis for an event not fulfilling any of the definitions for serious as given above and not resulting in hospital admission*
    - *Disease progression*

11.3.2 Recording and reporting SAEs and SUSARs

Fax numbers for reporting SAEs / SUSARs / Second Primary Malignancies

CTRU fax number: 0113 343 6427

SAEs
All SAEs occurring from the date of randomisation until 30 days after the date of disease progression and SARs/SPMs occurring for the duration of the trial must be recorded on the Serious Adverse Event Form and faxed to the CTRU **within 24 hours** of the research staff becoming aware of the event.

Each SAE will be described by:

- signs/symptoms with a diagnosis, if possible
- case description
- duration (start and end dates; times, if applicable)
- seriousness criteria
- action taken in relation to IMPs
- outcome
- causality, in the opinion of the investigator*
- whether the event would be considered expected or unexpected (Refer to the RSI, details below)*

*Assessment of causality and expectedness must be made by a doctor. If a doctor is unavailable, initial reports without causality and expectedness assessment should be submitted to CTRU by a non-doctor within 24 hours, but must be followed up by medical assessment as soon as possible thereafter.

When determining whether an SAE is expected or not, please refer to the RSI sections in the documents listed in the table below. Please note that you should use the approved versions of the SPCs and IBs supplied by CTRU.

Drug name	IB or SPC	RSI section
Lenalidomide	SPC	RSI in Section 4.4 (Special warnings and precautions for use) and Section 4.8 (Undesirable effects)
Thalidomide	SPC	RSI in Section 4.4 (Special warnings and precautions for use) and Section 4.8 (Undesirable effects)
Cyclophosphamide	SPC	RSI in Section 4.4 (Special warnings and precautions for use) and Section 4.8 (Undesirable effects)
Dexamethasone	SPC	RSI in Section 4.4 (Special warnings and precautions for use) and Section 4.8 (Undesirable effects)
Bortezomib	SPC	RSI in Section 4.4 (Special warnings and precautions for use) and Section 4.8 (Undesirable effects)
Vorinostat	IB, v8.0, 01/08/2013	RSI in Section 7.1 (Reference Safety Information)
Carfilzomib	IB, v16.1, 16th January 2016	RSI in Section 7 and Appendix A (Additional information for the Investigator and Company Core Safety Information (CCSI))

Please ensure that each SAE is reported separately and not combined on one SAE form. The original SAE/SUSAR Report(s) should be retained by site until the event has reached a final outcome and all queries have been resolved (as determined by CTRU). When requested, please return original (wet-ink) initial and follow-up reports to CTRU and retain copies at site.

Any follow-up information should be faxed to CTRU as soon as it is available. Changes in SAE outcome should be reported as soon as this is known. Events will be followed up until the event has resolved or a final outcome has been reached. Investigators must report all SAEs to their host institution in line with their local arrangements.

Second primary malignancies (SPMs)
All new / second primary malignancies or suspected malignancies occurring from the date of randomisation for the **duration of the trial** must be recorded on the SPM CRF in addition to the SAE CRF, and faxed to the CTRU **within 24 hours** of the research staff becoming aware of the event. Once all resulting queries have been resolved, the CTRU will request the original form to be posted and a copy to be retained on site.

SUSARs
All SAEs assigned by the local investigator (or following central review) as both suspected to be related to IMP-treatment and unexpected will be classified as SUSARs and will be subject to expedited reporting to the MHRA. The CTRU will inform the MHRA, the main REC and the Sponsor of SUSARs within the required expedited reporting timescales.

All SUSARs must be recorded on the SUSAR CRF and faxed to the CTRU within 24 hours of the research staff becoming aware of the event. SUSARs are reportable for the duration of the trial.

The original SAE/SUSAR Report(s) should be retained by site until the event has reached a final outcome and all queries have been resolved (as determined by CTRU). When requested, please return original (wet-ink) initial and follow-up reports to CTRU.

11.4 PREGNANCIES OR SUSPECTED PREGNANCIES

Pregnancy in participants on thalidomide or lenalidomide or their partners must be prevented as effectively as possible. The Celgene approved thalidomide and lenalidomide pregnancy prevention programme must be followed as per usual clinical practice. Contraception must also be used during treatment and for 3 months following bortezomib or cyclophosphamide treatment.

All protocol therapy must be stopped immediately if a pregnancy in a female participant occurs or is suspected. Participants must be instructed to return any unused portion of the medication to the investigator. Participants withdrawn from treatment will still attend for follow-up assessments unless unwilling to do so and case report forms will continue to be collected.

Female participants should be referred to an obstetrician/gynaecologist experienced in reproductive toxicity for further evaluation and counselling. If a pregnancy occurs in a male participant's partner, the partner should be advised to consult her GP or gynaecologist as soon as possible. CTRU will report the pregnancy or suspected pregnancy to Celgene, Merck and/or Onyx/Amgen as applicable.

Pregnant patients must be followed until the end of their pregnancy and the CTRU must be notified of the outcome of the pregnancy (including false-positive pregnancy tests) within 24 hours of this information being known. If a pregnancy occurs in a male participant's partner,

details of the pregnancy will still be collected where possible. The outcome of the pregnancy must be notified to CTRU.

The outcome of any pregnancy which qualifies as a SAE (i.e. spontaneous or therapeutic abortion, foetal and neonatal death, or congenital abnormalities – including those detected in an aborted foetus), or the death of an infant which occurs in connection with in utero exposure to the study drugs must be reported to the CTRU in accordance with Section 11.3.2.

The local Principal Investigator shall be responsible for any decision regarding the continued participation in the study of patients who, after an initial positive pregnancy diagnosis, are confirmed as no longer being pregnant.

11.5 RESPONSIBILITIES

Local Principal Investigator:

- Checking for SAEs when participants attend for treatment/follow up.
- Medical judgement in assigning to SAEs, seriousness, causality and expectedness.
- To ensure all SAEs are recorded and reported to the CTRU within 24 hours of becoming aware and to provide further follow up information as soon as available.
- To report SAEs to local committees in line with local arrangements.

CTRU:

- Expedited reporting of SUSARs to Competent Authority (MHRA in UK), main REC and sponsor within required timelines.
- Preparing annual safety reports in collaboration with appropriate members of the TMG to Competent Authority, main REC, periodic safety reports to TSC and DMEC as appropriate.
- Notifying Investigators of SUSARs that occur within the trial.
- Reporting SAEs to the relevant drug companies involved in the trial.

Chief Investigator (or nominated individual in CI's absence):

- To assign causality and expected nature of SAEs where it has not been possible to obtain local assessment.
- To review all SAEs.
- To review all events assessed as SUSARs in the opinion of the local investigator. In the event of disagreement between local assessment and sponsor review with regards to SUSAR status, local assessment will not be overruled, but sponsor may add comments prior to reporting to MHRA.

DMEC:

In accordance with the Trial Terms of Reference for the DMEC, periodically reviewing unblinded overall safety data to determine patterns and trends of events, or to identify safety issues, which would not be apparent on an individual case basis.

TSC:

In accordance with the Trial Terms of Reference for the TSC, periodically reviewing safety data and liaising with the DMEC regarding safety issues.

12. CRITERIA OF RESPONSE

Disease progression and response to treatment will be assessed according to the modified International uniform response criteria for multiple myeloma (refer to Appendix C) using locally and (where available) centrally analysed blood, urine and bone marrow samples, unless progression of myeloma occurs as an isolated bone lesion, growth of a plasmacytoma or an increase in plasma cells in the bone marrow without a change in M-protein, where tissue histological examination will be performed.

All response categories (CR, VGPR, PR, MR and PD) require 2 consecutive assessments made at any time before the institution of any new therapy. A bone marrow assessment must be done to confirm CR, but confirmation with a repeat bone marrow is not needed. VGPR and CR categories require serum and urine studies regardless of whether disease at baseline was measurable on serum, urine, both, or neither. All categories also require no known evidence of progressive or new bone lesions if radiographic studies were performed. Radiographic studies are not required to satisfy these response requirements.

13. ENDPOINTS

13.1 PRIMARY

- Overall survival
- Progression-free survival

13.2 SECONDARY

- Response, including CR rate at the end of induction and including conversion rate to CR/VGPR for participants who undergo bortezomib-cyclophosphamide-dexamethasone randomisation
- Toxicity
- PFS2
- Relevant biological endpoints

13.3 STUDY DEFINITIONS

Overall survival is defined as the time from the date of initial randomisation to the trial to the date of death from any cause or last follow-up. If a participant is still alive at the time of analysis or lost to follow-up before death is documented, they will be censored at the last date known alive. Participants discontinuing protocol treatment, receiving non-protocol treatment or suffering a second malignancy will still be followed for overall survival unless they explicitly withdraw consent. Overall survival for VCD randomisation comparisons is defined similarly from the date of VCD randomisation. Overall survival for maintenance randomisation comparisons is defined similarly from the date of maintenance randomisation.

Disease progression will be determined according to the Modified International Uniform Response criteria of Response and Progression (based on Blade et al, 1998; Durie et al, 2006; Rajkumar et al, 2011, Appendix C). It is not possible for a participant to progress whilst receiving protocol induction chemotherapy (CCRD, CTD, CRD, CTDa or CRDa treatment); this is refractory disease rather than progression. Progression-free survival for induction chemotherapy comparisons is defined as the time from the date of initial randomisation to the trial to the date of progression or death from any cause. Participants who do not progress will be censored at the last date they were known to be alive and progression-free. Participants discontinuing protocol treatment, receiving non-protocol treatment or suffering a second malignancy will still be followed for progression-free survival unless they explicitly withdraw consent. Progression-free survival for VCD randomisation comparisons is defined similarly from the date of VCD randomisation. Any participants who have been found to progress prior to entering the VCD randomisation will be censored at the date of VCD randomisation. Progression-free survival for maintenance randomisation comparisons is defined similarly from the date of maintenance randomisation. Any participants who have been found to progress prior to entering the maintenance randomisation will be censored at the date of maintenance randomisation.

Response rates will be determined according to the Modified International Uniform Response criteria of Response and Progression (based on Blade et al, 1998; Durie et al, 2006; Rajkumar et al, 2011, Appendix C) using local responses based on samples of blood, urine and bone marrow and other clinical assessments.

PFS2 is defined as the time from date of initial randomisation to the date of second documented disease progression (or the start of third line of anti-myeloma treatment) or date

of death from any cause, whichever first. Participants alive and for whom a second progression has not been observed will be censored at the last date they were known to be alive and second progression-free. PFS2 for VCD randomisation comparisons is defined similarly from the date of VCD randomisation. Any participants who have been found to progress prior to entering the VCD randomisation will be censored at VCD randomisation. PFS2 for maintenance randomisation comparisons is defined similarly from the date of maintenance randomisation. Any participants who have been found to progress prior to entering the maintenance randomisation will be censored at maintenance randomisation.

Toxicity will be reported based on adverse events, as graded by CTCAE V4.0 and determined by routine clinical assessments at each centre

14. STATISTICAL CONSIDERATIONS

14.1 SAMPLE SIZE

Summaries of sample size calculation are given in Table 1 for Pv 2.0- Pv 5.0 and Table 2 for Pv 6.0 and later. Further detail is provided in the remainder of this subsection.

14.1.1 Protocol version 2.0 - Protocol version 5.0

14.1.1.1 Intensive pathway

To demonstrate an increase in median survival from 66 months on the standard therapy (CTD) to 84 months (hazard ratio = 0.79) with RCD would require observing 545 events, with the recruitment of 1183 patients.

These numbers will also enable detection of a six-month increase in PFS from 29 months to 35 months when 893 PFS events have been observed. These calculations and those which follow assume time to progression and survival times follows an exponential distribution, a 2-sided 5% level of significance, 80% power, and allow for a 5% dropout, with a 4-year recruitment and 4 year follow-up period. The standard therapy estimates are taken from MRC Myeloma IX. These changes in OS and PFS are reasonable estimates of what can be expected clinically based on what is known of the impact of lenalidomide use at relapse setting and thalidomide at presentation.

14.1.1.2 Non-intensive pathway

To demonstrate an increase in median survival from 33 months on the standard therapy (CTDa) to 42 months (hazard ratio = 0.79) with RCDa would require observing 545 events, with the recruitment of 787 participants. These numbers will also enable detection of a four-month increase in PFS from 15 months to 19 months when 637 PFS events have been observed. These calculations also assume a 2-sided 5% significance level, 80% power, and allow for a 5% dropout, with a 4-year recruitment and 4 year follow-up period. The standard therapy estimates are again taken from MRC Myeloma IX. These changes in OS and PFS are reasonable estimates of what can be expected clinically based on what is known of the lenalidomide use at relapse setting and thalidomide at presentation.

14.1.1.3 Combined analysis across both pathways

To demonstrate a 9 month increase in median survival from, say, 48 months on standard therapy (CTD(a)) to 57 months with RCD(a) (hazard ratio = 0.84), with the combined total of 1970 participants (1138 events), would give about 82% power, again assuming a 2-sided 5% level of significance, and allowing for a 5% dropout. Although the two pathways are distinct, if they both show a similar effect of RCD(a), it will be appropriate to pool the data in this way. Note that the number of events required is slightly more than in the power calculations for the two pathways separately (545 each, 1090 in total) as assuming a smaller survival difference implies less improvement in the experimental arms which translates into more events.

14.1.1.4 Bortezomib, cyclophosphamide and dexamethasone versus nothing comparison

For the VCD comparison, approximately 47% of participants were estimated to be eligible in each pathway (based on data from MRC Myeloma IX), and would be randomised to VCD or nothing, resulting in about 556 randomised participants in the intensive pathway and 370 in the non-intensive pathway, making 926 participants in both pathways combined. It is

expected that this step produces at least a 15% conversion rate of these PR/MR participants to CR/VGPR. To ensure that the conversion rate is not less than 15%, the numbers of conversions will be checked after particular numbers of participants have been entered (in both pathways combined), to see if this is still consistent with this 15% conversion rate, using exact probabilities as used for the initial stage of a Gehan two-stage design (Gehan, 1961). To have 95% power that the conversion rate is not less than 15% at least 1 response in the first 19 participants and 3 responses in the first 50 participants would need to be observed. If less than these numbers of conversions are observed after the entry of these numbers of participants, an alternative trial strategy may be considered.

However, assuming this randomisation continues, there will be adequate numbers of participants (in each pathway separately) to detect with high power (>90%) at least a 10% increase in conversion rate between the VCD and no treatment arms – assuming a minimal 5% conversion rate in the ‘nothing’ arm, i.e. a postulated 15% conversion rate in the VCD arm as compared to 5% in the no consolidation treatment arm.

If the randomisation is not halted up to this point, a further interim analysis for the bortezomib comparison will be performed after 400 participants have had their conversion rates evaluated. The intention of this interim analysis is to stop this randomisation at this point if it has been firmly established that the conversion rate is less than 15%. With 200 participants randomised to VCD this would occur if there had been less than 22 conversions from these 200 participants (using a Gehan two-stage design approach, as above). A further intention for this interim analysis is to evaluate the conversion rate and PFS in this cohort for the possibility of an exceptionally large effect of bortezomib on either conversion rate or PFS, for example, a 50% conversion rate with bortezomib, or an increase of 24 months in median PFS with bortezomib, from a median of 21 months to 45 months. In the latter case, using a significance level of 0.005 for this interim analysis, based on the O’Brien and Fleming (1979) alpha spending function, which suggests an alpha level of 0.047 for the final analysis and 0.005 for the interim analysis, we will have approximately 80% power to detect such an increase in PFS.

For the final analysis, using PFS curves by response from MRC Myeloma IX as a baseline comparator, the power of this VCD treatment to detect an improvement in PFS through a likely increase in PFS in the PR+MR response categories has also been calculated. 476 participants would be necessary to detect a nine-month increase in PFS in the intensive pathway, from a median of 26 months to a median of 35 months, with 80% power, so with 556 participants we have more than 80% power for this comparison. In the non-intensive pathway we have 90% power to detect a six-month increase in PFS from a median of 14 months to a median of 20 months.

14.1.1.5 Maintenance comparison

<u>Lenalidomide maintenance versus nothing comparison</u>

The maintenance comparison for participants reaching maintenance randomisation under Pv 2.0- Pv 4.0 includes a two-way randomisation at maintenance. This maintenance comparison is powered on demonstrating a 10% increase in 5-year survival in the participants treated with lenalidomide as compared to no treatment. MRC Myeloma IX demonstrated that approximately 50% of participants over both pathways will reach the maintenance randomisation and it is assumed that a similar percentage will reach maintenance in this trial. Expected survival in those not given maintenance is of the order of 50% at 5 years. Under an exponential survival model, to show a 10% increase in 5 year survival in participants treated with lenalidomide would imply a median survival of about 81 months in this group. To demonstrate such an increase, assuming a 2-sided 5% level of

significance, 90% power, allowing for a 5% dropout, and with a 4-year recruitment and 3¼ year follow-up period, would require observing 474 events, with the recruitment of 1080 participants.

<u>Lenalidomide maintenance versus lenalidomide and vorinostat versus nothing comparison</u>
The maintenance comparison for participants reaching maintenance randomisation under Pv 5.0 includes a three-way randomisation at maintenance. This is a 1:1:1 randomisation to lenalidomide and vorinostat vs. lenalidomide alone vs. no maintenance. With assumed 5-year survivals of 65%, 55% and 50% respectively in the three arms and a total estimated recruitment of approximately 1000 participants this gives 90% power for the 10% increase in 5 year survival in the participants treated with lenalidomide and vorinostat compared with no maintenance treatment, and 78% power for the 10% increase in 5 year survival comparing lenalidomide and vorinostat. These power calculations assume an exponential survival distribution, 2-sided 5% levels of significance, a 2% dropout rate, 3¼-year recruitment and 4 year follow-up periods, and median survivals for the lenalidomide and vorinostat, lenalidomide alone and control groups of 96.5, 69.6 and 60 months respectively.

14.1.2 Protocol version 6.0

The extension of recruitment in the Myeloma XI trial gives greater power to answer existing questions relating to thalidomide, lenalidomide and bortezomib combinations in induction chemotherapy and questions regarding lenalidomide in maintenance therapy. In addition, questions related to a 4-drug regimen containing carfilzomib in the intensive pathway are added to the aims of the trial. Updates to the sample size calculation are described below and in Table 2. Overall, is anticipated that 1044 further participants will be recruited to the intensive pathway and 607 further participants will be recruited to the non-intensive pathway. In summary, a total of 4396 participants will be recruited to the Myeloma XI trial (2556 participants in the intensive pathway and 1840 participants in the non-intensive pathway).

14.1.2.1 Intensive pathway

To demonstrate a 20-month increase in median survival from 66 months on the 3-drug therapies (CTD and RCD) to 86 months (hazard ratio = 0.79) with CCRD would require observing 466 events, with the recruitment of 1044 patients.

These numbers will also enable detection of a 7-month increase in PFS from 30 months to 37 months when 703 PFS events have been observed. These calculations assume time to progression and survival times follow an exponential distribution, a 2-sided 5% level of significance, 80% power, and allow for a 5% dropout, with a 2-year recruitment and 4 year follow-up period.

If CTD or RCD is superior and comparisons are undertaken against only one of these arms, the power to show the 7 month increase in PFS or the 20 month increase in OS reduces to approximately 60%, although we would have approximately 80% power to detect a difference of 9-months in PFS and a difference of 26 months in OS.

With respect to the 3-drug comparison there will be 2074 patients allocated equally to CTD and RCD. No adjustments are made for this increase sample size with respect to power or clinically relevant difference assessable. However, it is anticipated that the event-driven comparison (545 events) will now be after a shortened period of follow-up than previously anticipated.

14.1.2.2 Non-intensive pathway

Increasing recruitment in the non-intensive pathway to 1840 participants means that there is the potential to detect powerfully smaller clinically relevant differences in PFS and OS under similar assumptions (a 2-sided 5% level of significance, 80% power, and allow for a 5% dropout, with a 4-year recruitment and 4 year follow-up period).

To demonstrate an increase in median survival from 33 months on the standard therapy (CTDa) to 39 months (hazard ratio = 0.85) with RCDa would require observing 1149 events, with the recruitment of 1604 participants. These numbers will also enable detection of a 3-month increase in PFS from 15 months to 18 months (hazard ratio = 0.83) when 1264 PFS events have been observed.

14.1.2.3 Combined analysis across both pathways

No updates are made to sample size calculations for combined analysis across both pathways in Pv 6.0.

14.1.2.4 Implications for the bortezomib, cyclophosphamide and dexamethasone versus no treatment comparison

Accrual to the VCD comparison has been lower than anticipated prior to Pv 6.0 (approximately 10 participants per month). Continuing recruitment into the intensive and non-intensive pathways should allow the important questions to be powerfully answered even at this slower accrual rate. Assuming randomisation continues at a similar rate into Pv 6.0 there will be adequate numbers of participants in the intensive pathway to detect with high power (>90%) at least a 10% increase in conversion rate between the VCD and no treatment arms – assuming a minimal 5% conversion rate in the ‘nothing’ arm. Similarly, for the non-intensive pathway there will be >80% power to detect a 15% increase in conversion rate.

For the final analysis, in the intensive pathway 476 participants would be necessary to detect a 9-month increase in PFS in the intensive pathway, from a median of 26 months to a median of 35 months (hazard ratio = 0.74) when 361 PFS events have been observed. Similarly, in the non-intensive pathway 180 participants yields approximately 80% power to detect an 8-month increase in PFS from a median of 14 months to a median of 22 months (hazard ratio = 0.64) when 154 PFS events have been observed.

Table 1: Summary of sample size calculations for comparisons in the Myeloma XI study (Pv 2.0 - Pv 5.0)

Pathway	Comparison	Endpoint	Standard therapy median (months)	Experimental therapy median (months)	Increase (months)	Recruitment (months)	Follow-up (months)	Drop-out (months)	Hazard ratio	Power	Number of events	Number of participants randomised
**Induction**												
I	CTD vs. RCD	OS	66	84	18	48	48	5%	0.79	80%	545	1183
I	CTD vs. RCD	PFS	29	35	6	48	48	5%	0.83	80%	893	1204
NI	CTDa vs. RCDa	OS	33	42	9	48	48	5%	0.79	80%	545	787
NI	CTDa vs. RCDa	PFS	15	19	4	48	48	5%	0.79	80%	567	637
I+NI	CTD(a) vs. RCD(a)	OS	48	57	9	48	48	5%	0.84	82%	1138	1970
**VCD**												
I	VCD vs. nothing	PFS	26	35	9	48	48	5%	0.74	80%	361	476
NI	VCD vs. nothing	PFS	14	20	6	48	48	5%	0.70	90%	337	380
**Maintenance**												
I+NI	Len. vs. nothing	PFS	20	26.7	6.7	39	48	5%	0.75	90%	509	1014
I+NI	Len. vs. nothing	OS	60*	69.6*	9.6	39	48	2%	0.74	90%	458	1014
I+NI	Len. vs. Len+Vor.	PFS	26.7	34	7.7	39	48	2%	0.76	80%	539	707
I+NI	Len. vs. Len+Vor.	OS	69.6*	96.5*	26.9	39	48	2%	0.72	78%	285	707

** Correspond to 5-year survivals of 50% (60 months median survival), 55% (69.6 month median survival) and 65% (96.5 months median survival)*

Table 2: Summary of sample size calculations for comparisons in the Myeloma XI study (Pv 6.0)

Pathway	Comparison	Endpoint	Standard therapy median (months)	Experimental therapy median (months)	Increase (months)	Recruitment (months)	Follow-up (months)	Drop-out (months)	Hazard ratio	Power	Number of events	Number of participants randomised
**Induction**												
I	CTD vs. RCD	OS	66	84	18	48	48	5%	0.79	80%	545	1183
I	CTD vs. RCD	PFS	29	35	6	48	48	5%	0.83	80%	893	1204
I	CCRD vs. CTD and RCD	OS	66	86	20	24	48	5%	0.77	80%	466	1044
I	CCRD vs. CTD and RCD	PFS	30	37	7	24	48	5%	0.81	80%	703	1044
NI	CTDa vs. RCDa	OS	33	39	6	48	48	5%	0.85	80%	1149	1604
NI	CTDa vs. RCDa	PFS	15	18	3	48	48	5%	0.83	90%	1264	1421
I+NI	CTD(a) vs. RCD (a)	OS	48	57	9	48	48	5%	0.84	82%	1203	1970
**VCD**												
I	VCD vs. nothing	PFS	26	35	9	48	48	5%	0.74	80%	361	476
NI	VCD vs. nothing*	PFS	14	22	8	48	48	5%	0.64	80%	154	180
**Maintenance**												
I	Len. vs. nothing	PFS	22	37	15	39	48	2%	0.60	90%	156	340
NI	Len. vs. nothing	PFS	18	28	10	39	48	2%	0.64	90%	215	400
I+NI	Len. vs. nothing	OS	*60	*72	12	48	39	2%	0.84	80%	1057	1900
I	Len. vs. nothing	OS	81	105	24	48	39	2%	0.77	80%	475	1260
NI	Len. vs. nothing	OS	38	50	12	48	39	2%	0.76	80%	428	640

** Correspond to 5-year survivals of 50% (60 months median survival) and 56% (72 month median survival)*

14.1.2.5 Lenalidomide versus no maintenance comparison

The increase in recruitment to the Myeloma XI study will allow important questions concerning lenalidomide maintenance therapy to be powerfully answered in the intensive and non-intensive pathways separately, in addition to previously planned comparisons in pathways combined. Lenalidomide and vorinostat is discontinued as an allocated maintenance treatment in Pv 6.0 due to a withdrawal of drug supply by the manufacturer.

Combined analysis across both pathways

Assuming that approximately 50% of participants entering the Myeloma XI study enter the maintenance randomisation we will have approximately 2200 participants randomised in total. The allocation ratio between no maintenance and lenalidomide in Pv 2.0 - Pv 4.0 was 1:1; between no maintenance, lenalidomide and lenalidomide and vorinostat was 1:1:1 in Pv 5.0; and between no maintenance and lenalidomide was 1:2 in Pv 6.0. It is estimated that this will result in approximately the allocations shown in Table 3.

Table 3: Estimated participant numbers and allocation ratios for comparisons after maintenance

	no treatment	lenalidomide	lenalidomide+ vorinostat	Total
**Pv 2.0 - Pv4.0**	230	230		460
**Pv5.0**	300	300	300	900
**Pv6.0**	280	560		840
**Total**	810	1090	300	2200
**Allocation ratio**	lenalidomide : No treatment		≈ 1.35:1	1900
	lenalidomide±vorinostat : No treatment		≈ 1.72:1	2200
	lenalidomide±vorinostat : lenalidomide		= 1:1	600

The primary comparisons will be lenalidomide versus no treatment and lenalidomide and vorinostat versus lenalidomide. Secondary comparisons will also be undertaken comparing a lenalidomide containing-regimen with no treatment, i.e. lenalidomide±vorinostat and no treatment.

A long-term follow-up analysis comparing lenalidomide versus no treatment will be undertaken when the study is sufficiently mature. This maintenance comparison is powered on demonstrating a 6% increase in 5-year survival in the participants treated with lenalidomide as compared to no treatment only. Expected survival in those not given maintenance is of the order of 50% at 5 years. Under an exponential survival model, to show a 6% increase in 5 year survival in participants treated with lenalidomide would imply a median survival of about 72 months in this group (hazard ratio = 0.84). To demonstrate such an increase, assuming a 2-sided 5% level of significance, 80% power, allowing for a 2% dropout, and with a 3¼-year recruitment and 4-year follow-up period, would require observing 1057 events, with the expected recruitment of 1900 participants and the overall allocation ratio being approximately 1.35:1. 90% power would require 1416 events to be observed.

Intensive pathway

Assuming that approximately 1900 participants reach maintenance and are allocated lenalidomide or no maintenance as shown in Table 3, it is anticipated that 1260 patients would have passed through the intensive pathway in Myeloma XI.

The median progression-free survival for participants receiving no maintenance therapy in a recent trial for transplant-eligible patients was 22 months with an increase in median

survival of 20 months to 42 months in an arm receiving continuous lenalidomide (Palumbo et al., 2014). To demonstrate a slightly smaller increase of 15 months from 22 months in the no maintenance arm to 37 months in the lenalidomide maintenance arm (hazard ratio = 0.60) assuming a 2-sided 5% level of significance, 90% power, allowing for a 2% dropout, and with a 3¼-year recruitment and 4 year follow-up period, would require observing 156 events.

The median survival for participants reaching the maintenance randomisation was approximately 6¾ years in MRC Myeloma IX (= 81 months). This equated to 60% survival at 5 years. An increase in median survival of 2 years to 105 months (hazard ratio = 0.77) is equivalent to a 7½% difference at 5 years from 60% to 67½%. This would require observing 475 events for 80% power which is achievable with 1260 participants in this time frame. 90% power would require 641 events to be observed.

These calculations again assume a 2% dropout, 5% type I error, 3¼ years of recruitment and 4 years of follow-up, with a 1.35:1 ratio of participants in the lenalidomide arm as compared to the no treatment arm.

Non-intensive pathway

Assuming a potential baseline population of 1840 participants in the non-intensive pathway, we should expect, from MRC Myeloma IX figures, that a slightly smaller percentage of non-intensive pathway participants would reach the maintenance randomisation, perhaps 45% rather than 50%. We would, therefore, have approximately 800 participants in this subgroup.

The hazard ratio for patients ineligible for transplant receiving continuous lenalidomide until progression was shown to be 0.72 in the recent FIRST trial (Benbouker et al., 2014) as compared to patients receiving standard therapy. To demonstrate a similar smaller increase of 10 months from 18 months in the no maintenance arm to 28 months in the lenalidomide maintenance arm (hazard ratio = 0.64) assuming a 2-sided 5% level of significance, 90% power, allowing for a 2% dropout, and with a 3¼-year recruitment and 4 year follow-up period, would require observing 215 events, with the recruitment of at least 400 participants.

Again from MRC Myeloma IX the median survival of the non-intensive pathway participants treated with CTDa is approximately 38 months. With 640 participants we would have 80% power to demonstrate a 12 month increase in this median, from 38 to 50 months when 428 events have been observed which again seems achievable in these timelines. 90% power would require 577 events to be observed.

These calculations again assume a 2% dropout, 5% type I error, 3¼ years of recruitment and 4 years of follow-up, with a 1.35:1 ratio of participants in the lenalidomide arm as compared to the no treatment arm.

14.2 PLANNED RECRUITMENT RATE

The study recruited its first patient on 25th May 2010 and 2745 participants have been recruited to the trial under Pv 5.0 and earlier. It is expected that 1651 further participants will be recruited to the study under Pv 6.0 and later with sample size scenarios anticipated to be met by early 2016.

14.3 ANALYSIS TIMELINES

All primary analyses of trial endpoints are described in the Myeloma XI statistical analysis plan. These are related to endpoints that occur during induction and consolidation therapy

(induction chemotherapy, consolidation chemotherapy and high dose therapy with autologous stem cell support) and maintenance therapy.

The timelines for analysis are presented in Table 4. These are subject to change based on updated event prediction rates. Analysis is anticipated to commence in the months described and will take between 3 and 6 months depending on the number of analyses being performed. Further details of analyses are described below.

Table 4: Scheduled analysis for the Myeloma XI study

Intense Data Cleaning	Analysis commences	Analysis block	Trigger Date	Patients included	Analysis	Type	Pathway	Comparison	Endpoint
Jan – Apr 2015	Apr 2015	1	2014-06-01	Pv2.0-Pv5.0	VCD	Interim	Both	VCD vs nothing	PFS
May – Jul 2015	Jul 2015	2	2015-03-01	Pv5.0	Maintenance	Interim	Combined	Len.+ Vor. vs Len.	PFS
May 2015 – Jun 2016	Jul 2016	3	2015-05-01	Pv2.0-Pv6.0+**	Maintenance	Final	Combined	Len. vs nothing	PFS
May 2015 – Jun 2016	Jul 2016	3	2015-12-26	Pv2.0-Pv6.0+	Induction	Final	Intensive	RCD vs CTD	PFS
May 2015 – Jun 2016	Jul 2016	3	2015-12-26	Pv2.0-Pv6.0+	Induction	Final	Non-intensive	RCDa vs CTDa	PFS
May 2015 – Jun 2016	Jul 2016	3	2015-12-26	Pv2.0-Pv6.0+	Induction	Final	Combined	RCD(a) vs CTD(a)	PFS
May 2015 – Jun 2016	Jul 2016	3	2015-12-26	Pv2.0-Pv6.0+	VCD	Final	Both	VCD vs nothing	PFS
May 2015 – Jun 2016	Jul 2016	3	2015-12-26	Pv2.0-Pv6.0+	Maintenance	Final	Intensive	Len. vs nothing	PFS
May 2015 – Jun 2016	Jul 2016	3	2015-12-26	Pv2.0-Pv6.0+	Maintenance	Final	Non-intensive	Len. vs nothing	PFS
May 2015 – Jun 2016	Jul 2016	3	2015-12-26	Pv2.0-Pv6.0+	VCD	Interim	Both	VCD vs nothing	OS
May 2015 – Jun 2016	Jul 2016	3	2015-12-26	Pv2.0-Pv6.0+	Induction	Interim	Intensive	RCD vs CTD	OS
May 2015 – Jun 2016	Jul 2016	3	2015-12-26	Pv2.0-Pv6.0+	Induction	Interim	Non-intensive	RCDa vs CTDa	OS
May 2015 – Jun 2016	Jul 2016	3	2015-12-26	Pv2.0-Pv6.0+	Induction	Interim	Combined	RCD (a) vs CTD(a)	OS
May 2015 – Jun 2016	Jul 2016	3	2016-06-01	Pv2.0-Pv6.0+	Maintenance	Interim	Combined	Len. vs nothing	OS
Jan – Mar 2017	Apr 2017	4	2017-01-01*	Pv6.0+	Induction	Interim	Intensive	CCRD vs. RCD and CTD	PFS
Sept – Dec 2017	Feb 2018	5	2018-01-25	Pv2.0-Pv6.0+	Induction	Final	Intensive	RCD vs CTD	OS
Sept – Dec 2017	Feb 2018	5	2018-01-25	Pv2.0-Pv6.0+	Induction	Final	Non-intensive	RCDa vs CTDa	OS
Sept – Dec 2017	Feb 2018	5	2018-01-25	Pv2.0-Pv6.0+	Induction	Final	Combined	RCD (a) vs CTD(a)	OS
Sept – Dec 2017	Feb 2018	5	2018-01-25	Pv2.0-Pv6.0+	VCD	Final	Both	VCD vs nothing	OS
Jun – Sep 2018	Oct 2018	6	2018-06-30	Pv2.0-Pv6.0+	Maintenance	Final	Combined	Len vs nothing	OS
Jun – Sep 2018	Oct 2018	6	2018-06-30	Pv6.0+	Induction	Interim	Intensive	CCRD vs. RCD and CTD	OS
Apr- Jun 2019	Jul 2019	7	2019-05-01*	Pv6.0+	Induction	Final	Intensive	CCRD vs. RCD and CTD	PFS
Jul – Oct 2019	Nov 2019	8	2019-09-06	Pv2.0-Pv6.0+	Maintenance	Final	Non-intensive	Len vs nothing	OS
Jul – Oct 2019	Nov 2019	8	2019-09-06	Pv2.0-Pv6.0+	Maintenance	Final	Intensive	Len vs nothing	OS
Sept – Dec 2019	Jan 2020	9	2019-12-31	Pv5.0	Maintenance	Final	Combined	Len.+ Vor. vs Len.	PFS
Sept – Dec 2019	Jan 2020	9	2019-12-31	Pv5.0	Maintenance	Final	Combined	Len.+ Vor. vs Len.	OS
Sept – Dec 2019	Jan 2020	9	2019-12-31*	Pv6.0+	Induction	Final	Intensive	CCRD vs. RCD and CTD	OS
Sept – Dec 2019	Jan 2020	9	2019-12-31	Pv2.0-Pv6.0+	Maintenance	Final	Combined	Len vs nothing	OS

**Estimates based on patients entered under Pv5.0.*

***Pv6.0+ refers to protocol version 6.0 and any subsequent amendments.*

14.3.1 Induction and consolidation therapy

Participants entered into the trial will be analysed at event-driven timepoints post-induction randomisation:

1. An interim analysis of response upgrade and PFS comparing VCD and no treatment took place when 400 participants had completed VCD treatment (occurred in April 2015).
2. A final analysis of PFS comparing CTD and RCD and CTDa and RCDa when all event triggers have been passed (expected to occur in July 2016). A final analysis of PFS comparing VCD and no treatment will also be undertaken at this time point.
3. An interim analysis of OS comparing CTD and RCD and CTDa and RCDa when half the required number of events has been observed in all comparisons (expected to occur in July 2016). The O'Brien and Fleming alpha spending function will be used to account for this interim analysis in final analysis (See Section 15.2).
4. An interim analysis of OS comparing VCD and no treatment when half the required number of events has been observed (expected to occur in July 2016). The O'Brien and Fleming alpha spending function will be used to account for this interim analysis in final analysis (See Section 15.2).
5. An interim analysis of PFS comparing CCRD and RCD and CTD when half the required number of events has been observed (expected to occur in April 2017). The O'Brien and Fleming alpha spending function will be used to account for this interim analysis in final analysis (See Section 15.2).
6. A final analysis of OS comparing CTD and RCD and CTDa and RCDa when all event triggers have been passed (expected to occur in February 2018). A final analysis of OS comparing VCD and no treatment will also be undertaken at this time point.
7. An interim analysis of OS comparing CCRD and RCD and CTD when half the required number of events has been observed (expected to occur in April 2017). The O'Brien and Fleming alpha spending function will be used to account for this interim analysis in final analysis (See Section 15.2).
8. A final analysis of PFS comparing CCRD and RCD and CTD when all event triggers have been passed (expected to occur in July 2019).
9. A final analysis of OS comparing CCRD and RCD and CTD when all event triggers have been passed (expected to occur in January 2020).

14.3.2 Maintenance therapy

Participants entered into the trial will be analysed at event-driven timepoints post-maintenance randomisation:

1. An interim analysis to assess the vorinostat and lenalidomide arm for harm as compared to lenalidomide alone. This will be undertaken when 130 PFS events have been observed in the vorinostat and lenalidomide and lenalidomide arms in participants randomised under Pv 5.0 (expected to occur in July 2015).
2. A final analysis to compare PFS in lenalidomide and no maintenance arms when all event triggers have been passed (expected to occur at the end of July 2016).
3. A final analysis to compare PFS in lenalidomide and no maintenance arms in each trial pathway (intensive and non-intensive) when all event triggers have been passed (expected to occur at the end of July 2016).
4. An interim analysis to compare OS in lenalidomide and no maintenance arms when half the required number of events has been observed (expected to occur at the end of July 2016). The O'Brien and Fleming alpha spending function will be used to account for this interim analysis in final analysis (See Section 15.2).

5. A final analysis to compare OS in lenalidomide and no maintenance arms when all event triggers have been passed (expected to occur in October 2018).
6. A final analysis to compare OS in lenalidomide and no maintenance arms in each trial pathway (intensive and non-intensive) when all event triggers have been passed (expected to occur in October 2018).
7. A final analysis to compare PFS in vorinostat and lenalidomide and lenalidomide arms when all event triggers have been passed (expected to occur in November 2019).
8. A final analysis to compare OS in vorinostat and lenalidomide and lenalidomide arms when all event triggers have been passed (expected to occur in November 2019).
9. A further updated final analysis of patients in long-term follow-up to compare OS in lenalidomide and no maintenance arms when all event triggers have been passed (expected to occur in January 2020).

Apart from these planned analyses, no other formal analyses of the Myeloma XI study are planned. Secondary and exploratory analysis will be undertaken at the event-driven timepoints described above, as appropriate.

Analyses of biological objectives will be undertaken at the discretion of the trial management group. Any release of trial endpoint data will be authorised by the Chair of the Myeloma XI Data Monitoring and Ethics Committee and will be blinded to randomised treatment allocation until analysis of main trial comparisons has been completed and submitted for publication.

15. STATISTICAL ANALYSIS

15.1 GENERAL CONSIDERATIONS

Statistical analysis is the responsibility of the CTRU statisticians. A full statistical analysis plan will be written before any analyses are undertaken. The analysis plan will be written in accordance with current CTRU standard operating procedures and will be finalised and agreed by the following people: the trial statistician and supervising statistician, the Chief Investigator, the CTRU Scientific and Delivery Leads and the Senior Data Manager. Any changes to the finalised analysis plan, and reasons for changes, will be documented.

All analyses will be conducted on the intention-to-treat (ITT) population, where participants will be included according to the treatment they were randomised to regardless of eligibility, whether they prematurely discontinued treatment or did not comply with the regimen. Separate ITT populations will be defined for the induction, consolidation and maintenance parts of the trial. The ITT population for the comparison of lenalidomide with no maintenance will consist of all participants randomised to lenalidomide or no maintenance under Pv 1.0–Pv 6.0 (regardless of the change of dose from 25 mg under Pv1.0–Pv 4.0 to 10 mg under Pv5.0). For the comparison of lenalidomide and vorinostat (RZ) with no maintenance, the population will only consist of participants entered into the trial prior to Pv 6.0 (RZ was introduced in Pv 5.0). For the comparison of RZ with lenalidomide, the population will consist of participants randomised to RZ or lenalidomide under Pv 5.0. In all three cases, participants will be included according to the treatment they were randomised to regardless of eligibility, whether they prematurely discontinued the treatment or did not comply with the regimen.

A per-protocol analysis, where participants will be included according to the treatment they received, will be considered for the primary endpoints if there are a considerable number of protocol violators. The safety population will consist of all participants who receive at least one dose of the relevant study treatment.

With the exception of the analysis of induction chemotherapy, the intensive and non-intensive trial pathways will be combined for the primary comparisons, although descriptive statistics will be presented for each of the randomised groups within the trial pathways. All analyses will be adjusted for minimisation factors excluding centre.

15.2 FORMAL INTERIM ANALYSES

Interim statistical summaries will be presented to the Data Monitoring and Ethics Committee in strict confidence at approximately yearly intervals. Formal interim analyses will be undertaken at the time points described in Section 14.3 and summarised in Table 4. Interim analyses will generally be undertaken for overall survival comparisons when half of the events have been observed, unless otherwise stated. Interim analyses will not be undertaken for progression-free survival comparisons due to the rolling nature of the study, unless otherwise stated. An overall two-sided 5% significance level will be used for all efficacy endpoint comparisons. For the primary endpoints, this will be adjusted to account for the planned interim analysis. The O'Brien and Fleming alpha spending function will be used, which suggests an alpha level of 0.047 for the final analysis and 0.005 for the interim analysis.

This committee, in the light of the interim data, and any advice or evidence they wish to request, will advise the Trial Steering Committee if there is proof beyond reasonable doubt that one treatment is better and recommend appropriate changes to the trial protocol.

15.3 PRIMARY ENDPOINT ANALYSES

The intensive and non-intensive trial pathways will not be combined for the primary comparisons of RCD with CTD. To compare RCD with CTD, CCRD with RCD/CTD combined or with whichever of these two is superior, and lenalidomide, lenalidomide and vorinostat with no maintenance, Cox regression analyses will be used to analyse overall and progression-free survival accounting the minimisation factors excluding centre. Overall and progression-free survival curves will be calculated using the Kaplan Meier method, and hazard ratios and corresponding 95% confidence intervals will be calculated. Note that although the significance level has been slightly reduced to account for the interim analyses, confidence intervals will still be presented at the 95% level as these are for summary purposes.

Although the two pathways are distinct, the induction chemotherapy result being different across the two pathways will be tested for, using a meta-analytic approach testing for heterogeneity between the two pathways. The only likely confounding variable is age and that will be examined using a Cox regression analysis. If they both show a similar effect of lenalidomide, the effects will be combined via a stratified analysis and a meta-analysis.

A new statistical method has been developed to enable survival curves and associated log rank tests for the primary RCD vs CTD comparison to be produced, adjusting for possible confounding effects, should there be a higher response rate in one arm, and a significant VCD effect which could ‘rescue’ non-responders and so confound the primary comparison (manuscript in preparation for submission to Statistics in Medicine).

15.4 SECONDARY ENDPOINT ANALYSES

The intensive and non-intensive trial pathways will not be combined for the primary comparisons of RCD with CTD and CCRD with RCD/CTD combined. Responses to the randomised treatments will be summarised by treatment group (ITT population) and 95% confidence intervals will be calculated. Treatment groups will be compared with respect to the proportion achieving remission (very good partial or complete) using logistic regression to adjust for the minimisation factors excluding centre (ITT population).

Conversion rates to CR/VGPR for participants who undergo bortezomib-cyclophosphamide-dexamethasone randomisation will be summarised by treatment group (ITT population) and 95% confidence intervals will be calculated.

Safety analyses will summarise the adverse event rates and serious adverse events separately for each of the treatments. Safety data will be presented for participants receiving any of the relevant study treatment by treatment group and relationship to study treatment.

15.5 SUBGROUP ANALYSES

Cytogenetic subgroups will be analysed to explore a number of specific hypotheses, including the effect on OS, PFS and response. Some examples of what will be studied include chromosome 14 translocations and abnormalities of chromosome 1p, 1q, 13q and 17p. In addition, other regions considered to be of interest will be analysed according to the statistical analysis plan. Other subgroup analyses may also be carried out and will be described in the Myeloma XI statistical analysis plan.

Subgroup analyses may, by chance, generate false negative or positive results. Those carried out will be interpreted with caution.

16. DATA MONITORING

16.1 DATA MONITORING AND ETHICS COMMITTEE

An independent Data Monitoring and Ethics Committee (DMEC) will be established to review the safety and ethics of the trial.

Detailed unblinded reports will be prepared by the CTRU for the DMEC at approximately yearly intervals and the committee will be required to review any formal interim analysis reports as detailed in Section 15. The DMEC will also review cumulative unblinded safety data along with individual SAE/SAR listings every 3 months.

16.2 DATA MONITORING

Data will be monitored for quality and completeness by the CTRU. Missing data will be chased until it is received, confirmed as not available, or the trial is at analysis. The CTRU/Sponsor will reserve the right to intermittently conduct source data verification exercises on a sample of participants, which will be carried out by staff from the CTRU/Sponsor. Source data verification will involve direct access to participants notes at the participating hospital sites and the ongoing central collection of copies of consent forms and other relevant investigation reports. A Trial Monitoring Plan will be developed and agreed by the Trial Management Group.

16.3 CLINICAL GOVERNANCE ISSUES

To ensure responsibility and accountability for the overall quality of care received by participants during the study period, clinical governance issues pertaining to all aspects of routine management will be brought to the attention of the TSC and, where applicable, to individual NHS Trusts.

17. QUALITY ASSURANCE, SPONSORSHIP, ETHICAL CONSIDERATIONS, CONFIDENTIALITY AND STATEMENT OF INDEMNITY

17.1 QUALITY ASSURANCE

The trial will be conducted in accordance with the principles of Good Clinical Practice in clinical trials, as applicable under UK regulations, the NHS Research Governance Framework (and Scottish Executive Health Department Research Governance Framework for Health and Social Care 2006, and through adherence to CTRU Standard Operating Procedures (SOPs).

CTRU and Sponsor have systems in place to ensure that serious breaches of GCP or the trial protocol are picked up and reported. Investigators are required to promptly notify the CTRU of a serious breach (as defined in Regulation 29A of the Medicines for Human Use (Clinical Trials) Regulations 2004 [Statutory Instrument 2004/1031], as amended by Statutory Instrument 2006/1928) that they become aware of. A ‘serious breach’ is a breach which is likely to affect to a significant degree:

i. The safety or physical or mental integrity of the subjects of the trial; or
ii. The scientific value of the trial.

For further information, the Investigator should contact the Senior Trial Co-ordinator at the CTRU.

17.2 SPONSORSHIP

The sponsor for this trial is The University of Leeds.

17.3 ETHICAL CONSIDERATIONS

The trial will be performed in accordance with the recommendations guiding clinicians in biomedical research involving human subjects adopted by the 18th World Medical Assembly, Helsinki, Finland, 1964, amended at the 48th World Medical Association General Assembly, Somerset West, Republic of South Africa, October 1996. Informed written consent will be obtained from the patients prior to randomisation into the study. The right of a patient to refuse participation without giving reasons must be respected. The participants must remain free to withdraw at any time from the study without giving reasons and without prejudicing his/her further treatment. The study will be submitted to and approved by a main Research Ethics Committee (REC) and the appropriate Site Specific Assessor for each participating centre, prior to entering participants into the study. The CTRU will provide the main REC with a copy of the final protocol, patient information sheets and consent forms, and all other relevant study documentation.

17.4 CONFIDENTIALITY

All information collected during the course of the trial will be kept strictly confidential. Information will be held securely on paper and electronically at the Clinical Trials Research Unit (CTRU). The CTRU will comply with all aspects of the 1998 Data Protection Act and operationally this will include:

- Consent from participants to record personal details including name, date of birth, NHS number, hospital number.
- Appropriate storage, restricted access and disposal arrangements for participant personal and clinical details.
- Consent from participants for access to their medical records by responsible individuals from the research staff or from regulatory authorities, where it is relevant to trial participation.
- Consent from participants for the data collected for the trial to be used to evaluate safety and develop new research.
- Participant name will be collected when a participant is randomised into the trial but all other data collection forms that are transferred to or from the CTRU will be coded with a trial number and will include two additional participant identifiers, usually the participant's initials and date of birth.
- Where central monitoring of source documents by CTRU (or copies of source documents) is required (such as scans or local blood results), the participant's name must be obliterated by site before sending.
- Samples sent to central laboratories will require participant names to be included on the samples in order for the samples to be correctly identified and processed upon receipt in line with the minimal identifiable information required by the laboratories. Results can then be reported back to the treating physician. This is clarified within the patient information sheet.
- Where anonymisation of documentation is required, sites are responsible for ensuring only the instructed identifiers are present before sending to CTRU.

If a participant withdraws consent from further trial treatment and / or further collection of data their existing samples and data will remain on file and will be included in the final study analysis.

17.5 ARCHIVING

At the end of the trial, data will be securely archived in line with the Sponsor's procedures for a minimum of 15 years. Data held by the CTRU will be archived in the Leeds Sponsor archive facility and site data and documents will be archived at the participating centres. Following authorisation from the Sponsor, arrangements for confidential destruction will then be made.

17.6 STATEMENT OF INDEMNITY

This trial is sponsored by the University of Leeds and the University of Leeds will be liable, in certain circumstances, for harm caused by participation in the trial. The NHS has a duty of care to patients treated, whether or not the patient is taking part in a clinical trial, and the NHS remains liable for clinical negligence and other negligent harm to patients under this duty of care.

18. STUDY ORGANISATIONAL STRUCTURE

18.1 RESPONSIBILITIES

Chief Investigator
The Chief Investigator will have overall responsibility for the design and set-up of the trial, the investigational drug supply and pharmacovigilance within the trial.

Clinical Trials Research Unit
The CTRU will have responsibility for conduct of the trial in accordance with relevant GCP standards and CTRU SOPs.

18.2 OPERATIONAL STRUCTURE

Chief Investigator
The Chief Investigator is involved in the design, conduct, co-ordination and management of the trial.

Trial Management Group
The TMG, comprising the Chief Investigator, CTRU team, and other key external member of staff involved in the trial will be assigned responsibility for the clinical set-up, ongoing management, promotion of the trial, and for the interpretation of results. Specifically the TMG will be responsible for i) protocol completion, ii) CRF development, iii) obtaining approval from the main REC and supporting applications for Site Specific Assessments, iv) submitting a CTA application and obtaining approval from the MHRA, v) completing cost estimates and project initiation, vi) nominating members and facilitating the TSC and DMEC, vii) reporting of serious adverse events, viii) monitoring of screening, recruitment, treatment and follow-up procedures, ix) auditing consent procedures, data collection, trial end-point validation and database development.

Clinical Trials Research Unit
The CTRU will provide set-up and monitoring of trial conduct to CTRU SOPs and the GCP Conditions and Principles as detailed in the UK Medicines for Human Use (Clinical Trials) Regulations 2006, including randomisation design and service, database development and provision, protocol development, CRF design, trial design, source data verification, monitoring schedule and statistical analysis for the trial. In addition, the CTRU will support the main REC, Site Specific Assessment and R&D submissions and clinical set-up, ongoing management including training, monitoring reports and promotion of the trial. The CTRU will be responsible for the day-to-day running of the trial including trial administration, database administrative functions, data management, safety reporting and all statistical analyses.

Data Monitoring and Ethics Committee
The DMEC will review the safety and ethics of the trial by reviewing interim data during recruitment. The Committee will meet annually as a minimum.

Trial Steering Committee
The TSC, with an independent Chair, will provide overall supervision of the trial, in particular trial progress, adherence to protocol, participant safety and consideration of new information. It will include an Independent Chair, not less than two other independent members and a consumer representative. The Chief Investigator and other members of the TMG may attend the TSC meetings and present and report progress. The Committee will meet annually as a minimum.

19. PUBLICATION POLICY

The trial will be registered with an authorised registry, according to the ICMJE Guidelines, prior to the start of recruitment.

The success of the trial depends upon the collaboration of all participants. For this reason, credit for the main results will be given to those who have collaborated in the trial, through authorship and contributorship. Uniform requirements for authorship for manuscripts submitted to medical journals will guide authorship decisions. These state that authorship credit should be based only on substantial contribution to:

- Conception and design, or acquisition of data, or analysis and interpretation of data;
- Drafting the article or revising it critically for important intellectual content;
- And final approval of the version to be published;
- And that all these conditions must be met (www.icmje.org).

In light of this, the Chief Investigator and relevant senior CTRU staff will be named as authors in any publication. In addition, all collaborators will be listed as contributors for the main trial publication, giving details of roles in planning, conducting and reporting the trial.

To maintain the scientific integrity of the trial, data will not be released prior to the end of the trial, either for trial publication or oral presentation purposes, without the permission of the Data Monitoring and Ethics Committee or Trial Steering Committee. For bolt-on/laboratory studies associated with this protocol, data release will be governed by a separate data release agreement. In addition, collaborators must not publish data concerning their patients which is directly relevant to the questions posed in the trial until the first publication of the analysis of the primary endpoint. Manuscripts will be submitted to a high impact factor internationally recognised journal e.g. Blood, Journal of Clinical Oncology. All publications (abstracts and full manuscripts) must be reviewed by the Trial Management Group.

20. KEY REFERENCES

Myeloma treatment

Attal M, Harousseau JL, Stoppa AM, et al. (1996). A prospective randomised trial of autologous bone marrow transplantation versus conventional chemotherapy in multiple myeloma. Intergroupe Français du Myélome. *N Eng J Med:* 335: 91-7.

Barlogie B, Jagannath S, Desikan KR, et al. (1999). Total therapy with tandem transplants for newly diagnosed multiple myeloma. *Blood*: 93: 55-65.

Blade J, Samson D, Reece D, et al. (1998). Criteria for evaluating disease response and progression in patients with multiple myeloma treated by high-dose therapy and haemopoietic stem cell transplantation. Myeloma Subcommittee of the EBMT. *Br J Haematol*: 102: 1115-23.

Blade J, Sureda A, Ribera JM, et al. (2001). High-dose therapy autotransplantation/intensification vs continued conventional chemotherapy in multiple myeloma patients responding to initial treatment chemotherapy. Results of a prospective randomised trial from the Spanish Cooperative Group PETHEMA. *Blood*: 98: abstr 3386.

Cavo M. Tosi P, Zamagni E, et al. The Bologna 96 clinical trial of single vs double PBSCT transplantation for previously untreated MM: results of an interim analysis. Proceedings of the VIII International Myeloma Workshop.

Child JA, Morgan GJ, Davies FE, Owen RG, Bell SE, Hawkins K, Brown J, Drayson MT, Selby PS, for the Medical Research Council Adult Leukaemia Working Party. (2003). High-dose chemotherapy with hematopoietic stem-cell rescue for multiple myeloma. *N Engl J Med*: 348: 1875-1883.

Cuzick J, Cooper EH, MacLennan IC. (1985). The prognostic value of serum beta 2 microglobulin compared with other presentation features in myelomatosis. *Br J Cancer*: Jul; 52: 1-6.

Davies FE, Forsyth PD, Rawstron AC, et al. (2001). The impact of attaining a minimal disease state following high-dose melphalan and autologous transplantation for multiple myeloma. *Br J Haematol:* 112: 814-9.

Durie BG, Harousseau JL, Miguel JS, et al. (2006). International uniform response criteria for multiple myeloma. *Leukemia:* 20:1467- 73.

Facon T, Avet-Loiseau H, Guillerm G, et al. (2001). Chromosome 13 abnormalities identified by FISH analysis and serum beta2-microglobulin produce a powerful myeloma staging system for patients receiving high-dose therapy. *Blood*: 1; 97: 1566-71.

Fermand JP, Marolleau JP, Alberti C, et al. (2001). Single versus tandem high-dose therapy supported with autologous blood stem cell transplantation using unselected or CD34 enriched ABSC: preliminary results of a two by two designed randomized trial in 230 young patients with multiple myeloma. *Blood*: 1; 98: abstr 3387.

Fermand JP, Ravaud P, Chevret S, C, et al. (1998). High-dose therapy and autologous blood stem cell transplantation in multiple myeloma: up front or rescue treatment? Results of a multicenter sequential randomised clinical trial. *Blood:* 92: 3131-6.

Fermand JP, Ravaud P, Katsahian S, et al. (1999). High-dose therapy and autologous blood stem cell transplantation versus conventional treatment in multiple myeloma: results of a randomised trial in 190 patients 55-65 years of age. *Blood*: 94: abstr 1754.

Gore ME, Selby PJ, Viner C, et al. (1989). Intensive treatment of multiple myeloma and criteria for complete remission. *Lancet*: 2: 879-82.

Greipp PR, San Miguel J, Durie BGM, Crowley JJ, Barlogie B, Blade J, Boccadoro M, Child JA, Avet-Loiseau H, Kyle RA, Lahuerta JJ, Ludwig H, Morgan G, Powles R, Shimizu K, Shustik C, Sonneveld P, Tosi P, Turesson I, Westin J. (2005). International Staging System for Multiple Myeloma. *JCO:* 23: 3412-20.

Lahuerta JJ, Martinez-Lopez J, Serna JD, et al. (2000). Remission status defined by immunofixation vs. electrophoresis after autologous transplantation has a major impact on the outcome of multiple myeloma. *Br J Haemat:* 109: 438-46.

Lenhoff S, Hjorth M, Holmberg E, et al. (2000). Impact on survival of high-dose therapy with autologous stem cell support in patients younger than 60 years with newly diagnosed multiple myeloma: a population based-study. *Blood:* 95: 7-11.

MacLennan IC, Chapman C, Dunn J, Kelly K. (1992). Combined chemotherapy with ABCM versus melphalan for treatment of myelomatosis. The Medical Research Council Working Party for Leukaemia in Adults. *Lancet*: 25: 200-5.

McElwain TJ, Powles RL. (1983). High dose intravenous melphalan for plasma-cell leukaemia and myeloma. *Lancet:* ii: 822-4.

Rajkumar VS, Harousseau JL, Durie B, et al (2011). Consensus recommendations for the uniform reporting of clinical trials: report of the International Myeloma Workshop Consensus Panel 1. *Blood*: 117(18): 4691-5

Samson D, Gaminara E, Newland A, et al. (1989). Infusion of vincristine and doxorubicin with oral dexamethasone as first-line therapy for multiple myeloma. *Lancet:* 2: 882-5.

Segeren CM, Sonneveld P, Van der Holt B, et al. (2001). Myeloablative treatment following intensified chemotherapy in untreated multiple myeloma: A prospective, randomized phase III study. Proceedings of the American Society of Haematology 43rd Annual Meeting.

Simnett SJ, Stewart LA, Sweetenham J, Morgan G, Johnson PW. (2000). Autologous stem cell transplantation for malignancy: a systematic review of the literature. *Clin Lab Haematol:* 22:61-72.

A Report of the International Myeloma Working Group. (2003). Criteria for the classification of monoclonal gammopathies, multiple myeloma, and related disorders. *Br J Haematol*: 121:749-757.

Thalidomide

Attal M, Harousseau JL, Leyvraz S, et al. (2006). Maintenance therapy with thalidomide improves survival in patients with multiple myeloma. *Blood*: 108: 3289-94.

Barlogie B, Desikan R, Eddlemon P, Spencer T, Zeldis J, Munshi N, Badros A, Zangari M, Anaissie E, Epstein J, Shaughnessy J, Ayers D, Spoon D, Tricot G. (2001). Extended survival in advanced and refractory multiple myeloma after single-agent thalidomide: identification of prognostic factors in a phase 2 study of 169 patients. *Blood:* 98: 492-4.

Cavo M, Zamagni E, Tosi P, et al. (2005). Superiority of thalidomide and dexamethasone over vincristine-doxorubicindexamethasone (VAD) as primary therapy in preparation for autologous transplantation for multiple myeloma. *Blood*: 106: 35-39.

Cavenagh JD, Oakervee H. (2003). Thalidomide in multiple myeloma: current status and future prospects. *Br J Haematol:* 120: 18-26.

Davies FE, Raje N, Hideshima T, Lentzsch S, Young G, Tai YT, Lin B, Podar K, Gupta D, Chauhan D, Treon SP, Richardson PG, Schlossman RL, Morgan GJ, Muller GW, Stirling DI, Anderson KC. (2001). Thalidomide and immunomodulatory derivatives augment natural killer cell cytotoxicity in multiple myeloma. *Blood:* 98: 3495-6.

Kyriakou C, Thomson K, D'Sa S, Flory A, Hanslip J, Goldstone AH, et al. (2005). Low-dose thalidomide in combination with oral weekly cyclophosphamide and pulsed dexamethasone is a well tolerated and effective regimen in patients with relapsed and refractory multiple myeloma. *Br J Haematol:* 129:763-770.

Facon T, Mary JY, Hulin C, et al. (2007). Melphalan and prednisone plus thalidomide versus melphalan and prednisone alone or reduced-intensity autologous stem cell transplantation in elderly patients with multiple myeloma (IFM 99-06): a randomised trial. *Lancet*: 370:1209-18.

Garcia-Sanz R, Gonzalez-Fraile MI, Sierra M, Lopez C, Gonzalez M, San Miguel JF. (2002). The combination of thalidomide, cyclophosphamide and dexamethasone (ThaCyDex) is feasible and can be an option for relapsed/refractory multiple myeloma. *Hematol J:* 3: 43-8.

Morgan GJ, Davies FE, Owen RG, Rawstron AC, Bell S, Cocks K, Gregory W, Jackson G, Drayson MT, Jenner MW, Child JA. (2007). Thalidomide Combinations Improve Response Rates; Results from the MRC IX Study. *Blood*: (ASH Annual Meeting Abstracts), Nov; 110: 3593.

Hideshima T, Chauhan D, Shima Y, Raje N, Davies FE, Tai YT, Treon SP, Lin B, Schlossman RL, Richardson P, Muller G, Stirling DI, Anderson KC. (2000). Thalidomide and its analogs overcome drug resistance of human multiple myeloma cells to conventional therapy. *Blood*: 96: 2943-50.

Neben K, Mytilineos J, Moehler TM, Preiss A, Kraemer A, Ho AD, Opelz G, Goldschmidt H. (2002). Polymorphisms of the tumor necrosis factor-alpha gene promoter predict for outcome after thalidomide therapy in relapsed and refractory multiple myeloma. *Blood*: 100: 2263-65.

Osman K, Comenzo R, Rajkumar SV. (1999). Deep venous thrombosis and thalidomide therapy for multiple myeloma. *N Engl J Med:* 344: 1951-2.

Palumbo A, Bringhen S, Caravita T, et al. (2006). Oral melphalan and prednisone chemotherapy plus thalidomide compared with melphalan and prednisone alone in elderly patients with multiple myeloma: randomised controlled trial. *Lancet*: 367:825-31.

Rajkumar SV, Hayman, SR, Gertz MA, Dispenzieri A, Lacy M, Greipp PR, Geyer S, Itturia N, Fonesca R, Lust JA, Kyle R, Witzig TE. (2001). Combination therapy with thalidomide plus dexamethasone (THAL/DEX) for newly diagnosed myeloma (MM). *Blood*: 98; Abst 3525.

Rajkumar SV, Hayman S, Gertz MA, Dispenzieri A, Lacy MQ, Greipp PR, Geyer S, Iturria N, Fonseca R, Lust JA, Kyle RA, Witzig TE. (2002). Combination therapy with thalidomide plus dexamethasone for newly diagnosed myeloma. *J Clin Oncol:* 20: 4319-23.

Rajkuma SV, Blood E, Vesole D, Fonseca R, Greipp PR; (2006). Eastern Cooperative Oncology Group. Phase III clinical trial of thalidomide plus dexamethasone compared with dexamethasone alone in newly diagnosed multiple myeloma: a clinical trial coordinated by the Eastern Cooperative Oncology Group. *J Clin Oncol*: 24: 431-436.

Sidra GM, Byrne JL, Myers, Mitchell DC, Russell NH. (2002). Combination therapy with thalidomide, cyclophosphamide and dexamethasone (C-ThaD) for relapsed and primary refractory multiple myeloma. *Br J Haematol:* 117:49-93.

Singhal S, Mehta J, Desikan R, Ayers D, Roberson P, Eddlemon P, et al. (1999). Antitumor activity of thalidomide in refractory multiple myeloma. *N Engl J of Med:* 341:1565-71.

Wu P, Davies FE, Horton C, et al. (2006). The combination of cyclophosphomide, thalidomide and dexamethasone is an effective alternative to CVAMP as induction chemotherapy prior to autologous transplantation for multiple myeloma – a case matched analysis. *Leuk Lymphoma*: 47:2335-8.

Yakoub-Agha I, Attal M, Dumontet C, Delannoy V, Moreau P, Berthou C, Lamy T, Grosbois B, Dauriac C, Dorvaux V, Bay JO, Monconduit M, Harousseau JL, Duguet C, Duhamel A, Facon T. (2002). Thalidomide in patients with advanced multiple myeloma: a study of 83 patients - report of the intergroupe francophone du myelome (IFM). *Hematol J:* 3:185-92.

Revlimid / Lenalidomide

Bartlett JB, Michael A, Clarke IA, Dredge K, Nicholson S, et al. (2004). Phase I study to determine the safety, tolerability and immunostimulatory activity of thalidomide analog lenalidomide in patients with metastatic malignant melanoma and other advanced cancers. *British J of Cancer:* 90, 955-961.

Corral LF, Haslett PAJ, Muller FW, Chen R, Wong LM, Ocampo CJ, Patterson RT, Stirling DI, Kaplan G. (1993). Differential cytokine modulation and T cell activation by two distinct classes of thalidomide analogues that are potent inhibitors of TNF-alpha. *J Immunol*: 163:380-386.

Revlimid™ (lenalidomide) healthcare professional information pack for UK. Celgene Corporation.

Dimopoulos M.A., Spencer A., Attal M., Prince M., Harousseau J., Dmoszynska A., San Miguel J, Hellmann A, Facon T, Foà R, Corso A, Masliak Z, Olesnyckyj M, Yu Z, Patin J, Zeldis JB, Knight RD. (2007). Multiple Myeloma (010) Study Investigators. Lenalidomide plus dexamethasone for relapsed or refractory multiple myeloma. *N Engl J Med:* 357:2123-32.

Dredge K, Horsfall R, Robinson S, Zhang L-H, Lu L, et al. (2005). Orally administered lenalidomide is anti-angiogenic in vivo and inhibits endothelial cell migration and Akt phosphorylation in vitro. *Microvascular Research:* 69 56-63.

Karam MA, Choueiri TK, Jawde RA, et al. (2006). Lenalidomide and pegylated liposomal doxorubicin-based chemotherapy for relapsed or refractory multiple myeloma: safety and efficacy. *Ann Oncol*: 17:1766-71.

List A, Kurtin S, Roe DJ, Buresh A, Mahadevan D, et al. (2005). Efficacy of lenalidomide in myelodysplastic syndromes. *N Engl J Med:* 352:549-57.

List AF, Dewald G, Bennett J, Giagounadis A, Raza A, et al. (2005). Hematologic and cytogenetic (CTG) response to lenalidomide in patients with transfusion-independent (TD) myelodysplastic syndrome (MDS) and chromosome 5q31.1 deletion: Results of the multicenter MDS-003 study. *Proc ASCO:* abstract #5.

Liu Y, Tohnya TM, Figg WD, Gulley JL, Arlen PM, et al. (2003). Phase I study of lenalidomide (Revimid), a thalidomide derivative, in patients with refractory metastatic cancer. *Proc ASCO:* Abstract #927.

Morgan GJ, Schey SA, Wu P, Srikanth M, Phekoo KJ, Jenner M, Davies FE. (2007). Lenalidomide (Revlimid), in combination with cyclophosphamide and dexamethasone (RCD), is an effective and tolerated regimen for myeloma patients. *BJH:* 137: 268-9.

Palumbo A, Falco P, Corradini P, et al. (2007). Melphalan, prednisone, and lenalidomide treatment for newly diagnosed myeloma: a report from the GIMEMA--Italian Multiple Myeloma Network. *J Clin Oncol*: 25:4459-65.

Rajkumar S.V., Hayman S.R., Lacy M.Q., Dispenzieri A., Geyer S.M., Kabat B., Zeldenrust S.R., Kumar S., Greipp P.R., Fonseca R., Lust J.A., Russell S.J., Kyle R.A., Witzig T.E., Gertz M.A. (2005). Combination therapy with lenalidomide plus dexamethasone (Rev/Dex) for newly diagnosed myeloma. *Blood*: 106:4050−4053.

Rajkumar S.V., Jacobus S, Callander N, Fonseca R, Vesole D, Williams M, Abonour R, Siegel D, Greipp P. (2007). A Randomized Trial of Lenalidomide Plus High-Dose Dexamethasone (RD) Versus Lenalidomide Plus Low-Dose Dexamethasone (Rd) in Newly Diagnosed Multiple Myeloma (E4A03): A Trial Coordinated by the Eastern Cooperative Oncology Group. *Blood:* (ASH Annual Meeting Abstracts), Nov; 110: 74.

Richardson P.G., Schlossman R.L., Weller E., Hideshima T., Mitsiades C., Davies F., LeBlanc R., Catley L.P., Doss D., Kelly K., McKenney M., Mechlowicz J., Freeman A., Deocampo R., Rich R., Ryoo J.J., Chauhan D., Balinski K., Zeldis J., Anderson K.C. (2002). Immunomodulatory drug CC-5013 overcomes drug resistance and is well tolerated in patients with relapsed multiple myeloma. *Blood:* 100, 3063−3067.

Richardson P.G., Blood E., Mitsiades C.S., Jagannath S., Zeldenrust S.R., Alsina M., Schlossman R.L., Rajkumar S.V., Desikan K.R., Hideshima T., Munshi N.C., Kelly-Colson K., Doss D., McKenney M.L., Gorelik S., Warren D., Freeman A., Rich R., Wu A., Olesnyckyj M., Wride K., Dalton W.S., Zeldis J., Knight R., Weller E., Anderson K.C. (2006). A randomized phase 2 study of lenalidomide therapy for patients with relapsed or relapsed and refractory multiple myeloma. *Blood*: 108, 3458−3464.

Schafer PH, Gandhi AK, Loveland MA, Chen RS, Man H-W, et al. (2003). Enhancement of cytokine production and AP-1 transcriptional activity in T cells by thalidomide-related immunomodulatory drugs. *J of Pharmacology and Exp Therapeutics:* 305:1222- 1232.

Weber D.M., Chen C., Niesvizky R., Wang M, Belch A, Stadtmauer EA, Siegel D, Borrello I, Rajkumar SV, Chanan-Khan AA, Lonial S, Yu Z, Patin J, Olesnyckyj M, Zeldis JB, Knight RD, Multiple Myeloma (009) Study Investigators. (2007). Lenalidomide plus dexamethasone for relapsed multiple myeloma in North America. *N Engl J Med*: 357:2133-42.

Wu A, Scheffler MR. (2004). Multiple-dose pharmacokinetics and safety of lenalidomide in 15 multiple myeloma patients. *Proc ASCO:* Abstract #2056.

Zangari M, Tricot G, Zeldis J, Eddlemon P, Saghafifar F, Barlogie B. (2001). Results of phase I study of lenalidomide for the treatment of multiple myeloma (MM) patients who relapse after high dose chemotherapy (HDCT). *Blood:* 98:775a (Abstract #3226).

Attal M, Lauwers V, Marit G et al. (2010). Maintenance Treatment with Lenalidomide After Transplantation for Myeloma: Final Analysis of the IFM 2005-02. ASH Annual Meeting Abstracts, *Blood* 116 (21): 310

McCarthy PL, Owzar K, Anderson K et al. (2010). Phase III Intergroup Study of Lenalidomide Versus Placebo Maintenance Therapy Following Single Autologous Hematopoietic Stem Cell Transplantation (AHSCT) for Multiple Myeloma: CALGB 100104. ASH Annual Meeting Abstracts, *Blood* 2010 116: 37

Palumbo A, Dimopoulos M, Delforge M, Hajek R, Kropff M, Petrucci MT, et al (2010). A phase III study to determine the efficacy and safety of lenalidomide combined with melphalan and prednisone in patients > 65 years with newly diagnosed multiple myeloma (NDMM) [abstract]. *Haematologica*; 95(234): 566.

Benbouker L, Dimopoulos M, Dispenzieri A Catalano J Belch AR, Cavo M, Pinto A, Weisel K, Ludwig H, Bahlis N, Banos A, Tiab M, Delforge M, Cavenagh J, Geraldes C, Lee JJ, Chen C, Oriol A, De La Rubia J, Qiu L, White DJ, Binder D, Anderson K, Fermand JP, Moreau P, Attal M, Knight R, Chen G, Van Oostendorp J, Jacques C, Ervin-Haynes A, Avet-Loiseau H, Hulin C and Facon T for the FIRST Trial Team. (2014). Lenalidomide and dexamethasone in transplant-ineligible patients with myeloma. *N Engl J Med*; 371(10): 906-17.

Proteosome Inhibition / Velcade

Adams J, Palombella VJ, Sausville EA, et al. (1999). Proteosome inhibitors: a novel class of potent and effective antitumor agents. *Cancer Res:* 1999;59:2615-22.

Aghajanian C, Soignet S, Dizon DS, et al. (2002). A Phase I Trial of the Novel Proteosome Inhibitor PS341 in Advanced Solid Tumor Malignancies. *Clin Cancer Res:* 8: 2505-11.

Cusack JC, Jr., Liu R, Houston M, et al. (2001). Enhanced chemosensitivity to CPT-11 with proteosome inhibitor PS-341: implications for systemic nuclear factor-kappaB inhibition. *Cancer Res:* 61:3535-40.

Ha MH et al. (2003). The proteosome inhibitor PS-341 markedly enhances sensitivity of multiple myeloma tumour cells to chemotherapeutic agents. *Clin Cancer Res:* 9:1136-44.

APPENDIX I – DOSE MODIFICATIONS FOR VCD

Before each dose, participants should be evaluated for possible toxicities which may have occurred. Dose modifications will be required for the situations detailed below. Please refer to the current Summary of Product Characteristics for further details. Dose modifications and delays different from those stated in the protocol, for management of toxicities are at the discretion of the local Principal Investigator.

**Neutropenia**	If the neutrophil count falls below $0.5x10^9$/L then the dose of cyclophosphamide can be omitted, if the count is particularly low the bortezomib therapy should be withheld

Hideshima T, Richardson P, Chauhan D, et al. (2001). The proteosome inhibitor PS-341 inhibits growth, induces apoptosis, and overcomes drug resistance in human multiple myeloma cells. *Cancer Res:* 61:3071-6.

Jagannath S, Barlogie B, Berenson JR et al. (2005). Bortezomib in recurrent and/or refractory multiple myeloma. *Cancer:* 103: 1195–200.

Jagannath S, Durie BG, Wolf J, et al. (2005). Bortezomib therapy alone and in combination with dexamethasone for previously untreated symptomatic multiple myeloma. *BJH*: 129:776-83.

Jelinsky SA, Samson LD. (1999). Global response of Saccharomyces cerevisiae to an alkylating agent. *Proc Natl Acad Sci USA:* 96: 1486-1491.

LeBlanc R, Catley LP, Hideshima T, et al. (2002). Proteosome inhibitor PS-341 inhibits human myeloma cell growth in vivo and prolongs survival in a murine model. *Cancer Res:* 62:4996-5000.

Lee A-H et al. (2003). Proteosome inhibitors disrupt the unfolded protein response in myeloma cells. *PNAS 100*: 9946-51.

Lightcap ES, McCormack TA, Pien CS, et al. (2000). Proteosome inhibition measurements: clinical application. *Clin Chem:* 46:673-83.

Orlowski RZ, Stinchcombe TE, Mitchell BS, et al. (2002). Phase I Trial of the Proteosome Inhibitor PS-341 in Patients with Refractory Hematologic Malignancies. *J Clin Oncol:* 20:4420-7.

McConkey D, Williams S, Papandreou. (2000). Role of p53 in proteosome inhibitor-based combination chemotherapy in LNCAP cells. *Proc Am Assoc Cancer Res*: 41:651. Abstract 4136.

Mitsiades N et al. (2003). The proteosome inhibitor PS-341 potentiates sensitivity of multiple myeloma cells to conventional chemotherapeutic agents: therapeutic applications. *Blood*: 101: 2377-80.

Pei XY et al. (2003). The proteosome inhibitor Velcade promotes mitochondrial injury and apoptosis induced by the small molecule Bcl-2 inhibitor HA14-1 in multiple myeloma cells. *Leukemia*: 17:2036-45.

Richardson PG, Barlogie B, Berenson J, et al. (2003). A phase II study of bortezomib in relapsed, refractory myeloma. *NEJM*: 348: 2609-17.

Richardson PG, Sonneveld P, Schuster MW, et al. (2005). Bortezomib or high-dose dexamethasone for relapsed multiple myeloma. *NEJM*: 352: 2487-98.

VELCADE (Velcade). Cambridge MA: Millennium Pharmaceuticals, Inc. 2003.

Vorinostat

Badros A, Burger AM, Philip S, Niesvizky R, Kolla SS, Goloubeva O, Harris C, Zwiebel J, Wright JJ, Espinoza-Delgado I, Baer MR, Holleran JL, Egorin MJ, Grant S (2009). Phase I

study of vorinostat in combination with bortezomib for relapsed and refractory multiple myeloma. *Clin Cancer Res.* 15(16): 5250-7. Epub 2009 Aug 11.

Campbell RA, Sanchez E, Steinberg J, Shalitin D, Li ZW, Chen H, Berenson JR (2010). Vorinostat enhances the antimyeloma effects of melphalan and bortezomib. *Eur J Haematol.* 84(3): 201-11. Epub 2009 Nov 18.

Mazumder A, Vesole DH, Jagannath S (2010). Vorinostat plus bortezomib for the treatment of relapsed/refractory multiple myeloma: a case series illustrating utility in clinical practice. *Clin Lymphoma Myeloma Leuk*. 1;10(2):149-51.

Mitsiades N, Mitsiades CS, Richardson PG, McMullan C, Poulaki V, Fanourakis G, Schlossman R, Chauhan D, Munshi NC, Hideshima T, Richon VM, Marks PA, Anderson KC (2003). Molecular sequelae of histone deacetylase inhibition in human malignant B cells. *Blood*. 101(10):4055-62. Epub 2003 Jan 16.

O'Connor OA, Heaney ML, Schwartz L, Richardson S, Willim R, MacGregor-Cortelli B, Curly T, Moskowitz C, Portlock C, Horwitz S, Zelenetz AD, Frankel S, Richon V, Marks P, Kelly WK (2006). Clinical experience with intravenous and oral formulations of the novel histone deacetylase inhibitor suberoylanilide hydroxamic acid in patients with advanced hematologic malignancies. *J Clin Oncol*. 24(1):166-73. Epub 2005 Dec 5.

Pei XY, Dai Y, Grant S (2004). Synergistic induction of oxidative injury and apoptosis in human multiple myeloma cells by the proteasome inhibitor bortezomib and histone deacetylase inhibitors. *Clin Cancer Res*. 10(11): 3839-52.

Carfilzomib

Alsina M, Trudel S, Furman RR, Rosen PJ, O'Connor OA, Comenzo RL, Wong A, Kunkel LA, Molineaux CJ, Goy A. A phase I single-agent study of twice-weekly consecutive-day dosing of the proteasome inhibitor carfilzomib in patients with relapsed or refractory multiple myeloma or lymphoma. Clin Cancer Res. 2012 Sep 1;18(17):4830-40.

Siegel DS, Martin T, Wang M, Vij R, Jakubowiak AJ, Lonial S, Trudel S, Kukreti V, Bahlis N, Alsina M, Chanan-Khan A, Buadi F, Reu FJ, Somlo G, Zonder J, Song K, Stewart AK, Stadtmauer E, Kunkel L, Wear S, Wong AF, Orlowski RZ, Jagannath S.A phase 2 study of single-agent carfilzomib (PX-171-003-A1) in patients with relapsed and refractory multiple myeloma. Blood. 2012 Oct 4;120(14):2817-25.

Vij R, Wang M, Kaufman JL, Lonial S, Jakubowiak AJ, Stewart AK, Kukreti V, Jagannath S, McDonagh KT, Alsina M, Bahlis NJ, Reu FJ, Gabrail NY, Belch A, Matous JV, Lee P, Rosen P, Sebag M, Vesole DH, Kunkel LA, Wear SM, Wong AF, Orlowski RZ, Siegel DS.An open-label, single-arm, phase 2 (PX-171-004) study of single-agent carfilzomib in bortezomib-naive patients with relapsed and/or refractory multiple myeloma. Blood. 2012 Jun 14;119(24):5661-70.

Vij R, Siegel DS, Jagannath S, Jakubowiak AJ, Stewart AK, McDonagh K, Bahlis N, Belch A, Kunkel LA, Wear S, Wong AF, Wang M. An open-label, single-arm, phase 2 study of single-agent carfilzomib in patients with relapsed and/or refractory multiple myeloma who have been previously treated with bortezomib. Br J Haematol. 2012 Sep;158(6):739-48.

Jagannath S, Vij R, Stewart AK, Trudel S, Jakubowiak AJ, Reiman T, Somlo G, Bahlis N, Lonial S, Kunkel LA, Wong A, Orlowski RZ, Siegel DS.An open-label single-arm pilot phase

II study (PX-171-003-A0) of low-dose, single-agent carfilzomib in patients with relapsed and refractory multiple myeloma. Clin Lymphoma Myeloma Leuk. 2012 Oct;12(5): 310-8.

Carfilzomib in multiple myeloma patients with renal impairment: pharmacokinetics and safety. Badros AZ, Vij R, Martin T, Zonder JA, Kunkel L, Wang Z, Lee S, Wong AF, Niesvizky R. Leukemia. 2013 Jan 31. doi: 10.1038/leu.2013.29. [Epub ahead of print]

Jakubowiak AJ, Dytfeld D, Griffith KA, Lebovic D, Vesole DH, Jagannath S, Al-Zoubi A, Anderson T, Nordgren B, Detweiler-Short K, Stockerl-Goldstein K, Ahmed A, Jobkar T, Durecki DE, McDonnell K, Mietzel M, Couriel D, Kaminski M, Vij R. A phase 1/2 study of carfilzomib in combination with lenalidomide and low-dose dexamethasone as a frontline treatment for multiple myeloma. Blood. 2012 Aug 30;120(9):1801-9.

Niesvizky R, Martin TG 3rd, Bensinger W, Alsina M, Siegel DS, Kunkel LA, Wong AF, Lee SJ, Orlowski RZ, Wang M Phase Ib Dose-escalation Study (PX-171-006) of Carfilzomib, Lenalidomide, and Low-Dose Dexamethasone in Relapsed or Progressive Multiple Myeloma. Clin. Cancer Res. 2013 Apr 15, 19(8):2248-56

Anticoagulation

Zangari M, Siegel E, Barlogie B, Anaissie E, Saghafifar F, Fassas A, Morris C, Fink L, Tricot G. (2002). Thrombogenic activity of doxorubicin in myeloma patients receiving thalidomide: implications for therapy. *Blood*: 100: 1168-71.

Palumbo A, Rajkumar SV, Dimopoulos MA, Richardson PG, San Miguel J, Barlogie B, Harousseau J, Zonder JA, Cavo M, Zangari M, Attal M, Belch A, Knop S, Joshua D, Sezer O, Ludwig H, Vesole D, Bladé J, Kyle R, Westin J, Weber D, Bringhen S, Niesvizky R, Waage A, von Lilienfeld-Toal M, Lonial S, Morgan GJ, Orlowski RZ, Shimizu K, Anderson KC, Boccadoro M, Durie BG, Sonneveld P, Hussein MA; International Myeloma Working Group. (2008). Prevention of thalidomide- and lenalidomide-associated thrombosis in myeloma. *Leukemia:Feb*;22:414-23.

Statistical

O'Brien, P.C and Fleming, T.R. (1979). A Multiple Testing Procedure for Clinical Trials. *Biometrics*: 35:549–556.

Gehan EA. (1961). The determination of the number of patients required in a preliminary and a follow-up trial of a new chemotherapeutic agent. *Journal of Chronic Diseases:* 13: 346-53.

Freidlin B, Korn EL, and Gray, R. (2010). A general inefficacy interim monitoring rule for randomized clinical trials. *Clin Trials*: 7:197-208.

21. APPENDICES

APPENDIX A – DEFINITION OF MYELOMA AND RELATED DISEASES

British Journal of Haematology 2003; 121: 749-757

Monoclonal Gammopathy of Undetermined Significance (MGUS) or Monoclonal Gammopathy, Unattributed/Unassociated (MG[u])

- M-protein in serum <30 g/L
- Bone marrow clonal plasma cells <10% and minimal plasma cell infiltration of a trephine biopsy (if done)
- No evidence of other B-cell proliferative disorders
- No related organ or tissue impairment (end organ damage)

Myeloma Related Organ or Tissue Impairment (end organ damage) Due to the Plasma Cell Proliferative Process

- * Calcium levels increased: serum calcium >10 mg/L (0.25 mmol/L) above normal or >110 mg/L (2.75 mmol/L)
- * Renal Insufficiency: creatinine >20 mg/L (173 mmol/L)
- * Anaemia: haemoglobin 2 g/dL below normal or haemoglobin <10 g/dL
- * Bone lesions: Lytic lesions or osteoporosis with compression fractures (MRI or CT may clarify)
- Other: symptomatic hyperviscosity, amyloidosis, recurrent bacterial infections (>2 episodes in 12 months)

*(****CRAB)*

Asymptomatic Myeloma

- M-protein in serum ≥30 g/L
- and/or Bone marrow clonal plasma cells ≥10%
- No related organ or tissue impairment (no end organ damage, including bone lesions) or symptoms

Symptomatic Multiple Myeloma

- M-protein in serum and/or urine
- Bone marrow (clonal) plasma cells* or plasmacytoma
- Related organ or tissue impairment (end organ damage, including bone lesions)

** If flow cytometry is performed most plasma cells (>90%) will show a 'neoplastic' phenotype.*

Nonsecretory Myeloma

- No M-protein in serum and/or urine with immunofixation
- Bone marrow clonal plasmacytosis ≥10% or plasmacytoma
- Related organ or tissue impairment (end organ damage, including bone lesions)

Solitary Plasmacytoma of Bone

- No M-protein in serum and/or urine*
- Single area of bone destruction due to clonal plasma cells
- Bone marrow not consistent with multiple myeloma
- Normal skeletal survey (and MRI of spine and pelvis if done)

- No related organ or tissue impairment (no end organ damage other than solitary bone lesion)

** A very small M-component may sometimes be present*

Extramedullary Plasmacytoma

- No M-protein in serum and/or urine*
- Extramedullary tumour of (clonal) plasma cells
- Normal bone marrow
- Normal skeletal survey
- No related organ or tissue impairment (end organ damage including bone lesions)

Multiple Solitary Plasmacytoma (±Recurrent)

- No M-protein in serum and/or urine*
- More than one localised area of bone destruction or extramedullary tumour of clonal plasma cells which may be recurrent
- Normal bone marrow
- Normal skeletal survey and MRI of spine and pelvis if done
- No related organ or tissue impairment (no end organ damage other than the localised bone lesions)

** A very small M-component may sometimes be present*

APPENDIX B – WHO GRADES OF PERFORMANCE STATUS

Grade	Summary	Description of performance status
0	Normal	Able to carry out all normal activity without restriction
1	With effort	Restricted in physically strenuous activity; ambulatory, can do light work
2	Restricted	Ambulatory and capable of all self-care but unable to carry out any work; up and about more than 50% of waking hours
3	Dependent	Capable of only limited self-care; confined to bed or chair for more than 50% of waking hours
4	Immobile	Completely disabled; cannot carry out any self-care; totally confined to bed or chair

APPENDIX C – DEFINITIONS OF RESPONSE

International Uniform Response criteria of Response and Progression (Blade et al, 1998; Durie et al, 2006; Rajkumar et al, 2011)

Paraprotein responses should only be calculated using sequential paraprotein measurements made in the same laboratory using the same method.

All response categories require 2 consecutive assessments made at any time before the institution of any new therapy. All categories also require no known evidence of progressive or new bone lesions if radiographic studies were performed. Radiographic studies are not required to satisfy these response requirements.

Complete Response (CR) requires all the following:

1. Absence of the original monoclonal paraprotein in serum / urine by routine electrophoresis and immunofixation. The presence of oligoclonal bands consistent with oligoclonal immune reconstitution does not exclude CR.
2. < 5% plasma cells in bone marrow (confirmation with repeat bone marrow is not needed)
3. No increase in size or number of lytic bone lesions on radiological investigations, if performed (development of a compression fracture does not exclude response).
4. Disappearance of soft tissue plasmacytomas.
5. For patients with light chain myeloma (the serum and urine M-protein are unmeasurable), a normal FLC ratio of 0.26 to 1.65 (or laboratory-specific normal FLC ratio reference range) in addition to the CR criteria above.

Patients in whom some, but not all, of the criteria for CR are fulfilled are classified as VGPR. This includes patients in whom electrophoresis is negative but in whom immunofixation has not been performed.

Very Good Partial Response (VGPR)

1. Serum and urine M-protein detectable by immunofixation but not on electrophoresis, **OR**
2. ≥90% reduction in the serum monoclonal paraprotein level plus urinary light chain excretion < 100 mg/24 hours, if measured.
3. No increase in size or number of lytic bone lesions on radiological investigations, if performed.
4. For patients with light chain myeloma (the serum and urine M-protein are unmeasurable), >90% decrease in the difference between involved and uninvolved FLC levels.

Partial Response (PR)

1. ≥ 50% reduction in the serum monoclonal paraprotein level, and.
2. Reduction in 24-hour urinary light chain excretion either by ≥90% or to < 200 mg/24 hours, if measured.
3. For patients with light chain myeloma (the serum and urine M-protein are unmeasurable), ≥ 50% reduction in the difference between involved and uninvolved serum FLC levels.
4. For patients with non-secretory myeloma only, ≥ 50% reduction in plasma cells in bone marrow, provided baseline percentage was ≥ 30%.
5. In addition, ≥ 50% reduction in the size of soft tissue plasmacytoma, if present at baseline.

6. No increase in size or number of lytic bone lesions on radiological investigations, if performed.

Patients in whom some, but not all, of the criteria for PR are fulfilled are classified as MR.

Minimal Response (MR) requires all the following

1. 25-49% reduction in the serum monoclonal paraprotein level.
2. 50-89% reduction in 24-hour urinary light chain excretion, which still exceeds 200 mg/24 hours, if measured.
3. For patients with non-secretory myeloma only, 25-49% reduction in plasma cells in bone marrow.
4. 25-49% reduction in the size of soft tissue plasmacytomas.
5. No increase in size or number of lytic bone lesions on radiological investigations, if performed.

MR also includes patients in whom some, but not all, of the criteria for PR are fulfilled.

No Change (NC)

Not meeting the criteria of either minimal response or progressive disease.

Progressive Disease (PD) requires one or more of the following:

1. ≥ 25% increase from lowest response level in the serum monoclonal paraprotein level which must also be an absolute increase of at least 5g/L and confirmed by at least one repeated investigation.
2. ≥ 25% increase from lowest response level in 24-hour urinary light chain excretion, if measured, which must also be an absolute increase of at least 200 mg/24 hours and confirmed by at least one repeated investigation.
3. For patients with light chain myeloma (the serum and urine M-protein are unmeasurable), ≥ 25% increase from lowest response level in the difference between involved and uninvolved serum FLC levels, confirmed by at least one repeated investigation. The absolute increase must be > 100 mg/L.
4. ≥ 25% increase in plasma cell percentage in bone marrow, which must also be an absolute percentage of at least 10%.
5. Definite increase in the size of existing lytic bone lesions or soft tissue plasmacytomas.
6. Development of new lytic bone lesions or soft tissue plasmacytomas. Development of a compression fracture does not exclude continued response.
7. Development of hypercalcaemia (corrected >2.65mmol/L) not attributable to any other cause.

Plateau

Stable values (within 25% above or below value at time response is assessed) maintained for at least 3 months.

Relapse from CR requires at least one of the following:

1. Reappearance of serum or urinary paraprotein on routine electrophoresis or on immunofixation confirmed by at least one further investigation and excluding oligoclonal immune reconstitution.
2. ≥ 5% plasma cells in bone marrow.
3. Development of new lytic bone lesions or soft tissue plasmacytomas or definite increase in the size of residual bone lesions. Development of a compression fracture does not exclude continued response.

4. Development of hypercalcaemia (corrected >2.8mmol/L) not attributable to any other cause.

Maximum response

The following definition of 'Maximum paraprotein response' is provided as guidance on determining when a patient has achieved maximum response and thus therapy can be stopped. It is not from the International Uniform Response criteria.

Maximum paraprotein response has been achieved when:

The difference in paraprotein levels at the end of the last two cycles of chemotherapy is < 25% above or below the level at the start of those two cycles of chemotherapy. For whole paraprotein, the absolute difference must be >5g/l. If the reduction in paraprotein level is ≥ 25% over the last two cycles, then therapy should continue.

Or

For patients with light chain myeloma (the serum and urine M-protein are unmeasurable), < 25% difference between involved and uninvolved serum FLC levels above or below the level at the start of those two cycles of chemotherapy. The absolute difference must be >100mg/L. If the reduction in uninvolved and involved serum FLC level is ≥ 25% over the last two cycles, then therapy should continue.

APPENDIX D – NATIONAL CANCER INSTITUTE COMMON TOXICITY CRITERIA (NCIC)

Toxicities will be assessed based on the National Cancer Institute Common Terminology Criteria for Adverse Events V4.0 (NCI-CTCAE). A copy is provided in the Investigator Site File and may be obtained at:
http://evs.nci.nih.gov/ftp1/CTCAE/About.html

APPENDIX E – LOCAL INVESTIGATIONS AND SAMPLE COLLECTION FOR CENTRAL INVESTIGATION

LOCAL INVESTIGATIONS (BOTH PATHWAYS)

	**Baseline** (Within 4 weeks prior to randomisation unless otherwise specified)*	**End of each treatment cycle** (CTD(a), RCD(a), CCRD or VCD)	**During maintenance treatment** For each treatment cycle	**2 / 3 monthly follow up****** (Following induction treatment, +/- VCD, +/- HDM/ASCT until disease progression)	**Disease progression** (Progressive disease during induction therapy not included)
WHO Performance Status	✓				
Physical examination	✓				
Medical history (including review for prior cancers)	✓				
Paraprotein (protein electrophoresis of serum) and immunofixation	✓	✓		✓	✓
IgA, IgG, IgM quantification	✓	✓		✓	✓
Serum Free Light Chain analysis	✓	✓		✓	✓
Urinary light chain excretion (24hr urine sample)	✓	✓		✓	✓
Bone marrow (aspirate and trephine) (must include sufficient sample for central investigations, if consented)	✓ (within 2 months before randomisation)	✓ in patients who become immunofixation negative, to confirm CR			✓
Full blood count and differential	✓	✓	✓	✓	
Biochemistry (to include calcium, urea, creatinine, albumin/LFTs and uric acid)	✓	✓	✓	✓	
ß2-microglobulin (ß2M)	✓				
Lactate dehydrogenase (LDH)	✓				
C-Reactive protein (CRP)	✓				
Pregnancy test** (for women of childbearing potential as defined in Appendix G)	Within 72 hours before start of thalidomide / lenalidomide treatment, 4-weekly as a minimum during thalidomide/ lenalidomide treatment and 4 weeks after last dose of thalidomide/ lenalidomide				
Axial skeletal survey or unenhanced whole body CT (see Appendix C and Appendix F)	✓ (up to 2 weeks after starting treatment)				
***MRI may be helpful, particularly for patients with suspected spinal cord compression	✓ (not compulsory)				
***CT may be helpful in assessing extramedullary disease	✓ (not compulsory)				
Lumbar and thoracic spine X-rays (PA and Lateral)	Should be performed in accordance with local policy.				
Clinical assessment (including monitoring for clinical symptoms of disease progression)	✓	✓	✓	✓	✓
Assessment of adverse events (including adverse events and second primary malignancy)	✓	✓	✓	✓	✓

** All local baseline investigations to confirm eligibility are performed within 4 weeks prior to randomisation. All other baseline investigations must be performed prior to starting protocol treatment, unless otherwise specified.*
*** Women of childbearing potential (WCBP) must have a negative pregnancy test performed by a healthcare professional in accordance with the Celgene thalidomide and lenalidomide Pregnancy Prevention Programmes*
**** May be performed as part of routine care however not mandatory for trial participation*
***** 2-monthly for the first 2 years post initial randomisation and 3 monthly thereafter until disease progression*

INTENSIVE PATHWAY Sample collection for central investigations (if patient has consented to laboratory investigations)

Sample	Investigation	Diagnosis (patient consented on NHS form)[1]	Trial consent	Baseline (post trial consent and before start of treatment)	Diagnosis of haematological Second Primary Malignancy[2]	Post cycle 1 and cycle 3 CTD/RCD/CCRD	Post induction treatment (CTD/RCD/CCRD)	Post VCD treatment	Stem cell harvest	1-2 weeks Post-HDT	3 months Post-HDT	2-monthly for the first 2 years & then 3-monthly to relapse	6 months post maintenance randomisation	Relapse
10 mL clotted peripheral blood Random urine sample<br>**SEND TO BIRMINGHAM**	Creatinine, IgA, IgG, IgM, paraprotein, serum free light chain, BJP, β_2M		**Consented to Myeloma XI trial and central laboratory investigations**	✓		✓	✓	✓		✓<br>Plus 2 mL EDTA peripheral blood	✓	✓	✓	✓
0.5 mL of stem cell harvest (fresh, or thawed)<br>**SEND 1ST CLASS TO BIRMINGHAM AND ICR**	T, B and NK cell subset analysis								✓					
5 mL EDTA bone marrow aspirate<br>5 mL EDTA peripheral blood<br>**SEND TO ICR, LONDON**	Plasma cell percentage, phenotype and FISH<br>Genomic DNA<br>RNA expression profiling	✓<br>Bone marrow only (including 3 bone marrow smears)		✓<br>Bone marrow aspirate & smears (if not sent previously) & peripheral blood			✓<br>Bone marrow aspirate only	✓<br>Bone marrow aspirate only			✓<br>Bone marrow aspirate only		✓<br>Bone marrow aspirate only	✓
0.5 mL EDTA bone marrow aspirate<br>**SEND TO HMDS, LEEDS**	Minimal residual disease	✓		✓<br>(if not sent previously)			✓	✓			✓		✓	
5 mL EDTA bone marrow aspirate, and trephine if available<br>**SEND TO ICR, LONDON**	Confirmation of diagnosis				✓<br>If bone marrow is being taken for SPM diagnostic purposes or as part of routine care									

[1] Baseline bone marrow samples may be taken before trial consent, provided that patients have consented to these samples being sent using the standard NHS consent form.

[2] A sample should be sent to ICR upon diagnosis of a haematological second primary malignancy if the participant is having a bone marrow taken as part of standard care and they have consented to trial samples being taken.

NON-INTENSIVE PATHWAY Sample collection for central investigations (If patient has consented to laboratory investigations)

Sample	Investigation	Diagnosis (patient consented on NHS form)[1]	Trial consent	Baseline (post trial consent and before start of treatment)	Diagnosis of haematological Second Primary Malignancy[2]	Post cycle 1 and cycle 3 CTDa/ RCDa	Post induction treatment (CTD/RCD)	Post VCD treatment	3 months post end of treatment CTD/RCD +/- VCD	2-monthly for the first 2 years & then 3-monthly to relapse	6 months post maintenance randomisation	Relapse
10 mL clotted peripheral blood Random urine sample **SEND TO BIRMINGHAM**	Creatinine, IgA, IgG, IgM, paraprotein, serum free light chain, BJP, $ß_2M$		**Consented to Myeloma XI trial and central laboratory investigations**	✓		✓	✓	✓	✓	✓	✓	✓
5 mL EDTA bone marrow aspirate 5 mL EDTA peripheral blood **SEND TO ICR, LONDON**	Plasma cell percentage, phenotype, FISH, Genomic DNA RNA expression profiling	✓ Bone marrow only (including 3 bone marrow smears)		✓ Bone marrow aspirate & smears (if not sent previously) & peripheral blood			✓ Bone marrow only	✓ Bone marrow only	✓ Bone marrow only		✓ Bone marrow only	✓
0.5 mL EDTA bone marrow aspirate **SEND TO HMDS, LEEDS**	Minimal Residual Disease	✓		✓ (if not sent previously)			✓	✓	✓		✓	
5 mL EDTA bone marrow aspirate Trephine if available **SEND TO ICR, LONDON**	Confirmation of diagnosis				✓ If bone marrow is being taken for SPM diagnostic purposes or as part of routine care							

1 Baseline bone marrow samples may be taken before trial consent, provided that patients have consented to these samples being sent using the standard NHS consent form.

2 A sample should be sent to ICR upon diagnosis of a haematological second primary malignancy if the participant is having a bone marrow taken as part of standard care and they have consented to trial samples being taken.

APPENDIX F – AXIAL SKELETAL SURVEY

The following axial skeletal survey images should be taken at presentation (taken from Royal College Guidelines):

**Skull**	LATERAL view
**Shoulders**	Both AP views only, on bucky to include clavical
**Cervical spine**	AP, LATERAL and OPEN MOUTH view
**Dorsal spine**	AP and LATERAL
**Lumbar spine**	AP and LATERAL
**Pelvis**	AP to include upper femora
**Chest**	PA THORAVISION FILM. If not available, do SUPINE BUCKY film for RIBS

NB: In addition, any part that is affected by pain should also be examined.

For all skeletal surveys, it is essential to have good quality films with fine bone detail.

It is permissible to use unenhanced whole body CT rather than skeletal survey where that is local policy.

APPENDIX G – DEFINITION OF A WOMAN OF CHILDBEARING POTENTIAL

A woman of childbearing potential (WCBP) is:

- a sexually mature woman (i.e. any female who has ever experienced menstrual bleeding) **AND**
- who has not undergone a hysterectomy or who has not been postmenopausal for at least 24 consecutive months (i.e. who has had menses at any time within the preceding 24 consecutive months). Amenorrhoea following cancer therapy does not rule out childbearing potential.

The following are examples of highly effective and additional effective methods of contraception:

Highly effective methods:
- Intrauterine device (IUD)
- Hormonal (birth control pills, injections, implants, levonorgestrel-releasing intrauterine system [IUS], medroxyprogesterone acetate depot injections, ovulation inhibitory progesterone-only pills [e.g. desogestrel])
- Tubal ligation
- Partner's vasectomy

Additional effective methods:
- Male condom
- Diaphragm
- Cervical cap

APPENDIX H – DOSE MODIFICATIONS FOR INDUCTION REGIMENS

Lenalidomide starting dose	25 mg
Dose level -1	15 mg
Dose level -2	10 mg
Dose level -3	5 mg

Carfilzomib current dose	Reduce to*
36 mg/m^2	27 mg/m^2
27 mg/m^2	20 mg/m^2
20 mg/m^2	15 mg/m^2

** If the participant tolerates the reduced dose for two cycles, participant may be dose escalated to the dose prior to reduction at the discretion of the treating clinician.*

<table>
<tr><th></th><th>CTD</th><th>CTDa</th><th>RCD</th><th>RCDa</th><th>CCRD</th></tr>
<tr><td>Thromboembolism</td><td colspan="5">The occurrence of a thromboembolic event such as a DVT or pulmonary embolism is an indication for full anticoagulation following standard treatment guidelines. Thalidomide or lenalidomide may be stopped, but can be re-introduced, assuming good anticoagulant control and no other untoward side effects</td></tr>
<tr><td rowspan="3">Adjustments for renal insufficiency</td><td colspan="5">If, despite the continuation of vigorous hydration, the serum creatinine is >300µmol/L, cyclophosphamide is omitted.</td></tr>
<tr><td rowspan="2"></td><td rowspan="2"></td><td colspan="3">Lenalidomide dose should be adjusted in accordance with the lenalidomide SPC.

Lenalidomide is substantially excreted by the kidney, therefore care should be taken in dose selection and monitoring of renal function is advised. No dose adjustments are required for patients with mild renal impairment. The following dose adjustments are recommended at the start of therapy for patients with moderate or severe impaired renal function or end stage renal disease.

<table><tr><th>Renal Function (CrCl)</th><th>Dose Adjustment</th></tr><tr><td>Moderate renal impairment
(30 ≤ CrCl < 50 mL/min)</td><td>10 mg once daily*</td></tr><tr><td>Severe renal impairment
(CrCl < 30 mL/min, not requiring dialysis)</td><td>15 mg every other day**</td></tr><tr><td>End stage renal failure
(CrCl < 30 mL/min, requiring dialysis)</td><td>5 mg once daily. On dialysis days, the dose should be administered following dialysis</td></tr></table>* The dose may be escalated to 15 mg once daily after 2 cycles if patient is not responding to treatment and is tolerating the treatment.
** The dose may be escalated to 10 mg once daily if the patient is tolerating the treatment.</td></tr>
<tr><td></td><td></td><td>Carfilzomib should be held for CrCl < 15 mL/min</td></tr>
</table>

<table>
<tr><th></th><th>CTD</th><th>CTDa</th><th>RCD</th><th>RCDa</th><th>CCRD</th></tr>
<tr><td>Adjustments for neutropenia and/or thrombocytopenia (also see below)</td><td colspan="5">Evidence of myelosuppression prior to initial treatment is likely to be a reflection of bone marrow infiltration. Unless there is evidence suggesting another cause, participants should be given at least the first cycle at full dose. If the cytopenias are treatment-related, omission of cyclophosphamide for 1-3 weeks and then a dose reduction e.g. to 400 mg or 300 mg, would be reasonable. The use of G-CSF is entirely appropriate and may remove the need for amendment of the regimen.</td></tr>
<tr><td rowspan="2">Neutropenia</td><td></td><td></td><td colspan="3">Lenalidomide treatment must not be started if the Absolute Neutrophil Counts (ANC) <1.0 x 10^9/l, and/or platelet counts <75 x 10^9/l or, dependent on bone marrow infiltration by plasma cells, platelet counts <30 x 10^9/l.
<table>
<tr><th>When neutrophils</th><th>Recommended course</th></tr>
<tr><td>First fall to <0.5 x 10^9/L</td><td>Interrupt lenalidomide treatment</td></tr>
<tr><td>Return to ≥0.5 x 10^9/L when neutropenia is the only observed toxicity</td><td>Resume lenalidomide at Starting Dose once daily</td></tr>
<tr><td>Return to ≥0.5 x 10^9/L when dose-dependent haematological toxicities other than neutropenia are observed</td><td>Resume lenalidomide at next lower dose level once daily</td></tr>
<tr><td>For each subsequent drop to <0.5 x 10^9/L</td><td>Interrupt lenalidomide treatment</td></tr>
<tr><td>Return to ≥0.5 x 10^9/L</td><td>Resume lenalidomide at next lower dose level (Dose level -2 and -3) once daily. Do not dose below 5mg once daily.</td></tr>
</table></td></tr>
<tr><td></td><td></td><td></td><td></td><td>
<table>
<tr><th>When ANC</th><th>Recommended Action Carfilzomib</th></tr>
<tr><td>Falls to < 0.5 × 10^9/L</td><td>Interrupt carfilzomib add growth factor if Gr 3 with fever or Gr 4, follow FBC weekly</td></tr>
<tr><td>Returns to > 1.0 × 10^9/L (if neutropenia was the only toxicity noted)</td><td>Resume at full dose</td></tr>
<tr><td>Returns to > 1.0 × 10^9/L (if other toxicity noted)</td><td>Resume at 1 dose decrement</td></tr>
<tr><td>Subsequently drops to < 0.5 × 10^9/L</td><td>Interrupt carfilzomib</td></tr>
<tr><td>Returns to > 1.0 × 10^9/L</td><td>Resume at 1 dose decrement</td></tr>
</table></td></tr>
</table>

<table>
<tr><th></th><th>CTD</th><th>CTDa</th><th>RCD</th><th>RCDa</th><th>CCRD</th></tr>
<tr><td></td><td></td><td></td><td></td><td></td><td></td></tr>
<tr><td rowspan="2">Thrombocytopenia</td><td rowspan="2"></td><td rowspan="2"></td><td colspan="3">Lenalidomide treatment must not be started if the Absolute Neutrophil Counts (ANC) <1.0 x 10^9/l, and/or platelet counts <75 x 10^9/l or, dependent on bone marrow infiltration by plasma cells, platelet counts <30 x 10^9/l.

<table>
<tr><th>When platelets</th><th>Recommended course</th></tr>
<tr><td>First fall to <30 x 10^9/L</td><td>Interrupt lenalidomide treatment</td></tr>
<tr><td>Return to ≥30 x 10^9/L</td><td>Resume lenalidomide at next lower dose level once daily</td></tr>
<tr><td>For each subsequent drop below 30x10^9/L</td><td>Interrupt lenalidomide treatment</td></tr>
<tr><td>Return to ≥30 x 10^9/L</td><td>Resume lenalidomide at next lower dose level (Dose Level -2 and -3) once daily. Do not dose below 5mg once daily.</td></tr>
</table>
</td></tr>
<tr><td></td><td></td><td>
<table>
<tr><th>When Platelets:</th><th>Recommended Action Carfilzomib</th></tr>
<tr><td>Fall to <25×10^9/L (with active bleeding)</td><td>Interrupt carfilzomib, follow FBC weekly</td></tr>
<tr><td>Return to ≥25×10^9/L</td><td>Resume at full dose</td></tr>
<tr><td>Subsequently drop to <25 × 10^9/L (with active bleeding)</td><td>Interrupt carfilzomib, follow FBC weekly</td></tr>
<tr><td>Return to ≥25×10^9/L</td><td>Resume at 1 dose decrement</td></tr>
</table>
Carfilzomib will be withheld from participants with Grade 4 thrombocytopenia with active bleeding.

Grade 4 anaemia and thrombocytopenia without active bleeding does not require the carfilzomib dose to be withheld. However, participants should receive supportive measures in accordance with institutional guidelines. For participants with Grade 4 thrombocytopenia without
</td></tr>
</table>

	CTD	CTDa	RCD	RCDa	CCRD
					evidence of bleeding, study drug dosing may occur at the discretion of the investigator.
**Other side effects (thalidomide or lenalidomide)**	Thalidomide-related grade 1-2 toxicity, but sometimes grade 3-4 toxicity, may be encountered and include constipation, neuropathy, fatigue, sedation, rash, tremor and oedema. Grade 3-4 toxicity is an indication to stop thalidomide for the remainder of the current cycle and then re-introduce at 50mg daily with the next or subsequent cycle. Assuming tolerance at the lower dose level, escalation to 100 mg daily may be considered, and possibly to 150 mg or the full dose of 200 mg daily if the symptoms resolve and do not recur.		Lenalidomide-related grade 1-2 toxicity, but sometimes grade 3-4 toxicity, may be encountered. Grade 3-4 toxicity is an indication to stop lenalidomide for the remainder of the current cycle and then re-introduce at a lower dose with the next or subsequent cycle.		
**Other side effects (dexamethasone)**	Occasionally participants will be unable to tolerate dexamethasone at the protocol doses because of various corticosteroid effects. Dose reduction, would be a reasonable first step. Omission of one of the two 4-day pulses of dexamethasone in a treatment cycle would be an alternative approach. Switching to an alternative corticosteroid, e.g. methylprednisolone, although rarely appropriate, would also be permissible. However, amendments should be at the discretion of the treating clinician in view of appreciable variability in the manifestation of such side effects.				
**Treatment-related fever, rigors, chills, and/or dyspnea**					If these symptoms occur post any dose of carfilzomib after the first cycle, a minimum dose of dexamethasone (4 mg po/iv) should be administered prior to subsequent doses of carfilzomib. In most instances it is expected that this will be the treatment dose of dexamethasone.
**Allergic reaction/ hypersensitivity**					If Grade 2-3: Hold until ≤ Grade 1, reinstitute at full dose.<br>If Grade 4: Discontinue
**Tumour lysis syndrome**					If the participant has ≥ 3 of following:<br>≥ 50% increase in creatinine, uric acid, or phosphate;<br>≥ 30% increase in potassium;<br>≥ 20% decrease in calcium;<br>or ≥ 2-fold increase in LDH

	CTD	CTDa	RCD	RCDa	CCRD
					Hold carfilzomib until all abnormalities in serum chemistries have resolved. Reinstitute at full doses.
**Neuropathy**					If Gr 2 treatment emergent neuropathy with pain or Grade 3 neuropathy: Continue to dose, if neuropathy persists for more than two weeks hold carfilzomib until resolved to ≤ Grade 2 without pain. Then restart at 1 dose decrement<br><br>Grade 4 neuropathy: Discontinue
**Hypertension including hypertensive crises**					All participants should be routinely evaluated for hypertension and treated as needed. If the hypertension cannot be controlled, the carfilzomib dose should be reduced by one dose level. In case of hypertensive crisis, stop carfilzomib until resolved or returned to baseline and consider whether to restart carfilzomib based on benefit/risk assessment.
**Pulmonary toxicity**					Evaluate and stop carfilzomib until resolved and consider whether to restart carfilzomib based on a benefit/risk assessment
**Pulmonary hypertension**					Stop carfilzomib until pulmonary hypertension has resolved or returned to baseline, and consider whether to restart carfilzomib based on a benefit/risk assessment.
**Congestive heart failure**					Dose must be held until resolution or return to baseline, after which treatment may continue at a reduced dose, or the participant may be discontinued from treatment. If no resolution after 2 weeks, the participant will be discontinued from treatment.<br><br>Any participant with symptoms of congestive heart failure (CHF) or any other suspected acute cardiac event, whether or not drug related, must have the dose held until resolution. After the event has resolved or returned to baseline, treatment may continue at a reduced dose, with the approval of the Chief Investigator, or the participant may be discontinued by treatment. If there is no resolution of CHF after 2 weeks, the participant will be discontinued from treatment.
**Other side effects (carfilzomib)**					Study drug should be held for ≥ Grade 3 events suspected to be related to carfilzomib until resolved to ≤ Grade 1 or return to baseline.

	CTD	CTDa	RCD	RCDa	CCRD
					After resolution of the event to ≤ Grade 1 or return to baseline, if the adverse event was not treatment-related, subsequent treatment with carfilzomib may resume at full dose. If the event was treatment-related, subsequent treatment with carfilzomib will resume at one level dose reduction. If toxicity continues or recurs, a 2nd carfilzomib dose reduction may be permitted at the discretion of the investigator. No more than three dose reductions will be permitted in an individual participant on study. If toxicity continues or recurs after three dose reductions, the participant should be discontinued from treatment.

Aghajanian C, Soignet S, Dizon DS, et al. (2002). A Phase I Trial of the Novel Proteosome Inhibitor PS341 in Advanced Solid Tumor Malignancies. *Clin Cancer Res:* 8: 2505-11.

Cusack JC, Jr., Liu R, Houston M, et al. (2001). Enhanced chemosensitivity to CPT-11 with proteosome inhibitor PS-341: implications for systemic nuclear factor-kappaB inhibition. *Cancer Res:* 61:3535-40.

APPENDIX I – DOSE MODIFICATIONS FOR VCD

Ha MH et al. (2003). The proteosome inhibitor PS-341 markedly enhances sensitivity of multiple myeloma tumour cells to chemotherapeutic agents. *Clin Cancer Res:* 9:1136-44.

Before each dose, participants should be evaluated for possible toxicities which may have occurred. Dose modifications will be required for the situations detailed below. Please refer to the current Summary of Product Characteristics for further details. Dose modifications and delays different from those stated in the protocol, for management of toxicities are at the discretion of the local Principal Investigator.

<table>
<tr><td>Neutropenia</td><td>If the neutrophil count falls below $0.5x10^9/L$ then the dose of cyclophosphamide can be omitted, if the count is particularly low the bortezomib therapy should be withheld until the neutrophil count rises, and a dose reduction to 1 mg/m^2 or 0.7 mg/m^2 considered for subsequent treatment cycles. An alternative option is to remain at a dose of 1.3 mg/m^2 and support the participant though the remainder of the cycle and subsequent cycles with G-CSF (e.g. Lenograstim™). This latter option may be most appropriate for participants with heavy myeloma marrow infiltration.</td></tr>
<tr><td>Thrombocytopenia</td><td>If the platelet count falls below $25x10^9/L$ then the cyclophosphamide dose can be omitted, if the counts are particularly low the bortezomib therapy should be withheld until the platelet count rises, and a dose reduction to either 1 mg/m^2 or 0.7 mg/m^2 considered for subsequent treatment cycles. An alternative option is to remain at a dose of 1.3 mg/m^2 and support the participant though the remainder of the cycle and subsequent cycles with platelets according to local guidelines. This latter option may be most appropriate for participants with heavy myeloma marrow infiltration.</td></tr>
<tr><td>Renal insufficiency</td><td>Bortezomib has not been formally studied in participants with impaired renal function, but has been given safely to participants with a reduced creatinine clearance. Participants with compromised renal function should be monitored carefully, especially if creatinine clearance is ≤30 mL/min and a dose reduction should be considered. Bortezomib has also been given to a small number of participants on dialysis where a starting does of 1 mg/m^2 is usually used.</td></tr>
<tr><td>Any Grade 3 or 4 non-haematological toxicity</td><td>Bortezomib should be withheld at the onset of any Grade 3 or Grade 4 non-haematological toxicity for up to 2 weeks until the toxicity returns to at least Grade 2. See below for neurological toxicity. Once the toxicity has recovered, the dose of bortezomib should be reduced to 1 mg/m^2 or 0.7 mg/m^2 for the remainder of the treatment courses. An alternative option is to remain at a dose of 1.3 mg/m^2 and change the treatment schedule to once per week.</td></tr>
<tr><td>Neurological toxicity (continues on to next page)</td><td>The following table contains the SPC recommended dose modifications for the management of participants who experience bortezomib-related neuropathic pain or peripheral sensory neuropathy. If the toxicity does not resolve after dosing has been withheld for 2 weeks, then the participant MUST be discontinued from treatment.
<table>
<tr><th colspan="2">Recommended dose modifications for bortezomib-related neuropathic pain and/or peripheral sensory neuropathy</th></tr>
<tr><th>Severity of peripheral neuropathy signs and symptoms</th><th>Modification of dose and regimen</th></tr>
<tr><td>Grade 1 (paresthesia and/or loss of reflexes) without pain or loss of function</td><td>No action</td></tr>
<tr><td>Grade 1 with pain or Grade 2 (interfering with function but not with activities of daily living)</td><td>Reduce bortezomib to 1 mg/m^2</td></tr>
<tr><td>Grade 2 with pain or Grade 3 (interfering with activities of daily living)</td><td>Withhold bortezomib therapy until toxicity resolves. When toxicity resolves reinitiate with a reduced dose of bortezomib at 0.7 mg/m^2 and change treatment schedule to once per week.</td></tr>
</table></td></tr>
</table>

	Grade 4 (Permanent sensory loss that interferes with function) | Discontinue bortezomib<br><br>An alternative option is to remain on bortezomib at 1.3 mg/m^2 and change the treatment schedule to once per week (days 1, 8 and 15), with the cyclophosphamide dose on the same days (i.e. 1, 8 and 15) and 20 mg of dexamethasone on the day of and day after bortezomib (i.e. days 1, 2, 8, 9, 15 and 16).<br><br>No specific prophylaxis is recommended to prevent peripheral neuropathy.

APPENDIX J – SUGGESTED DOSE REDUCTION SCHEDULE FOR LENALIDOMIDE MAINTENANCE

Lenalidomide dose level	Lenalidomide alone (10 mg starting dose)	Lenalidomide alone (25 mg starting dose)
**Starting dose**	10 mg	25 mg
**Dose level -1**	5 mg	15 mg
**Dose level -2**	5 mg every other day	10 mg
**Dose level -3**	Discontinue	5 mg
**Dose level -4**	n/a	5 mg every other day

Maintenance treatment should only be started when the neutrophil count is ≥1.0 x 10^9/L and platelets ≥100 x 10^9/L. For each subsequent cycle of treatment, lenalidomide should not be started if the Absolute Neutrophil Counts (ANC) <1.0 x 10^9/l, and/or platelet counts <75 x 10^9/l or, dependent on bone marrow infiltration by plasma cells, platelet counts <30 x 10^9/l.

Thrombocytopenia

When platelets	Lenalidomide alone (10 mg starting dose)	Lenalidomide alone (25 mg starting dose)
First fall to <30 x 10^9/L	Interrupt lenalidomide treatment	Interrupt lenalidomide treatment
Return to ≥30 x 10^9/L	Resume lenalidomide at starting dose	Resume lenalidomide at next lower dose level
For each subsequent drop below 30 x 10^9/L	Interrupt lenalidomide treatment	Interrupt lenalidomide treatment
Return to ≥30 x 10^9/L	Resume lenalidomide at next lower dose level	Resume lenalidomide at next lower dose level

Information on neutropenia on following page.

Neutropenia

When neutrophils	Lenalidomide alone (10 mg starting dose)	Lenalidomide alone (25 mg starting dose)
First fall to <1.0 x 10^9/L	Interrupt lenalidomide treatment. GCSF if Grade 3 with fever or Grade 4	Interrupt lenalidomide treatment. GCSF if Grade 3 with fever or Grade 4
Return to ≥1.0 x 10^9/L if neutropenia is the only observed toxicity	Resume lenalidomide at starting dose once daily	Resume lenalidomide at starting dose once daily
Return to ≥1.0 x 10^9/L when dose-dependent haematological toxicities other than neutropenia are observed	Resume lenalidomide at next lower dose level	Resume lenalidomide at next lower dose level
For each subsequent drop below 1.0 x 10^9/L	Interrupt lenalidomide treatment	Interrupt lenalidomide treatment
Return to ≥1.0 x 10^9/L	Resume lenalidomide at next lower dose level	Resume lenalidomide at next lower dose level (Dose Level -2, -3 and -4)

Lenalidomide is substantially excreted by the kidney, therefore care should be taken in dose selection and monitoring of renal function is advised.

No dose adjustments are required for patients with mild renal impairment. The following dose adjustments are recommended at the start of therapy for patients with moderate or severe impaired renal function or end stage renal disease.

Suggested Dose Modification for Non-Haematologic Toxicity (Maintenance schedule)

Toxicity	Lenalidomide alone (10 or 25 mg starting dose)
Non-Blistering Rash<br><br>Grade 3<br><br>Grade 4	 <br>Hold (interrupt) lenalidomide dose; follow up weekly. If the toxicity resolves to ≤ Grade 1 prior to Day 21, resume at next lower dose level (5 mg less) and continue the cycle until Day 21.<br><br>Discontinue lenalidomide study drug.
Desquamating (blistering) rash – Any Grade	Discontinue lenalidomide study drug.
Erythema multiforme ≥Grade 3	Discontinue lenalidomide study drug.
Sinus bradycardia/ other cardiac arrhythmia<br><br>Grade 2<br><br>≥ Grade 3	 <br>Hold (interrupt) lenalidomide. Follow up at least weekly. If the toxicity resolves to ≤ Grade 1 prior to Day 21, resume at next lower dose level, and continue the cycle until Day 21.<br><br>Discontinue
Allergic reaction or hypersensitivity<br><br>Grade 2 – 3<br><br>Grade 4	 <br>Hold (interrupt) lenalidomide. Follow up at least weekly. If the toxicity resolves to ≤ Grade 1 prior to Day 21, resume at next lower dose level, and continue the cycle until Day 21.<br><br>Discontinue
Venous thrombosis/embolism ≥ Grade 3	Hold (interrupt) lenalidomide dose and start anticoagulation; resume at investigator's discretion (maintain dose level).
Hyperthyroidism or hypothyroidism	Omit lenalidomide for remainder of cycle, evaluate aetiology, and initiate appropriate therapy.
Infection Grade 3 or 4	Hold lenalidomide until systemic treatment for infection is completed. If no neutropenia, resume both drugs at current dose. If neutropenic, follow neutropenic instructions.
Herpes Zoster any grade or Herpes Simplex	Hold both lenalidomide until lesions are dry. Resume at current doses

Lenalidomide is substantially excreted by the kidney, therefore care should be taken in dose selection and monitoring of renal function is advised.

No dose adjustments are required for patients with mild renal impairment. The following dose adjustments are recommended at the start of therapy for patients with moderate or severe impaired renal function or end stage renal disease.

Toxicity	Lenalidomide alone (10 or 25 mg starting dose)
Renal Dysfunction<br><br>Moderate<br>(CrCl 30-50 mL/min)<br>Severe<br>(CrCl <30 mL/min, with or without dialysis)<br><br>Serum creatinine > 2 mg/dL	 <br>10 mg once daily<br><br>Discontinue (10 mg arm)<br>15mg alternate days (25 mg arm)<br><br>Base dose reduction on CrCl as summarised above.
Grade 2 neuropathy with pain or any Grade 3 neuropathy	Hold until ≤ Grade 2. Then resume lenalidomide at reduced dose level
Grade 4 neuropathy	Discontinue
Congestive Heart Failure (CHF)	Any subject with symptoms of CHF, whether or not drug related, must have the dose held until resolution of the CHF. After the CHF has resolved or returned to baseline, treatment may continue at a reduced dose, at the discretion of the treating clinician, or the subject may be withdrawn from the study. If there is no resolution of CHF after 2 weeks, the subject will be withdrawn from protocol treatment.
Nausea, vomiting, diarrhoea, dehydration, constipation Grade ≥3 (any duration)	Hold until ≤ Grade 1. Then resume at current dose.<br><br>For each subsequent event reduce dose level
Fatigue Grade ≥3 (for any duration)	Hold until ≤ Grade 1. Then resume at current dose.<br><br>For each subsequent event reduce dose level
Elevation in transaminases (AST and/or ALT) or total bilirubin Grade 3 (for ≥ 5 days) or Grade 4 (for any duration)	Hold until ≤ Grade 1. Then resume at one reduced dose level.
Other non-haematologic toxicity assessed as lenalidomide-related ≥ Grade 3	Hold (interrupt) lenalidomide dose. Assess at least weekly. If the toxicity resolves to ≤Grade 1 prior to Day 21, resume at reduced dose level, and continue the cycle until Day 21

APPENDIX K – DEFINITION OF HIGH RISK VENOUS THROMBOSIS

It is recommended that all participants should receive thromboprophylaxis for at least the first three months of treatment. This should be done according to local guidelines. However, it is suggested that low risk participants be given aspirin (75 mg daily) and high risk patients be given LMWH. Participants any of the following risk factors are considered to be high risk.

**Risk factor**
Diabetes or other co-morbidities
Cardiovascular disease
Immobility
Prior history of thromboembolic events
Use of erythropoietic agents of other agents such as hormone replacement therapy
Renal failure

APPENDIX L – CENTRAL LABORATORY ADDRESSES

IMMUNOLOGY
University of Birmingham, Clinical Immunology Service, Division of Immunity and Infection, PO Box 1894, Vincent Drive, Edgbaston, Birmingham, B15 2SZ

THE INSTITUTE OF CANCER RESEARCH
The Institute of Cancer Research, Centre for Myeloma Research, Division of Molecular, Pathology, Brookes Lawley Building, 15 Cotswold Road, Sutton, Surrey, SM2 5NG

HAEMATOLOGICAL MALIGNANCY DIAGNOSTIC SERVICE
Level 3, Bexley Wing, St. James's Institute of Oncology, Beckett Street, Leeds LS9 7TF

APPENDIX M – EBV SUB-STUDY

Evaluation of the effect of IMiDs on EBV lifecycle in plasma samples from multiple myeloma patients in the Myeloma XI trial

Goal

To determine EBV reactivation status in plasma samples from SPM patients and associations with trial treatment.

Rationale

Lenalidomide, thalidomide, and pomalidomide are immunomodulatory agents approved for treatment of multiple myeloma. Several recent reports have raised the possibility of an increased incidence of haematological and other secondary primary malignancies (SPMs) with IMiD use. Some of these malignancies have been causally linked to Epstein-Barr virus (EBV), raising the possibility that immunomodulatory drugs may have an effect on the latent EBV lifecycle.

Myeloma XI is a trial that investigates the efficacy and safety of lenalidomide, thalidomide and proteasome inhibitors in newly diagnosed multiple myeloma patients, including an intensive treatment pathway and a non-intensive treatment pathway. The treatment scheme for the intensive pathway is illustrated in Figure 1 in this appendix. The trial has enrolled over 4000 participants in the UK. Peripheral blood samples at various time points have been collected for clinical assessments and biomarker research (Figure 2 in this appendix).

There is no clear evidence indicating an increased incidence of SPM in IMiD treated multiple myeloma (MM) patients, and no linkage between EBV activation status and the development of SPM has been established. Nevertheless, we propose to evaluate whether there is any effect of lenalidomide, either as induction or maintenance therapy, on EBV lifecycle using the blood samples from the Myeloma XI study. The effect of high dose melphalan on EBV activation will also be determined in intensive pathway patients, and compared to the effect of lenalidomide.

Figure 1: The outline of the intensive treatment pathway for Myeloma XI study

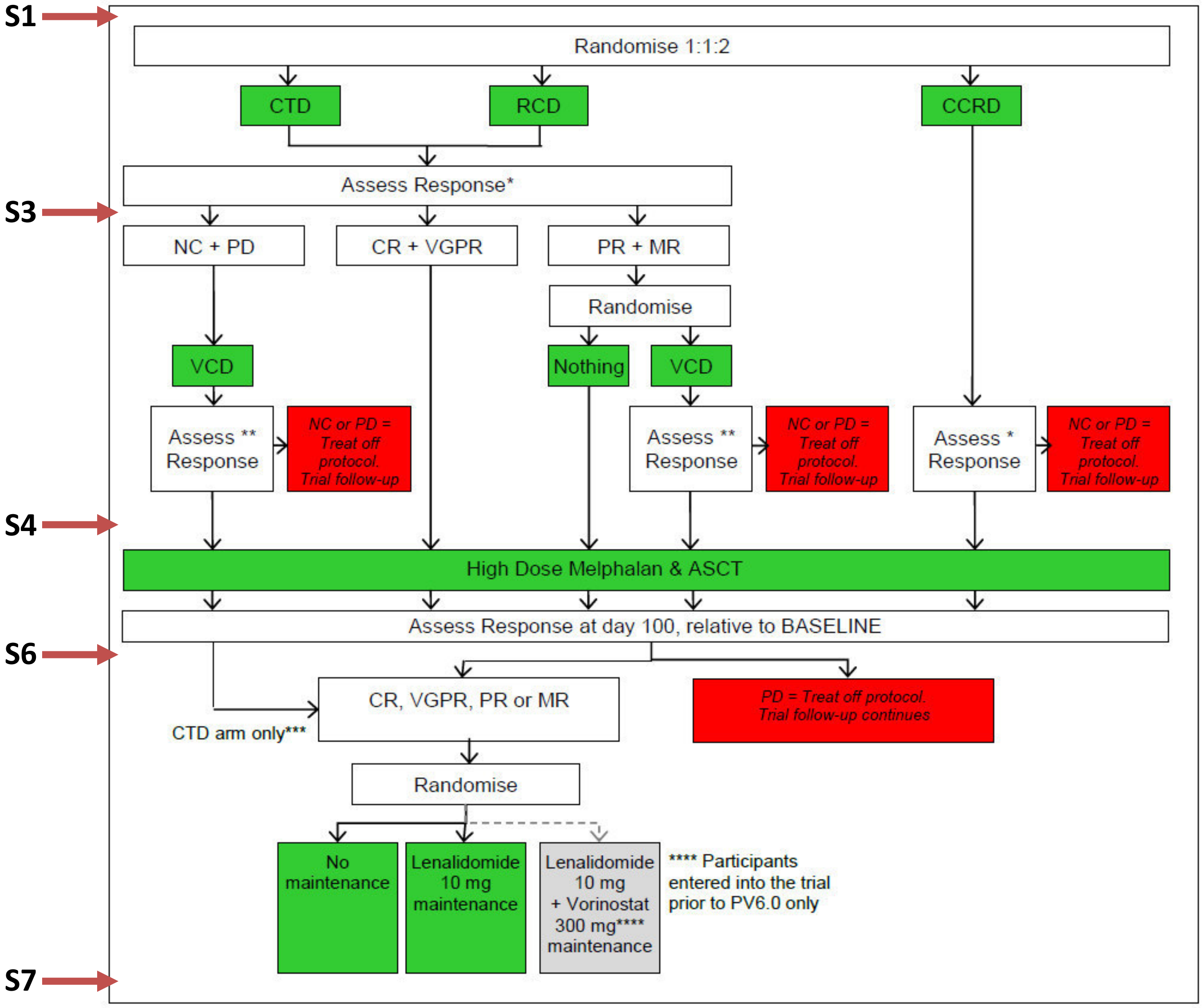

**** As of 2nd November 2017, participants receiving treatment with lenalidomide and vorinostat must permanently discontinue the vorinostat. These patients should continue to take **lenalidomide only** as maintenance treatment, as of the start of their next cycle onwards. Please see section 9.3.1.2.

Figure 2: Sample collection for central investigation in patients consented to laboratory investigation

Samples: S1 S2 S3 S4 S5 S6 S7 S8 S9

Sample	Investigation	Diagnosis (patient consented on NHS form)[1]	Trial consent	Baseline (post trial consent and before start of treatment)	Suspicion of Second primary malignancy (post treatment)[2]	Post cycle 1 and cycle 3 CTD/RCD/CCRD	Post induction treatment (CTD/RCD/CCRD)	Post VCD treatment	Stem cell harvest	1-2 weeks Post-HDT	3 months Post-HDT	2-monthly for the first 2 years & then 3-monthly to relapse	6 months post maintenance randomisation	Relapse
10 mL clotted peripheral blood Random urine sample **SEND TO BIRMINGHAM**	Creatinine, IgA, IgG, IgM, paraprotein, serum free light chain, BJP, β_2M		**Consented to Myeloma XI trial and central laboratory investigations**	✓		✓	✓	✓		✓ Plus 2 mL EDTA peripheral blood	✓	✓	✓	✓
0.5 mL of stem cell harvest (fresh, or thawed) **SEND 1ST CLASS TO BIRMINGHAM AND ICR**	T, B and NK cell subset analysis								✓					
5 mL EDTA bone marrow aspirate 3 bone marrow smears 5 mL EDTA peripheral blood **SEND TO ICR, LONDON**	Plasma cell percentage, phenotype and FISH Genomic DNA RNA expression profiling	✓ Bone marrow only		✓ Bone marrow (if not sent previously) and peripheral blood			✓ Bone marrow only	✓ Bone marrow only			✓ Bone marrow only		✓ Bone marrow only	✓
0.5 mL EDTA bone marrow aspirate **SEND TO HMDS, LEEDS**	Minimal residual disease	✓		✓ (if not sent previously)			✓	✓			✓		✓	
Tissue sample **SEND TO ICR, LONDON**	Confirmation of diagnosis				✓									

[1] Baseline bone marrow samples may be taken before trial consent, providing patients have consented to these samples being sent using the standard NHS consent form.
[2] A sample must be send to ICR upon diagnosis of a second primary malignancy

Research plan

Multiple peripheral blood samples have been collected at various phases of treatments, including the induction, stem cell transplantation, and maintenance therapy (Figure 2 in this appendix). We will conduct a staged study to investigate the effect of lenalidomide on EBV activation. We will first survey the rate of EBV positivity on blood samples, and then, if feasible, determine whether any of the specific treatments are associated with EBV activation in patients.

EBV positivity will be determined using the EBV quantitative PCR (VEBVPN artus® EBV RG PCR Kit (Qiagen)); IgG Serology EBV EBNA IgG Antibody (EBNQ); and IgM Serology EBV screen (SEBS).

Objective 1: To determine EBV reactivation status in serum samples from SPM patients

Serum samples from SPM patients at the time points described in Figure 1 in this appendix (S1, S3, S4, S6, and S7, if available) will be obtained, and the EBV load will be determined by PCR. Based on the most recent update on SPM cases in Myeloma XI trial, there are 110 confirmed SPM cases in 102 participants from Myeloma XI trial.

<u>Materials required</u>

1. 200μl of serum samples for each time points from the selected participants.
2. artus® EBV RG PCR Kit (Qiagen)

Objective 2: To determine whether lenalidomide or chemotherapy treatment is associated with EBV reactivation

Depending on the results of Objective 1, the following further work may be undertaken if feasible:

A. <u>Determine the percentage of samples that are EBV positive either at baseline or after high dose melphalan and ASCT treatment</u>

A1. 200 samples (100 each from participants who were subjected to the "No maintenance" or the "Lenalidomide 10mg maintenance" treatment; all participants should have received RCD induction therapy) in the "3 months post-HDT; S6" group will be characterized to determine the rate of EBV positivity among these samples.

A2. Similarly, 200 samples in the "baseline; S1*" group from the same participants identified in #A1 above will be surveyed to determine the rate of EBV positivity.

This information will help to determine the sample size required for the following experiments.

B. <u>Determine whether RCD induction treatment induces EBV activation</u>

The matching serum samples from the same patients selected for research plan A1 (200** samples each from the "Baseline; S1" or the "Post induction treatment; S3" group in the RCD arm) will be evaluated for EBV positivity to test whether the RCD induction therapy activate EBV in newly diagnosed MM patients.

C. <u>Determine whether high dose melphalan and ASCT treatment induces EBV activation</u>

200** matching serum samples from "Post VCD treatment; S4" will be tested for EBV positivity and compared to the samples at "3 months post –HDT; S6"that were used in Research plan #A1.

D. <u>Determine whether maintenance therapy of lenalidomide induces EBV activation</u>

200** matching serum samples at 4 and 12 months "after initiation of maintenance randomisation; S7" will be investigated for EBV positivity, and compared to the samples at "3 months post –HDT; S6" that were used in research plan #A1.

** S1, 2, 3, 4, 5, 6, 7 refers to the samples collected at various time points of the study, as indicated in both Figure 1 and Figure 2 of this Appendix.*

*** The exact number of samples to be used in Research Plan B, C, & D may be adjusted after analysing the data generated from Research Plan #A1 and #A2 and sample size calculation undertaken.*

Summary of Myeloma XI protocol amendments

Myeloma XI opened to recruitment under protocol v2.0, dated 26th August 2009

Protocol version number & date	Main amendments made to previous version of protocol
Version 3.0, 7th December 2010	Main rationale for protocol amendment: To update information about the central lab samples.<br>Amendments from protocol v2.0-v3.0:<br>1. Change to the frequency of follow-up clinic visits until disease progression (Section 10.2.1: Local follow-up)<br>Previous wording: Patients should attend clinic for follow up visits every 2 months until disease progression<br>New wording: Patients should attend clinic for follow up visits every 2 months for the first 2 years and 3 monthly thereafter until disease progression.<br>2. Patient names required on samples sent to the central labs (Section 17.4: Confidentiality)<br>Samples sent to central laboratories will require patient names to be included on the samples in order for the samples to be correctly identified and processed up on receipt in line with the minimal identifiable information required by the laboratories. Results can then be reported back to the treating physician. This is clarified on the participant information sheet.<br>3. Change in the bone marrow samples sent to the central labs (Section 10.1: Baseline investigations, Section 10.2: Follow-up investigations, and Appendix G)<br>Removal of bone marrow trephine samples for central analysis, as they were not required.
Version 4.0, 5th April 2011	Main rationale for protocol amendment: To clarify information about the central lab samples.<br>Amendments from protocol v3.0-v4.0:<br>1. Clarifying text in central investigations table (Appendix G)<br>To bring central investigations table in line with protocol text.<br>2. Clarification on sending bone marrow samples for central review (Section 10.1: Baseline investigations)<br>Amending text to clarify that only the bone marrow samples should be sent to central review at the same time as local diagnostic

	procedures.<br><br>3. <u>Clarifying when serum free light chains should be analysed (Section 10.1: Baseline investigations)</u><br>Clarification that that serum free light chain analysis should be performed as part of the local investigations at presentation.
Version 5.0, 14th September 2011	Main rationale for protocol amendment: To add a new treatment arm in the maintenance randomisation.<br><br>Amendments from protocol v4.0-v5.0:<br><br>1. <u>Addition of a new treatment arm in the maintenance randomisation</u><br>Addition of a 3rd treatment arm in the maintenance randomization (lenalidomide plus vorinostat). Rationale: New data from other studies has demonstrated an important clinical benefit (clear and significant improvement in progression free survival) for the use of maintenance lenalidomide in newly diagnosed myeloma in both younger and older patients. Thus for the trial to remain timely, the maintenance randomisation has been changed to include a 3rd arm of lenalidomide in combination with vorinostat. There remains uncertainty surrounding the impact of lenalidomide maintenance on overall survival, therefore there is sound rationale for retaining a control arm.<br>Amended sections in protocol: Title, summary, flow diagrams, Section 1: Background, Section 2: Aims and objectives, Section 3: Trial design, Section 4.6: Exclusion, Section 7: Randomisation, Section 8: IMPs, Section 9: Study treatment, Sections 14.1.5, 15.1, and 15.2 (Statistical considerations, maintenance, interim analysis, respectively), Section 20: References, Appendix I: Dose reduction schedule for lenalidomide +/- vorinostat maintenance).<br><br>2. <u>Changes in lenalidomide maintenance dosing (Section 9: Study treatment)</u><br>Change of lenalidomide maintenance dose from 25 mg daily to 10 mg daily. (Schedule of administration unchanged). Dose reduced due to recent published data showing the effectiveness of the 10 mg dose and the potential to reduce toxicity.<br><br>3. <u>Clarification of exclusion criteria (Sections 4.2, 4.4, and 4.6: Exclusions for initial, consolidation and maintenance randomisations, respectively)</u><br>The following updates were made to the exclusion criteria for clarification:<br>- To clarify previous malignancy exclusions<br>- Addition of MDS as an exclusion<br>- Clarification that lactating and breastfeeding women are excluded<br>- Patients who have not responded to any protocol treatment are not eligible for maintenance randomisation<br>4. <u>Pharmacovigilance updates (Section 11.1.3: ARs, Section 11.2: Operational definition and reporting adverse events and reactions,</u>

	Section 11.3.1: Events not classed as SAEs, Section 11.3.2: Expected SAEs)<br>The following updates were made to provide clarification:<br>- Adverse reaction definition added<br>- Hospitalisation for disease progression, deaths due to myeloma are not reportable as SAEs<br>- Clarification of the reporting timelines for ARs (up until 30 days after protocol treatment).<br>- Addition of expected SAEs for vorinostat<br><br>5. Clarifying requirements for response assessment (Section 10.2.1: Local investigations, Sections 10.2.3 and 10.2.4: Timing of samples for central investigations, Section 10.2.6: Response and relapse assessment, Section 12: Criteria of response, Section 20: References, Appendix C: Definitions of response, Appendix E: Local and central investigations)<br>The following updates were made to clarify the requirements for response assessment (as per the uniform response criteria):<br>- All response categories require 2 consecutive assessments made at any time before the institution of any new therapy<br>- Tests must include immunofixation<br>- A bone marrow is required to confirm CR (2 consecutive assessments not needed)<br>- Criteria for light chain only patients added<br>- Definition of maximum response added for guidance (not part of the Uniform response criteria)<br>- Paraprotein responses should only be calculated using sequential paraprotein measurements made in the same laboratory using the same method.<br><br>6. Updates to central samples (Sections 10.2.3 and 10.2.4: Timing of samples for central investigations, Appendix E: Local and central investigations)<br>The following updates were made to the central samples:<br>- Additional central sample collection at end of cycle 3<br>- Addition of a 6 months post randomisation time point: Blood and urine samples to the Birmingham myeloma lab, and bone marrow to the ICR and HMDS labs<br>- Change in the volume of the baseline bone marrow sample sent to HMDS : 0.5ml instead of 2ml<br>- Addition of text to clarify that bone marrow samples at post-induction, post-VCD and 3 months post HDT should be split between ICR in addition to HMDS<br>- Addition of text to clarify that a stem cell harvest sample should be sent to ICR in addition to HMDS. Samples can be sent fresh or

	thawed by first class post.<br><br>7. <u>Clarification on use of zoledronic acid (Section 1.6.1: Introduction – supportive care, bisphosphonates, Section 1.8.3 Summary and rationale for trial, Section 9.4: Supportive measures</u><br>Text added to suggest that patients should be treated with zoledronic acid, but this is not a prerequisite to a patient being included in the study.<br><br>8. <u>Addition of advice about entering TEAMM trial (Section 9.4: Supportive measures)</u><br>Addition of advice to clarify that patients are eligible to enter the TEAMM trial (a randomised placebo controlled trial of levofloxacin once daily for the first 12 weeks from diagnosis).
Version 6.0, 28th June 2013	Main rationale for protocol amendment: To add a 3rd treatment arm in the induction randomisation, and to remove one treatment arm in the maintenance randomisation.<br><br>Amendments made to protocol v5.0-v6.0:<br><br>1. <u>Addition of a 3rd treatment arm in the induction randomisation in the intensive pathway of the trial</u><br>Addition of a 3rd treatment arm (carfilzomib, cyclophosphamide, lenalidomide and dexamethasone) in the induction randomisation for the intensive pathway. In this group the randomisation will be that 50% of participants will receive the new 4 drug combination while the other 50% will follow the original study randomisation to either CTD or CRD. The induction pathway for the non-intensive arm was not changed. Recruitment was continued to enable approximately a further 2000 patients to be entered into the trial. Rationale: New data from other studies has demonstrated an important clinical benefit with the use of a concomitant proteasome/IMiD combination. Thus for the trial to remain timely, the induction randomisation has been changed to include a 3rd arm of CCRD (alkylating agent, steroid, IMiD and proteasome inhibitor).<br>Amended sections: Title, summary, flow diagrams, Section 1: Background, Section 2: Aims and objectives, Section 3: (Trial design, Section 4: Exclusion, Section 7: Randomisation, Section 8: IMPs, Section 9: Study treatment, Section 10: Laboratory investigations and data collection, Section 14: Statistical considerations, Section 15: Statistical analysis, Section 20: References, Appendix E: Local investigations and sample collection for central investigation, Appendix J: Dose reduction steps for carfilzomib<br><br>2. <u>Removal of one treatment arm in maintenance randomisation</u><br>The lenalidomide plus vorinostat maintenance treatment was removed for new participants. Participants who entered the trial prior to PV6.0 were still be eligible to be randomised to receive lenalidomide plus vorinostat maintenance treatment. Rationale: Sufficient patients had been recruited to complete the lenalidomide plus vorinostat maintenance treatment part of the trial.<br>Amended sections: Title, summary, flow diagrams, Section 2: Aims and objectives, Section 3: Trial design, Section 7: Randomisation,

	Section 9: Study treatment, Section 14: Statistical considerations<br><br>3. Updates to exclusion criteria (Section 4.2: Exclusion criteria for initial randomisation, and Section 4.4: Exclusion criteria for VCD randomisation)<br>The following changes were made to the exclusion criteria for clarification:<br>- Clarification of previous corticosteroid use;<br>- Clarification that participants who have received CCRD are excluded from VCD randomisation.<br><br>4. Update to recruitment figures (Section 5.1: Recruitment, Section 14: Statistical considerations, Section 15: Statistical analysis)<br>Recruitment figures updated in line with the extension to trial recruitment.<br><br>5. Clarification about baseline investigations (Section 6: Baseline investigations, Section 10: Laboratory investigations and data collection, Appendix E: Local investigations and sample collection for central investigation)<br>Clarification that baseline investigations should be performed prior to Myeloma XI treatment, except for the axial skeletal survey and local investigations which need to be completed prior to randomisation. Removal of the optional CT/MRI scans. Clarification that baseline disease assessments (paraprotein, serum free light chains and urinary light chains) should be from when paraprotein / light chain values are at their highest.<br><br>6. Clarification about bortezomib administration (Section 8: IMPs, Section 9: Study treatment)<br>Text added to clarify that bortezomib may be given subcutaneously. Rationale: Recent data had shown that bortezomib is suitable for subcutaneous as well as intravenous administration.<br><br>7. Central samples (Section 10: Laboratory investigations and data collection, Appendix E: Local investigations and sample collection for central investigation)<br>Text added to clarify that upon suspicion of a second primary malignancy a sample be sent to the ICR lab to confirm diagnosis and further research purposes.<br><br>8. Response assessments (Section 10: Laboratory investigations and data collection, Section 11: Pharmacovigilance procedures)<br>Clarification that wherever possible, local response assessments should take into consideration the results provided by the central laboratories.<br><br>Text added to clarify the following requirements for response assessment (as per the uniform response criteria) - Appendix C

	(Definitions of response):<br>- Confirmation of progression disease for light chain only patients requires 2 consecutive assessments<br>- Clarification of the bone marrow plasma cell measurements for PD<br><br>9. Pharmacovigilance updates (Section 11.2 Operational definition and reporting adverse events and reactions, Section 11.3.2: Expected SAEs, Section 11.3.3: Recording and reporting SAEs and SUSARs, Section 11.4: Pregnancies or suspected pregnancies, Section 11.5: Responsibilities<br>The following changes were made to the pharmacovigilance section:<br>- SAEs, SARs and SUSARs are reportable for the duration of the trial<br>- Inclusion that second primary malignancies are an expected SAE for lenalidomide<br>- Inclusion that tumour lysis syndrome is an expected SAE for carfilzomib<br>- Reporting procedure for second primary malignancies<br>- Clarification of reporting procedures for pregnancies<br>- Clarification that it is CTRU responsibility to report SAEs to the relevant drug companies involved in the trial<br><br>10. Endpoints (Section 13: Endpoints)<br>The following changes were made to the endpoints:<br>- Clarification that the secondary end point of response includes CR rate at the end of induction<br>- Inclusion of lenalidomide and vorinostat to the definition of overall survival for maintenance therapy
Version 6.3, 11th December 2014	Main rationale for protocol amendment: To update the eligibility criteria.<br>Amendments made to protocol v6.0-v6.3:<br>1. Changes to eligibility criteria (Section 4: Eligibility):<br>- Addition of the following text in the inclusion criteria for initial randomisation and VCD randomisation, regarding contraception advice: "Contraception must be used during treatment and for 3 months following bortezomib or cyclophosphamide treatment". Updated in line with trial IMP SPCs/IBs information.<br>- Deletion of text regarding malignancies to exclude all previous or concurrent malignancies in the following eligibility criteria: exclusion criteria for initial randomisation, exclusion criteria for randomisation to VCD, and exclusion criteria for maintenance randomisation. Updated in line with Trial Management Team decision to exclude all previous or concurrent malignancies due to the

	potential increased risk of second malignancy in participants treated with lenalidomide.<br>- To exclude all MDS cases, deletion of text regarding documented diagnosis of Myelodysplastic Syndrome (MDS) having to meet the criteria for high risk disease in the following eligibility criteria: exclusion criteria for initial randomisation, exclusion criteria for randomisation to VCD, and exclusion criteria for maintenance randomisation. Updated in line with Trial Management Team decision to exclude all previous or concurrent malignancies, due to the potential increased risk of second malignancy in participants treated with lenalidomide.<br><br>2. <u>Updates to carfilzomib administration and dose capping (Section 8: IMPs, Section 9: Study treatment)</u><br>Addition of text to carfilzomib administration information that pre-carfilzomib dexamethasone 4 mg po/iv not necessary if already had 40 mg po (or lower dose if reduced due to toxicity), and that full protocol defined treatment dose of dexamethasone should be given in addition to this (post carfilzomib infusion). Also added that an alternative corticosteroid, being given due to toxicity, can be used at an equivalent dose prior to carfilzomib infusion. Addition of carfilzomib dose capping information.<br><br>3. <u>Clarification on platelet count requirements (Section 9: Study treatment)</u><br>Change to platelet count requirement in criteria for starting each cycle of trial treatment following treatment dose reduction.<br>In line with SPC/IB.<br><br>4. <u>Clarification on skeletal survey requirements (Section 10.1: Baseline investigations, Appendix F: Axial skeletal survey)</u><br>Addition of text to clarify that unenhanced whole body CT can be undertaken in place of skeletal survey if this is local policy. This text was added to reflect variable local practices and following discussion with Chief Investigator.<br><br>5. <u>Clarification of haematological assessments (Section 10.2: Follow-up investigations)</u><br>Deletion of text specifying participants receiving *lenalidomide* may receive more frequent haematological assessments, which are required according to all trial IMP SPCs. Rationale: For clarity – so this applies to all IMPs and not just lenalidomide.<br><br>6. <u>Clarification of biopsy sample requirements (Section 10.2: Follow-up investigations)</u><br>Deletion of requirement for a biopsy sample during follow-up investigations upon suspicion of a secondary primary malignancy - samples no longer required to be sent centrally, because local analysis is adequate.<br><br>7. <u>Clarification of SAE definition (Section 11.1.2: Serious Adverse Events (SAEs))</u><br>Definition of SAE amended to include "other important medical event" and deletion of any effect which "may jeopardise the participant and may require medical or surgical intervention to prevent one of the other defined outcomes". Rationale: To bring the protocol in line

	with the standard SAE definition.<br><br>8. Reporting pregnancies and suspected pregnancies (Section 11.4: Pregnancies or suspected pregnancies):<br>Time-frame for reporting pregnancies and suspected pregnancies added as 3 months after the cessation of trial treatment, to bring this in line with SPCs.<br><br>9. Reporting suspected Second Primary Malignancies (SPMs) (Section 11.3.3 Recording and reporting SAEs and SUSARs)<br>Addition of suspected SPMs in the section on recording and reporting of SAEs and SUSARs, and addition of requirement to report on both the SPM and SAE CRF.<br><br>10. Dose reduction schedules (Appendix I: Suggested dose reduction schedule for lenalidomide maintenance and lenalidomide with vorinostat maintenance)<br>Clarification added for Absolute Neutrophil Counts (ANC) and platelet values with regard to dose reduction schedule for lenalidomide containing schedules, to bring the protocol in line with SPCs.<br><br>11. Contraception<br>The following sections containing contraception advice were clarified:<br>- Contraception information for women updated in line with IMP SPCs: contraceptive measures must be used for 3 months after completion of treatment (rather than 1 month).<br>- Contraception information for men with females partners of childbearing potential amended to clarify contraceptive methods: a) must be used (rather than 'should'), b) to be used 'during' chemotherapy made explicit, c) updated in line with IMP SPCs that they must be used for 3 months after completion of treatment (rather than 1 month).<br>- Deletion of reference to Lenalidomide and Thalidomide and replaced with 'chemotherapy' or participation in Myeloma XI trial to encompass all trial IMPs, with regards to use of contraception.<br>- Addition of statement of the potential risks for unborn children by other drugs used in the trial (as well as thalidomide and lenalidomide) and the requirement for female participants to use two methods of contraception.
Version 6.4, 22nd January 2015	Amendments made to protocol v6.3-v6.4:<br><br>Non-substantial amendments to correct typos and grammatical errors in protocol v6.3 – this did not need REC approval.
Version 7.0, 26th January 2016	Main rationale for protocol amendment: To update the sections on statistics in line with the rolling nature of the trial design.

	Amendments made to protocol v6.4-v7.0:<br><br>1. <u>Clarification of text in further translational objectives (Section 2.4: Further translational objectives)</u><br>The following updates were made in this section:<br>- Addition of text to include the identification of new abnormalities using modern scientific techniques.<br>- Deletion of text with regards to predictive classification of venous thromboembolic events exposure associated with IMiDs.<br><br>1. <u>Updates to eligibility criteria (Section 4: Eligibility)</u><br>The following updates were made to the eligibility criteria:<br>- Addition of text to the eligibility section to explain that eligibility waivers to the inclusion/exclusion criteria are not permitted.<br>- Addition of text to the inclusion criteria for initial randomisation to clarify information about abstinence and contraception.<br>- Removal of the following text in the exclusion criteria for initial randomisation: "Caution is advised in participants with a past medical history of ischaemic heart disease..." - added as a guidance note instead in the same section, below the list of exclusion criteria.<br>- Addition of new exclusion criterion: "Patient has active or prior hepatitis C". Rationale: New criterion identified by the Trial Management Group committee to clarify eligibility requirements, following multiple queries from sites.<br>- Clarification of text in the inclusion criteria for maintenance randomisation to define completed randomised induction treatment.<br><br>2. <u>Clarification of informed consent process (Section 5.2: Informed consent process)</u><br>Addition of text in the section describing the informed consent process to inform participants that an invitation to participate in the trial will be made during their first consultation, or at the time they receive their diagnostic test results.<br><br>3. <u>Carfilzomib information (Section 8.1.4: IMP administration)</u><br>Addition of text relating to cardiac failure and hypertension events for carfilzomib in the section on IMP administration. Rationale: Updated carfilzomib related safety information - patient receiving carfilzomib must be monitored for cardiac and hypertension events.<br><br>4. <u>Clarification of timelines for maintenance randomisation (Section 9.3: Maintenance)</u><br>Addition of text to the section on maintenance randomisations to explain that eligible participants must be randomised to maintenance arm within 9 months of completing induction or ASCT. All investigations listed in the appendices were also updated to reflect this.

	5. Clarification of the definition of end of trial (Section 10.3: Definition of end of trial)<br>Addition of text in the section describing the definition of end of trial to explain that participants are to be followed up until death or until the final analysis of survival data as described in Section 15 of the protocol.<br><br>6. Updates to protocol sections on statistics (Section 14: Statistical considerations, Section 15: Statistical analysis, Section 16: Data monitoring)<br>The following updates were made to the sections on statistics:<br>- Text re-written in Section 14 (Statistical Considerations, Section 15 (Statistical Analysis), and Section 16 (Data Monitoring) to reflect the rolling nature of the design and information added about when each endpoint and analysis is expected to be reached.<br>- Addition of PFS2 as a secondary endpoint.<br><br>7. Updates to Second Primary Malignancy (SPM) samples (Appendix E: Local investigations and sample collection for central investigation)<br>Clarification of text in the intensive and non-intensive pathway tables in Appendix E to explain that an SPM sample is only required if a participant is diagnosed with a second haematological malignancy. Footnote 3 deleted from both intensive and non-intensive pathway tables as a bone marrow sample is no longer required to be taken outside of the time points noted.<br><br>8. Updates to dose modifications (Appendix H: Dose modifications for induction regimens)<br>Dose modification for induction regimens table introduced in Appendix H and "recommended action carfilzomib" added in relation to safety update (#3) from Amgen/Onyx with regards to Posterior Reversible Encephalopathy Syndrome (PRES) – in relation to participants receiving carfilzomib.
Version 8.0, 29th September 2016	Main rationale for protocol amendment: To add information about a new laboratory sub-study to the protocol.<br><br>Amendments made to protocol v7.0-v8.0:<br><br>1. Addition of a new laboratory sub-study<br>Information about a new laboratory sub-study added to the following sections:<br>- Addition of the laboratory sub-study objective in Section 2.3: To determine EBV reactivation status in plasma samples from SPM patients and associations with protocol treatment.<br>- Addition of a new appendix (Appendix M) to describe the sub-study proposal.<br><br>2. Clarification of time point for central follow up

<table>
<tr>
<td></td>
<td>In Section 10.2.2, clarification of one time point for central follow-up; "treatment response" replaced with "disease progression"

3. <u>Reference safety information</u>
Update of table in Section 11.3.2 to clarify that the Carfilzomib Investigator's Brochure v16.1 to be used as Reference Safety Information.</td>
</tr>
<tr>
<td>Version 9.0, 2nd November 2017</td>
<td>Main rationale for protocol amendment: To update information about discontinuing vorinostat, following an urgent safety measure.

Amendments made to protocol v8.0-v9.0:

1. <u>Updates to Section 9.3.1.2</u> (Lenalidomide Vorinostat (RZ) maintenance (for participants entered into the trial prior to protocol version 6.0 only))
Addition of the following text in red:

As of 2nd November 2017, participants receiving treatment with lenalidomide and vorinostat must permanently discontinue the vorinostat. These patients should continue to take **lenalidomide only as maintenance treatment, as of the start of their next cycle onwards. They should continue taking lenalidomide only up until disease progression, in the absence of toxicity.**
<table>
<tr><td>Days 1-21</td><td>Lenalidomide 10 mg daily po</td></tr>
<tr><td>Days 1-7 and 15-21</td><td>AS OF 2ND NOVEMBER 2017, ALL PARTICIPANTS RANDOMISED TO ~~LEN+VORST~~ LENALIDOMIDE AND VORINOSTAT MUST PERMANENTLY DISCONTINUE VORINOSTAT TREATMENT ON COMPLETION OF THEIR CURRENT TREATMENT CYCLE. SEE ABOVE**</td></tr>
</table>
2. <u>Dose reduction schedules</u>
The dose reduction schedule (Appendix J) was amended to remove dose reduction advice for vorinostat, but to continue to advise on single agent lenalidomide.

3. <u>Addition of references to discontinuing vorinostat throughout the protocol:</u>
Addition of the text below to the following pages in the protocol: 9, 10, 35, 37, 41, 42, 47.
"NB: See Section 9.3.1.2 (page 50) for ongoing treatment details of participants randomised to the lenalidomide + vorinostat arm".

4. <u>Updates to trial flow diagrams to reflect discontinuation of vorinostat</u>
Addition of the text below to the Pathway Outline flow diagrams (in the Study Summary and in Sections 9.1.1 and 9.2.1, and Appendix M):
"**** As of 2nd November 2017, participants receiving treatment with lenalidomide and vorinostat must permanently discontinue the</td>
</tr>
</table>

	vorinostat. These patients should continue to take **lenalidomide only** as maintenance treatment, as of the start of their next cycle onwards. Please see section 9.3.1.2”.
