# Supplementary material for: Carfilzomib, lenalidomide, dexamethasone, and cyclophosphamide (KRdc) as induction therapy for transplant-eligible, newly diagnosed multiple myeloma patients (Myeloma XI+): Interim analysis of an open-label randomised controlled trial
Source: PLoS Med. 2021 Jan 11;18(1):e1003454. doi: 10.1371/journal.pmed.1003454 (PMC7799846; doi:10.1371/journal.pmed.1003454)
Supplement: Attachment — Submitted filename: KRdc responses 080920.docx [file pmed.1003454.s002.docx]

Richard Turner, PhD
Senior Editor, PLOS Medicine

Dear Dr Turner,

**Re: PMEDICINE-D-20-00730R1**

We would like to thank you and the reviewers for your consideration of our manuscript.

We are grateful for the insightful comments which have helped us improve our manuscript in the revised version attached.

We have submitted one "clean" copy and one copy where the changes are tracked, using Word's tracked changes function. Please find below a list of the editorial and referee comments with our replies shown in purple below each point. Where indicated, page numbers refer to those in the tracked changes version of the document for ease of reference.

We hope that with these changes you will find our manuscript suitable for publication in PLOS Medicine and would be happy to answer any further queries.

Yours sincerely,

Professor Graham Jackson

**Editorial comments:**

Please review PLOS Medicine's data policy (<https://journals.plos.org/plosmedicine/s/data-availability>) which your paper will need to comply with in the event of publication. For example, a non-author contact would need to be provided for those inquiring about access to study data.

The data sharing statement has been amended and we have added a contact for a non-author at the trials centre via whom data access can be requested. It now reads (Page 19):

“De-identified participant data will be made available when all trial primary and secondary endpoints have been met. Any requests for trial data and supporting material (data dictionary, protocol, and statistical analysis plan) should be sent to ctru-dataaccess@leeds.ac.uk in the first instance. Only requests that have a methodologically sound proposal and where use of the data has been approved by the independent trial steering committee will be considered. Data requestors will need to sign a data access agreement.”

Please add a study descriptor to the title, e.g., "...myeloma: an open-label randomized controlled trial".

This has been added. The title is now: “Carfilzomib, Lenalidomide, Dexamethasone and Cyclophosphamide (KRdc) as Induction Therapy for Transplant Eligible Newly Diagnosed Multiple Myeloma Patients: Myeloma XI+, an open-label randomised controlled trial.”

Please combine the "methods" and "findings" subsections of your abstract to match journal style. The final sentence of the new combined subsection should quote 2-3 of the study's main limitations.

These have been combined and the following sentence added to the end of the Methods and Findings subsection (Page 3):

“Limitations of the study include a lack of blinding to treatment regimen and that the triplet control regimen did not include a proteasome inhibitor for all patients, which would be considered a current standard of care in many parts of the world.”

Please add summary demographic details for study participants to the abstract.

These have been added:

“1056 patients (median age 61 years, range 33 to 75, 39.1% female)”

After the abstract, we will need to ask you to add a new and accessible "author summary" section in non-identical prose. You may find it helpful to consult one or two recent research papers in PLOS Medicine to get a sense of the preferred style.

This has been added.

Please remove the "role of the funding source" section from the text. This information will appear in the article metadata in the event of publication, via entries in the submission form. Similarly, please remove the conflict of interest information from the end of the main text.

These have been removed.

Please avoid claims of "the first" (e.g., at the start of the discussion section) and where necessary add "to our knowledge" or similar.

This has been amended.

Please restructure the discussion section so that there is a discrete paragraph summarizing study limitations.

The penultimate paragraph of the manuscript is dedicated to summarizing the key study limitations and has been expanded.

Should that be "these data" on p.14?

This has been amended (now page 18).

Please substitute "sex" for "gender" where appropriate, e.g., in figure 1b.

This has been amended in Figure 1B, we apologise for this oversight.

Throughout the paper, please quote exact p values or p<0.001.

These have been amended.

Please aim to be consistent about phase 3/III; and US/UK spelling (e.g., "haematological"/"multi-center").

We have unified style throughout the manuscript.

Throughout the text, please adapt reference call-outs as follows: "... are published [8,9]." (i.e. preceding punctuation and with no spaces within the square brackets).

In the reference list, please ensure that journal names are abbreviated consistently; and where appropriate list 6 author names rather than 3, followed by "et al.".

We have changed the reference formatting to PLOS endnote style.

Please move the patient flowchart to the main text.

The patient flowchart is now Figure 1.

Please add a completed CONSORT checklist as an attached file, referred to in the methods section of the main text. In the checklist, individual items should be referred to by section (e.g., "Methods") and paragraph number rather than by line or page numbers, as the latter generally change in the event of publication.

A CONSORT checklist has been attached and referred to in the methods section. In a few instances this prompted additions to the manuscript as marked.

***** Reviewer #1:**

We thank Prof McConnachie for his insightful review of our manuscript and are pleased he found it to be reporting an impressive trial and worthy of publication. We have aimed to answer all his helpful suggestions in full to aid the presentation and understanding of our data.

Alex McConnachie, Statistical Review

This is a review of the statistical aspects of Jackson et al's paper about the interim results of the Myeloma XI+ trial, comparing progression free survival between patients treated with a 4-drug combination versus one of two 3-drug combinations.

This is a hugely impressive trial, and I am in favour of the decision to seek to publish the results of the study to date. The statistical analyses are generally very good, and my comments are mainly to do with the way that things have been presented.

The co-primary endpoints are PFS and overall survival, though this paper does not report the OS data. This is fine, but reading the abstract, the reader is expecting to see them. The results in the abstract show PFS results, and then moves on to response rates and adverse events, giving the impression that the OS data are being omitted. I think the first part of the abstract, and maybe the paper as a whole (including the title?), could be more up-front about this being a planned interim analysis, focusing on the first of two co-primary outcomes, plus secondary and safety outcomes. This is clearer on reading the statistical methods section, but many readers will not get that far.

We have amended both the abstract and the methods to make it clear that this is a planned interim analysis and does not include overall survival data, for which follow up is ongoing. The title is already quite long so we have not added to the title. We have however added details to the Abstract, Methods and Findings (Page 2):

“PFS, response and safety outcomes are reported following a planned interim analysis.”

and

“The trial continues to treat and follow-up patients to the co-primary endpoint of overall survival and for planned long-term follow-up analysis.”

Methods, Study design and participants (Page 6):

“This planned interim analysis, reports the first of two co-primary outcomes, progression-free survival, and secondary and safety outcomes.”

The previous description within the Methods, Statistical analysis section remains the same.

The wording in the abstract "…median PFS not reached vs 36.2 months" is not immediately clear. Perhaps a few more words need to be used to explain this a little better, for someone who is only reading the abstract.

This sentence has been expanded to clarify. It now reads (Page 2):

“After a median follow up of 34.5 months KRdc was associated with a significantly longer PFS than the triplet control group (hazard ratio 0.63, [95%CI 0.51-0.76]. The median PFS for patients receiving KRdc has not yet been reached vs 36.2 months for the triplet control group, P<0.001).”

The unblinded nature of the trial is not mentioned as a limitation. Should it be?

The unblinded nature of the trial has been added to the paragraph about limitations of the study and to the sentence about limitations in the abstract.

Abstract, Methods and findings (Page 3):

“Limitations of the study include a lack of blinding to treatment regimen and that the triplet control regimen did not include a proteasome inhibitor for all patients, which would be considered a current standard of care in many parts of the world.”

Discussion (Page 18):

“Another limitation includes the unblinded nature of the therapy randomisation, both the patient and local investigator were aware of the treatment being delivered.”

Particularly as a non-clinical reader, I found the use of abbreviations quite challenging at times throughout the paper. Often they were not spelled out in full on first use, making it difficult to follow.

We apologise for the lack of clarity around some of the abbreviations. We have gone through and made sure that all abbreviations are spelled out at first use and tried to limit their use where possible. We have simplified the drug combination abbreviations to aid understanding.

The primary analysis method of an adjusted Cox model is fine. I presume the proportional hazards assumption was checked, and was met, though this is not mentioned.

The proportional hazards assumption was assessed informally by plotting the hazards over time (i.e., the log cumulative hazard plot) for each treatment group and formally using the simulation-based method of Lin et al. These checks provided no evidence of the proportional hazards assumption being violated. This has been added to the methods and results.

A subgroup analysis is presented of the main treatment effect within those who were MRD negative (though I had to look up what MRD means). Is this a sensible analysis, given that MRD status is defined after randomisation (and treatment)? Should the same results from the MRD+ group be reported? Or, should the main analysis be performed with adjustment for MRD status, to assess whether MRD status explains the treatment effect?

Minimal residual disease (MRD) is currently being evaluated by the FDA as a proposed surrogate outcome for PFS in myeloma studies. Therefore we consider the reporting the MRD status after the end of treatment, although it is post-randomisation, to be of interest to the community. We agree that it is somewhat biased to have not reported the figures for the MRD+ group and have now added this to the text. We did not mean to imply that any formal testing in a subgroup had taken place and have revised the term ‘significantly’ used in the text. A manuscript which describes in detail the impact of this potential surrogate outcome on the trial primary outcomes is in preparation, and will include such analysis as the reviewer describes.

The section on MRD in the results now reads (Page 13):

At the end of induction MRD negativity (MRD-) was achieved in more patients in the KRdc group (55% [90/164]) compared to (17% [26/157]) in the control group (11% [8/74] with CTD, 22% [18/83] with CRD). PFS for MRD- patients was improved compared to MRD+ patients (% PFS at 3yrs from MRD assessment, MRD- 78.7% vs MRD+ 51.9%). Interestingly there were differences in outcome within the MRD- group with patients receiving KRdc doing better (% PFS at 3yrs from MRD assessment, KRdc MRD- 81.8% vs control MRD- 68.1% (CTD 62.5% and CRD 70.6%). In the MRD+ group the outcomes were similar (% PFS at 3yrs from MRD assessment, KRdc MRD+ 49.9% vs control MRD+ 52.8% (CTD 61.8% and CRD 40.7%). At 100 days after ASCT the rate of MRD- had improved in all groups, but remained higher in the KRdc group (75% [152/202]) compared to (50% [80/160]) in the control group (51% [40/79] with CTD, 49% [40/81] with CRD). The achievement of MRD- status after ASCT was also associated with improved outcomes.

The final efficacy result reported is that KRdc showed improved PSF2 compared to CTD, but not CRD, when analysed separately. Given that the KRdc vs. CTD/CRD comparison only just achieved p<0.05, it is almost certain that at least one of KRdc vs. CTD or CRD will not achieve this p-value threshold. Is this observation worthy of a mention, when I doubt that there is any evidence of a difference between CRT and CRD?

Conversely there is actually evidence of a difference in PFS, PFS2 and OS outcome between patients treated with CTD and CRD in a larger group of patients recruited to Myeloma XI and reported in Jackson et al, Haematologica, 2020.

The contemporaneous randomisation reported in this manuscript was not powered to show a difference between CTD and CRD (or indeed between KRdc and either one, but rather the combined triplet control). We would therefore prefer not to add any further significance tests to the manuscript as we feel the effect estimates and confidence intervals in the figure provide sufficient information.

Table 2 present response data, but does not show any treatment effect estimates or p-values. These might be helpful here.

The treatment effect estimates and p-values are in the results section of the manuscript. We would prefer not to duplicate these estimates in the table.

Figures 2a and 2b include legends saying that none of the median PSF2 times, nor their confidence limits, were estimable. Is this necessary?

We agree that this could be considered unnecessary and have removed this from the body of the plot and made note in the figure legend.

***** Reviewer #2:**
We thank reviewer 2 for considering our manuscript and are pleased they found our study results to be important and worthy of publication to a wide audience. We have responded to each comment below.

The manuscript by Jackson et al describes the first interim analysis of one of the randomisations on the UK Myeloma XI trial. The randomisation compared KRdc against CRD and CTD (2:1:1) with the latter two arms also undergoing a second randomisation in the event of suboptimal response. The design is thus complex with numerous caveats in interpretation of the results and the control arms (CTD or CRD) are not considered standard induction in most countries where proteasome inhibitor-based induction (+/- IMiD) is routine. Nonetheless, this is an important study and needs to be published to a wide audience. The PFS benefit for the KRdc arm is not only statistically significant but is clinically significant as well. The results are likely to be of interest to readers of Plos Medicine.

Major points
*    Methods (p6). It is hopefully a typo, but PFS is defined as the time from maintenance randomisation until progression or death. Please confirm if this definition is correct (rather than from induction randomisation). If it is correct, then results really need to be reanalysed with PFS defined according to correct definition. Same as per OS - please define accurately in view of above point.

This was indeed a typo, PFS is defined as the time from initial randomisation. We apologise for the confusion and thank the reviewer for spotting this error. This statement now reads (Page 8):

“Progression-free survival was defined as the time from randomisation to progressive disease or death from any cause.”

*    Methods (p7). Is it valid to combine CTD and CRD into one comparative group? In describing the primary endpoint analysis, the Protocol (Section 15.3) states that "To compare RCD with CTD, CCRD with RCD/CTD combined or with whichever of these two is superior" which is not exactly what is communicated in the Methods section. In the Study Design and Participant section of this paper it states that PFS and OS were significantly better for CRD compared to CTD. In the Results section an exploratory analysis of PFS in KRdc compared to both CRD and CTD induction when analysed separately is presented but this analysis is not mentioned or defined in the statistical section. Ultimately that data appears reasonable as both analysis types are presented for readers but the rationale to justify why the CRD and CTD arms can be compared into one group as opposed to performing comparisons of three separate arms, and the analysis method for the three group comparison needs to be tidied up in the statistical methods section.

Section 14.3 of the protocol and the study statistical analysis plan define the primary analysis to be against the combined triplets and exploratory analysis versus the individual triplets. This is why we have presented that data as shown so that the reader can see both the comparison with the combined triplet control as well as the outcome of each of the triplet controls individually. We have clarified this at the beginning of the statistical analysis section (Page 10).

Minor points
*    Methods (p5). Why were calcium and platelets chosen as minimisation factors in the randomisation as opposed to albumin which is a key prognostic variable in the ISS? Was the lased on some prior findings from the UK Myeloma group?

The choice of minimisation factors was based on the outcomes of the preceding MRC Myeloma VII and Myeloma IX trials.

*    Methods (p5). Is it valid to compare treatment arms with different dose densities and cycle numbers? The dexamethasone dose in the CTD is greater because of the shortened cycle length and the CTD and CTD arms have more cycles, presumably due to worse response rates. This results in treatment arms that are not directly comparable. Nonetheless, at least in terms of response and PFS efficacy, this would bias against the KRdc.

The protocol states that patients should be treated to maximum response at the discretion of the investigator before progressing to mobilisation. This strategy was also used in our previous trials such as MRC Myeloma IX. The constituents of the intervention are clearly described and could be considered as induction “treatment schedules” to be compared, rather than a comparison of dose densities.

*    Methods (p6). What was the definition of renal insufficiency for using MEL200? Please list whether eGRF cut-off or up to investigator discretion

This has been added to the methods (Page 8):

“…melphalan 200g/m2 (except in those with renal insufficiency defined as serum creatinine >= 200umol/L prior to transplant, 140mg/m2)”

*    Methods (p7). Which group of patients had MRD assessments performed? Only patients in CR? All patients?

Patients in all response groups had MRD assessments and were included.

*    Methods (p7). What methods were used for serum FLC and urine light chain excretion?

This has been added to the methods which now read (Page 9):

“Paraprotein (Sebia, France) and, serum-free light-chain analysis (Freelite, The Binding Site Ltd., Birmingham, UK) were assessed at least every 2 months for the first 2 years and then at least every 3 months until disease progression. Urine light chain excretion (Sebia, France) was assessed to confirm complete response.”

*    Methods (p7). Why were AEs not assessed in the CTD group?

We apologise for this error in the methods. As can be seen in Table 3 AEs were collected in all groups. This has now been amended and reads (Page 9):

“Adverse reactions were assessed at the start of each treatment cycle”

*    Results (Supplementary Fig 1). In the consort diagram it is not clear what has happened at various points as the numbers do not add up e.g. 511pts received KRdc but only 474 had response recorded. What happened to the others e.g. death, off study for toxicity etc. Similarly, it would be helpful to have the broad reasons for not proceeding to ASCT listed in this Figure.

We have made some additions to the CONSORT diagram for clarity.

*    Results (p9). Patients older than 65 had a greater PFS benefit with KRdc. This is somewhat unexpected. Can the authors speculate in the discussion why this might be the case?

We hypothesise that the inclusion of individual patients over the age of 65 in the transplant eligible pathway of the study may have been subject to a more rigorous assessment of fitness by the local clinicians than in younger patients. This may have enabled them to derive more benefit from the quadruplet combination. This is speculation and we do not have data to support this as local investigators were not required to justify their choice of treatment pathway. We therefore prefer not to include this in the discussion which is already quite long.

*    Results. While OS data is not mature, can the authors please provide the number of deaths in each arm.

This has been added to the Results, efficacy section (Page 11):

“Disease progression or death occurred in 411 patients (171 of 526 patients [32.5%, of which 101/526, 19.2% progression and 70/526, 13.3% death] in the KRdc group and 240 of 530 patients [45.3%, of which 151/530, 28.5% progression and 89/530, 16.8% death] in the control group).”

*    Results (p12). The cardiac event rate in the KRdc arm is very low. It seems that exclusion of patients with a history of cardiac disease was largely at investigator discretion. Perhaps this could be mentioned either in the Methods or Discussion as there will be some uncertainty about the extent of exclusion of patients with a history of cardiac disease.

This is an interesting point and we have added a statement to the section of the discussion where the cardiac event rate is discussed (Page 17).

“…overall incidence of cardiac adverse events was ow which may be related to the different dose of carfilzomib used in our study (36mg/m2 vs 56mg/m2) or the absence of the impact of prior therapy. The exclusion of patients with a significant history of cardiac disease was at the discretion of the investigators and so may have affected this finding.”

*    Results (Table 1). It appears that molecular risk was only known for ~ 35% of patients which is disappointing. It makes the conclusions about the benefit of KRdc in high and ultra-high risk myeloma harder to support e.g. in Supplementary Fig 2, while HRs are consistent with primary analysis, there is in fact no significant difference between arms for any of the cytogenetic risk groups, particularly not the ultra-high-risk group. While this is probably due to small numbers, it could also be because there is no beneficial effect of KRdc in the ultra-high-risk group. In a similar fashion, the conclusion states that there is promising activity in patients with t(4;14) and 17p- patients but no data in the Supplementary appendix is provided to support this (the high-risk curved in Suppl Fig 2 are overwhelmed by 1q patients). Could data on the t(4;14) and 17p-  groups be presented in the Supplementary appendix, acknowledging that numbers will be very small?

Data for the t(4;14) and del(17p) groups are included in the Forest plot (**Figure 2B**) and show a HR of 0.4 (0.17, 0.92) for patients with t(4;14) and of 0.14 (0.04, 0.54) for patients with del(17p). We have also added the Kaplan Meier curves for these groups to the Supplementary Appendix (**Supplementary Figure 2**).

*    Results (Table 2). The post-ASCT response rates appear to be per-protocol and not IIT. Could this be clearly indicated? This has been added to the table.

***** Reviewer #3:**
We thank reviewer 3 for their kind review of our study and manuscript. We are pleased they found our manuscript to be of high quality overall and worthy of publication. We have incorporated their suggestions to improve our report further.

I congratulate the authors on their thoughtful study design and well-written manuscript.  Unfortunately the study's impact will be limited primarily by the inclusion of CTD-treated patients in the control arm, as thalidomide has largely become antiquated due to its inferior efficacy and tolerability issues and is now only rarely used, at least in the United States.  Of course this is not the fault of the authors as the study was initiated prior to the de facto retirement of thalidomide.  What I found most thought provoking about this study was the signal of high efficacy in the high-risk subgroup.  Although the point of the study was to evaluate the value of adding carfilzomib to an IMiD-Cytoxan triplet, the more relevant question that the study raises in my opinion is the value of adding Cytoxan to an IMiD-PI triplet (or even, very ambitiously and probably unwisely, to an IMiD-PI-mAb quad) in patients with high-risk NDMM.  The EVOLUTION study attempted to answer the
question regarding the value of an IMiD-PI-Cytoxan quad vs triplets and failed to demonstrate a benefit of adding Cytoxan to VRD, however the overall number of subjects was low to the point of precluding any meaningful subgroup analysis.  Certainly the Myeloma XI+ high-risk subgroup analysis is not statistically significant but the HR in the 17p- subgroup for example is enough to give one pause.

It is also noteworthy that the high-grade hematologic toxicity in the KRDc arm was lower than what I would have expected.  The EVOLUTION investigators chose a Rev dose of 15mg for this reason, thinking that 25mg would be too toxic hematologically in a VRDc quad, and it seems that Myeloma XI+ may have proven otherwise, albeit with a relatively few number of induction cycles.

Although this was not a study of a combined IMiD-PI vs planned sequential IMiD->PI induction approach, the results nonetheless confirm the power of IMiD-PI synergy and suggest that a response-adapted approach that separates IMiD from PI and attempts to deepen response to the former with a switch to the latter is likely not an effective strategy.

Accordingly, I do think that the Myeloma XI+ trial is worthy of publication in this journal as it will make a meaningful contribution to the existing body of knowledge in the NDMM space.  I think the manuscript is high-quality overall but can be improved further:

1) "This is the first phase III randomised study of a carfilzomib and lenalidomide combination induction therapy for TE NDMM patients."  Is this completely accurate?  What about ENDURANCE and FORTE?

We apologise and did not wish to omit the significant contribution to the field of these studies, unpublished at the time of initial submission of our manuscript. The results of ENDURANCE, now published, and a reference to the early data from FORTE, have been added to the discussion. We have removed the statement ‘the first’.

2) Page 11 paragraph 2:  The inclusion of reasons for early stoppage of induction therapy in both arms is appreciated.  I am assuming that the definition of "early" is before completion of the fourth cycle.  I am curious as to the reasons for stopping induction therapy in general (not just stopping therapy early) in both arms.  Since median # of induction cycles was two cycles fewer in the KRDc arm and the ≥VGPR rate was higher in this arm, it is most likely that the latter is the explanation for the former, in other words the quad-treated subjects achieved maximal response with fewer cycles compared to those treated with a triplet.  However it is also possible that some subjects may have stopped KRDc after the fourth cycle due to toxicity and prior to the achievement of maximal response (aka prior to M-spike plateau in patients who hadn't yet achieved CR).  In order for this study to retain maximal significance in the current era (and specifically thinking about the coming IMiD-PI-mAb quad induction era), it would be nice to have a better idea of how tolerable KRDc is beyond four cycles.

We have added further information about the tolerability of treatment, including information about those stopping early (as previously) but adding information about all patients’ reasons for stopping induction. There is no evidence to suggest patients receiving KRdc stopped either early or after the 4^th^ cycle due to toxicity in higher rates than those receiving either triplet.

The sections now reads (Page 13):

“Induction treatment was planned for a minimum of four cycles and to continue to maximum response. The median number of treatment cycles received was 4 (range, 1 to 12) in the KRdc group and 6 (1 to 15) in the control group (CRD median 5, CTD 6). The use of a dose modification schedule in the event of adverse events allowed the vast majority of patients to complete at least four cycles of induction therapy. Patients not completing four cycles numbered 50 (9.5%) in the KRdc group and 43 (8.1%) in the CTD/CRD control group (CRD 20, CTD 23). The most common reasons cited for stopping induction therapy early were clinician choice (KRdc 22 patients, CTD/CRD control 26 patients, CRD 12, CTD 14) or unacceptable toxicity (KRdc 20 patients, CTD/CRD control 14 patients, CRD 4, CTD 11); more than one reason could be cited per withdrawal. Overall the majority of patients stopped induction therapy as they had reached maximum response as per protocol. Maximum response was cited as the reason for stopping in 430/526 (81.7%) in the KRdc group and 413/530 (77.9%) in the CTD/CRD control group (CRD 216, CTD 197). Clinician choice was cited in 37/526 (7.0%) in the KRdc group and 61/530 (11.5%) in the CTD/CRD triplet control group (CRD 24, CTD 37). Unacceptable toxicity was cited in 25/526 (4.8%) in the KRdc group and 26/530 (4.9%) in the CTD/CRD triplet control group (CRD 7, CTD 19).”

3) Page 11 paragraph 3:  "Thromboembolic events occurred in similar proportions of patients across the treatment groups."  This statement is probably true vis a vis the KRDc arm (9.4% DVT+other thrombosis) vs CRD/CTD control arm (7.5%), but probably not in terms of the KRDc arm vs the CRD arm specifically (5.8%).  We know that Thal is quite thrombogenic, and based on data such as the recently presented MSKCC data (Piedra et al, ASH 2019 abstract #1835) we now know that KRD is more thrombogenic than RVD (VTE rate 4x higher in patients on ASA prophylaxis!).  In my opinion, the statement of "similar proportions" may give the reader the impression that carfilzomib does not add thrombotic risk to a Revlimid-based regimen when we have a pretty good idea that it does and when the Myeloma XI+ data describe an almost 10% incidence of thrombosis with KRDc.  In my opinion, the portion of this manuscript that remains most relevant today is the KRDc vs CRD analysis, which the authors do a nice job of in terms of efficacy, but I would kindly suggest maybe something similarly robust in terms of toxicity as well.

We have reworded the section around VTEs and referenced our recently published paper which examines this in much more detail across the whole trial.

This section now reads (Page 14):

“Thromboembolic events occurred in slightly higher rates in patients receiving KRdc compared to Rdc. Thromboembolic events within the study have been examined in more detail in another manuscript.”

4) Page 13 paragraph 2: If the "potentially greater activity of carfizomib compared to bortezomib" is to be mentioned, I think there probably should be at least a brief discussion of ENDURANCE in the discussion section.

We have added a discussion of ENDURANCE to the manuscript. This section now reads (Page 16):

“Importantly, exposure to proteasome inhibitor and immunomodulatory agent combinations has been reported to be beneficial for these subgroups of patients and potentially the greater activity of carfilzomib compared to bortezomib is associated with these excellent results. The superiority of carfilzomib compared to bortezomib was demonstrated in patients with relapsed disease in the ENDEAVOR study. It is not, however, supported by data from the recently reported ENDURANCE study which examined the combination of carfilzomib with lenalidomide and dexamethasone (without cyclophosphamide) as induction treatment for newly diagnosed myeloma patients. This study did not identify a difference in outcome between patients receiving KRd or VRd but it was not carried out in the same population as Myeloma XI+, excluding patients with high-risk disease and those destined for stem cell transplantation. For transplant eligible patients of all risk subgroups the phase 2 FORTE study has reported preliminary data suggesting the KRd combination can achieve deeper responses than the combination of carfilzomib, cyclophosphamide and dexamethasone but full results are awaited.”

Congratulations again on an overall excellent manuscript.
